# Lewis acidity of a high-valent copper(iii) acetylacetonate

**DOI:** 10.1039/d6ra04158b

**Published:** 2026-07-08

**Authors:** Vladimir Motornov, Niklas Limberg

**Affiliations:** a Freie Universität Berlin Fabeckstraße 34-36 14195 Berlin Germany cuprate51@gmail.com motornov@zedat.fu-berlin.de

## Abstract

The Lewis acidity of the stable high-valent copper complex (acac)Cu^III^(CF_3_)_2_ was studied using experimental and computational methods. Pentacoordinated adducts formed *via* the addition of monodentate Lewis bases (Py, *i*PrNH_2_, MeCN, DMF, and Et_3_PO) were characterized, including using X-ray crystallography. Addition of a primary amine alters the acetylacetonate coordination. Coordination of a square-planar Cu(iii) complex with various N- and O-based ligands weakens the covalent Cu–C bonding, as revealed by NBO studies. The quantitative Lewis acidity of this compound was studied using experimental methods (Gutmann–Beckett) and DFT calculations of the fluoride ion affinity (FIA). The acidity was compared with those of copper(ii) and copper(i) acetylacetonates, which indicated a stepwise increase in Lewis acidity with the formal oxidation state of copper.

## Abstract 2

Lewis acidity of a stable high-valent copper(iii) compound has been systematically studied by X-ray characterization of the elusive adducts and DFT calculations.

## Graphical abstract

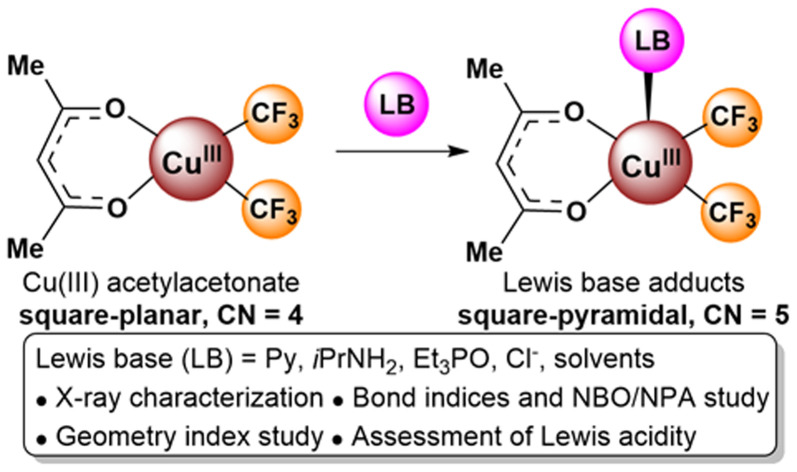

## Introduction

Copper in its high oxidation state of +3 is a unique species with a very high effective nuclear charge density for a transition metal. Over the past two decades, studies on Cu(iii) complexes have reshaped the field of transition metal chemistry, advancing the understanding of metalloenzyme oxidation,^1^ aerobic processes,^2^ and cross-coupling reactions.^3^ Well-defined, formally Cu(iii) trifluoromethyl complexes also find application in late-stage functionalization of complex molecules and biomolecules.^4^ Very recently, we have reported a tetrameric copper(iii) hydroxide scaffold as an easily accessible copper(iii) source,^5^ which enables direct access to a family of well-defined copper(iii) complexes with two trifluoromethyl groups, including chloride,^6*a*^ azide,^6*a*^ amino^6*a*^ complexes, scorpionates,^6*b*^ and 1,3-diketonates^5,7^*via* simple ligand exchange.

**Fig. 1:**
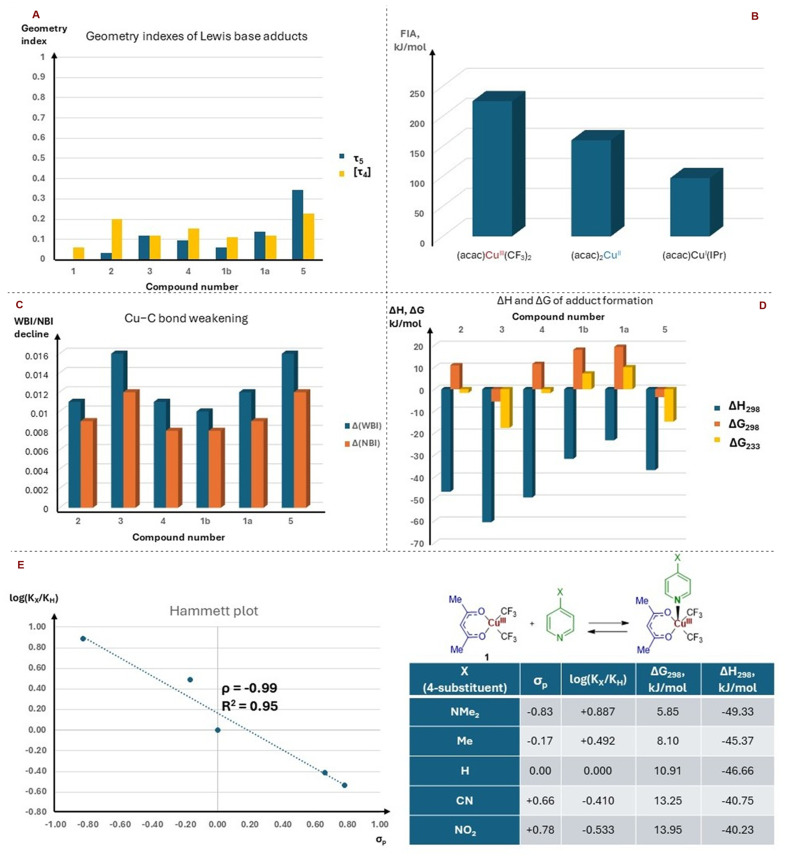
Calculated geometry indices for Lewis base adducts (A), calculated fluoride ion affinities for copper(iii), copper(ii) and copper(i) acetylacetonates (B), calculated declines in the NBO parameters (WBI and NBI) for Cu–C bonds (C) and Δ*H*_298_ and Δ*G* (at 298 and 233 K) of the Lewis base adducts formation (D) in DCM solution (calculated using the SMD solvation model), and the electronic effects of the modelled interaction between acetylacetonate 1 and different pyridines in DCM, illustrated by the Hammett plot (E). 1 – free (acac)Cu(CF_3_)_2_, 2 – Py adduct, 3 – *i*PrNH_2_ adduct, 4 – Et_3_PO adduct, 1b – DMF adduct, 1a – MeCN adduct, and 5 – chloride adduct. See SI for full calculation details.

The concept of Lewis acidity is fundamental to the understanding of chemical bonding, reactivity, and electronegativity.^8^ Although the Lewis acidity of main group elements in high oxidation states is well-established and has been extensively studied, especially in recent years,^9^ the situation is more complex for transition metals, where partially filled d-orbitals and the ambiguity of metal oxidation states introduce significant challenges in both experimental and theoretical assessments.^10^ The oxidation state of high-valent copper complexes is a subject of debate, where arguments for both Cu(iii) and inverted ligand field Cu(i) assignments have been made.^10^

**Scheme 1:**
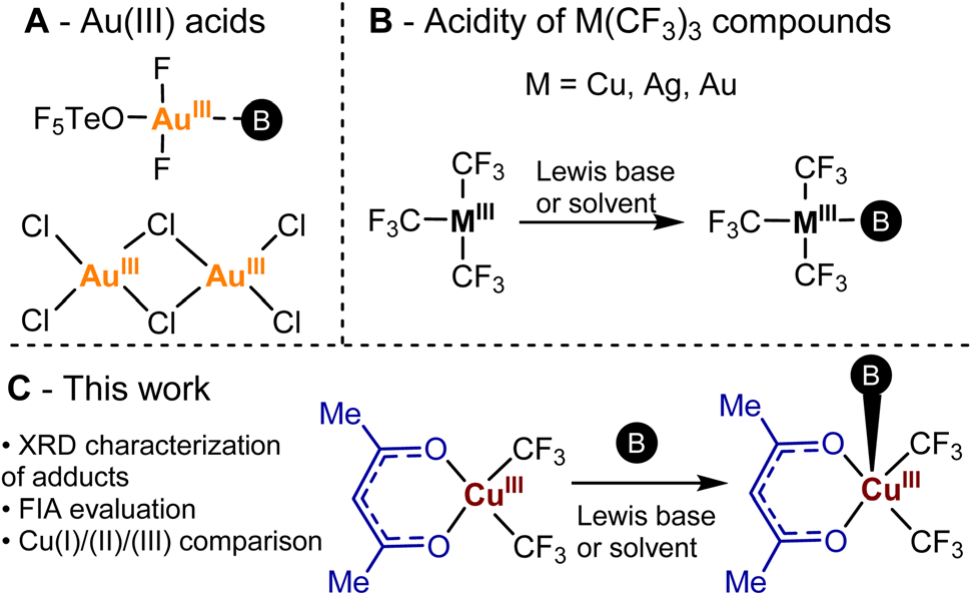
Reported gold(iii)-based Lewis acids (A), trifluoromethyl-containing Lewis acids (B), and this work (C).

In recent years, only the Lewis acidity of Au(iii), a 5d coinage metal with a more stable high oxidation state, has been experimentally studied in detail (Scheme 1A).^11^ Computational evaluation of fluoride ion affinity has been performed for high-valent 4d and 5d metal derivatives.^12^ Due to its high effective nuclear charge density, the Lewis acidity of Cu(iii) is particularly pronounced, even in stable square-planar complexes. Thus, the coordination number of five is as common as square-planar geometry for high-valent copper. Very recently, the formation of Lewis base adducts was observed for Cu(CF_3_)_3_ complexes by Nebra,^13^ Shen,^14^ and our group,^15^ and for Ag(CF_3_)_3_ complexes by the Menjon and Nebra groups (Scheme 1B).^16^ However, a detailed assessment of Lewis acidity or comprehensive theoretical investigations of transition metal-based acids still remain challenging. We have recently evaluated the Lewis acidity of neutral copper(iii) complexes including the novel Cu(iii) tetrameric hydroxide.^17^ The reactivity and composition of the Cu(iii)-Lewis base adducts varied depending on the base, which complicated a straightforward assessment. In contrast, the Lewis acidity of copper(iii) diketonates^5,7^ is completely unknown to date. Taking into account the suggested essential role of copper Lewis acidity in enzymatic processes,^18^ we envisioned the mononuclear (acac)Cu(CF_3_)_2_ (1) as an excellent platform to study the Lewis acidity of copper(iii), based on the straightforward formation of apically-coordinated 1 : 1 monoadducts. Moreover, the existence of various copper(i) and copper(ii) diketonates can enable a more straightforward comparison of the Lewis acidity of acetylacetonates of copper in different oxidation states. Herein, we report the reactivity of a bench-stable square-planar copper(iii) acetylacetonate with different nitrogen and oxygen Lewis bases, analyse the geometry and bonding alteration at the copper(iii) centre upon the Lewis base addition, and assess the Lewis acidity of copper(iii) by experimental and computational methods (Scheme 1C).

## Results and discussion

**Scheme 2:**
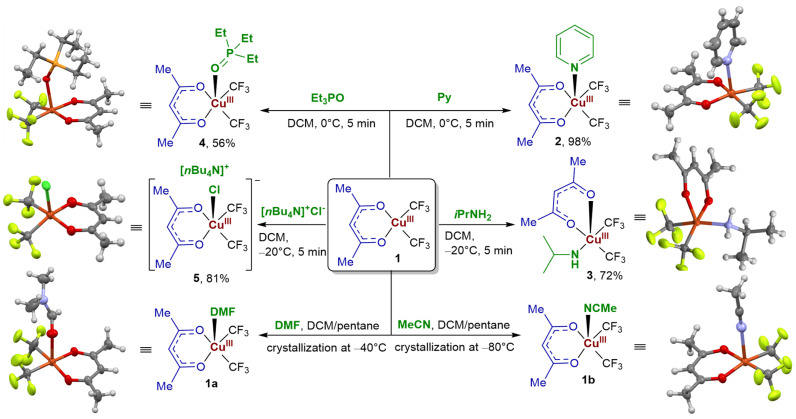
Synthesis and crystal structures of Lewis base-Cu(iii) acetylacetonate adducts 2–5, and solvates 1a–1b. Cation in the crystal structure of 5 is omitted for clarity. Ellipsoids are shown at the 50% probability level. Bond lengths (Å): 1: Cu–O: 1.879(3) and 1.865(2), Cu–C: 1.960(6) and 1.947(4); 2: Cu–N = 2.366(4), Cu–O = 1.883(3) and 1.897(3), Cu–C = 1.937(5) and 1.941(5); 3: Cu–N = 1.987(2), Cu–O = 2.187(1) and 1.880(1), Cu–C = 1.957(3) and 1.931(3); 4: Cu–O(apical) = 2.254(2); Cu–O(acac) = 1.893(1) and 1.892(2); Cu–C = 1.948(2) and 1.945(3); and 5: Cu–Cl = 2.501(1), Cu–O: 1.901(4) and 1.915(4); Cu–C: 1.947(6) and 1.932(6). 1a: Cu–N = 2.422(2), Cu–O = 1.892(2) and 1.884(2); Cu–C = 1.957(2) and 1.958(3). 1b: Cu–O(DMF) = 2.322(4), Cu–O(acac) = 1.887(1) and 1.870(1), Cu–C = 1.947(2) and 1.934(3).

We initiated our studies of the Lewis acidity of copper(iii) acetylacetonate 1 from its reactions with nitrogen Lewis bases (Scheme 2). For the synthesis of Lewis base adducts, direct reactions of complex 1 with the corresponding bases in dichloromethane at reduced temperatures were carried out. Thus, addition of either pyridine (a Lewis base with a lone pair on sp^2^-nitrogen atom) or isopropylamine (a stronger base with sp^3^ nitrogen donor) resulted in a change of the solution color to an intensive red. Crystallization at low temperature afforded the Lewis base monoadducts 2 and 3, respectively (Scheme 2). Importantly, pyridine is coordinated at the apical position with an elongated Cu–N bond of 2.366(4) Å, whereas the acetylacetonate coordination remains intact without considerable changes in the Cu–O and Cu–C bond lengths. In the case of isopropylamine, a change in the acetylacetonate coordination to an unsymmetrical mode is observed according to the crystal structure, where one Cu–O bond elongates to 2.187(1) Å in the apical position while the other remains short (1.880(1) Å). The amine is strongly coordinated (Cu–N = 1.987(2) Å) and lies on the same plane as the two CF_3_ ligands and the strongly coordinated arm of the acetylacetonate.

With these results, we turned our attention to acetonitrile, an even weaker base with sp-hybridization of nitrogen, which afforded the adduct 1a upon crystallization at −80 °C, with weakly coordinated acetonitrile (Cu–N = 2.422(2) Å). This copper–nitrogen distance is considerably longer than that observed for the adduct (MeCN)Cu(CF_3_)_3_, which reflects both the different geometry (square-planar *vs.* apical) and much stronger acidity of Cu(CF_3_)_3_ compared to diketonate 1. This solvate is unstable at room temperature and easily decomposes upon warming up.

With these adducts of acetylacetonate 1 with nitrogen Lewis bases, oxygen Lewis bases were tested. Crystallization of a DCM solution of 1 with the addition of DMF afforded a solvate 1b as orange crystals. Triethylphosphine oxide resulted in the formation of an adduct 4 as orange crystals with an apically coordinated ligand (Cu–O = 2.254(2) Å). Finally, tetrabutylammonium chloride as an anionic base was tested, which afforded an adduct 5 (Cu–Cl = 2.501(1) Å) in 91% yield after crystallization.

Importantly, solvates 1a and 1b require stabilization at low temperature, whereas all other adducts are stable in solution at room temperature. For the pyridine adduct 2, the dependence of the chemical shifts of the H4 pyridine atom was compared to that of free pyridine to estimate the degree of dissociation in solution (Table 1). Importantly, the H4 proton of pyridine shifts downfield, reflecting the electronic effects of the Lewis acid–base interaction. The downfield shift upon coordination Δ*δ*(H4) varies from 0.137 at ambient conditions to 0.212 at −75 °C, suggesting partial dissociation of the adducts in solution, leading to broadening of the NMR signals of the apical ligand. Importantly, no coalescence or shift to the slow exchange mode at low temperatures was observed, as only one set of signals was observed at all temperatures, which confirms that the process is very fast on the NMR scale.

**Table 1:** Temperature-dependent chemical shifts of pyridine protons in adduct 2 in a 0.1 M CD_2_Cl_2_ solution

*T*, K	*δ*(H4 (Py)), ppm [free Py: 7.675 ppm]	Δ*δ*(H4) ppm
198	7.887	0.212
213	7.874	0.199
228	7.862	0.183
243	7.852	0.173
258	7.837	0.158
273	7.824	0.145
288	7.812	0.137
303	7.812	0.137

From the crystal structures of all Lewis base adducts, we compared the change in the square-planar geometry of 1 (*τ*_4_ = 0.059) upon addition of a Lewis base, considering both *τ*_5_ index for square-pyramidal complexes, and the change in the original square-planar environment of copper with acetylacetonate and two CF_3_ ligands, without considering the entering Lewis base. The latter metric was named [*τ*_4_]-index, since it was calculated for the square-pyramidal complexes the same way as *τ*_4_ of 1 to reflect only the geometry of the pyramid base.^19^ The results (Table 2 and Fig. 1A) indicate distortion of the original geometry and slight changes in the geometry indices in all cases. The most prominent changes were observed for the amine adduct 3, where the coordination of acetylacetonate changed to unsymmetrical, and the chloride adduct 5 (the latter case can be attributed to the π-donor properties of chloride). The NPA charge on copper in acetylacetonate 1 is similar to that calculated for related copper(iii) species^5,6,20^ and higher than that calculated in copper(ii) complexes.^21^ Calculation of NBO parameters of the adducts revealed that the NPA charge on copper increases upon addition of a base (Table 2). While this might seem counter-intuitive, this result is likely to be a consequence of weakening of the π-backbonding of the Cu–CF_3_ coordination. This was confirmed by a decrease in both the Wiberg Bond Index (WBI) and Natural Binding Index (NBI) of Cu–C bonds upon addition of the Lewis base (Fig. 1B). This observation suggests much stronger π-backbonding in the square-planar environment of 1. The Cu(iii)-Lewis base interactions are predominantly ionic with a low degree of covalency, compared to the Cu–C coordination. The highest WBI and NBI parameters were observed in the case of amine complex 3, which corroborates the crystal structure data. Validation of these results using different functionals (B2PLYP and coupled-cluster DLPNO-CCSD(T)) supported these trends (see SI for full details). Moreover, *T*_1_ factor of 0.018–0.020 calculated for all complexes 1–5 at the DLPNO-CCSD(T)/def2-TZVP level lies safely below the 0.050 benchmark for transition metal systems,^22^ which validates the use of single-reference approaches for these formally copper(iii) compounds.

**Table 2:** Geometry indices and NBO parameters for acetylacetonate 1 and its Lewis base adducts^a^

Complex (base)	*τ*_5_	*τ*_4_ or [*τ*_4_]	NPA (Cu)	WBI (Cu–L)^b^	NBI (Cu–L)^b^	WBI (Cu–C) median	NBI (Cu–C) median
1 (none)	—	0.059	1.119	—	—	0.529	0.728
2 (Py)	0.033	0.139	1.178	0.051	0.226	0.518	0.719
3 (*i*PrNH_2_)	0.117	0.200	1.162	0.182	0.426	0.513	0.716
4 (Et_3_PO)	0.095	0.153	1.191	0.046	0.213	0.518	0.720
1b (DMF)	0.059	0.110	1.187	0.042	0.206	0.519	0.720
1a (MeCN)	0.138	0.117	1.172	0.041	0.203	0.517	0.719
5 (Cl^−^)	0.343	0.228	1.179	0.121	0.347	0.513	0.716

a Geometry indices were calculated from the XRD structures; NBO parameters were calculated at the ωB97X-D3(BJ)/def2-QZVPPD//ωB97X-D3(BJ)/def2-TZVPD level of theory.

b Cu–L indicates a bond with a new Lewis base.

With these results, the Lewis acidity trends of acetylacetonate 1 were investigated. The ^31^P NMR spectrum of the triethylphosphine oxide adduct 4 in acetonitrile at room temperature showed only a slight downfield shift to 53.0 ppm, compared to 50.0 ppm for the pure base (see SI for full details). This indicates a relatively weak experimental Lewis acidity of compound 1 at ambient conditions, which could be even more diminished in a coordinating solvent. The dynamic behavior of the adducts in solution led to broadening of the signals when non-coordinating solvents were used, which complicated the experimental assessment. Theoretically, the fluoride ion affinity (FIA) was established as the most reliable indicator of Lewis acidity of hard main group Lewis acids.^9^ Thus, we performed FIA calculations for acetylacetonate 1 using two methods, relying either on the TMSF/TMS^+^ anchor point approach at the ωB97X-D3(BJ)/def2-TZVPPD//B3LYP-D3(BJ)/ZORA-def2-TZVP level of theory (Greb's method)^9*a*^ or on the total enthalpy change at the ωB97X-D3(BJ)/def2-TZVPD level of theory (Kaupp's method).^9*d*^ Considering the ambiguous oxidation state assignments due to the highly covalent Cu–C bonds, relativistic effects are important for taking into account the contraction of the 3d orbitals and the energy shifts of the 4s and 4p orbitals when the optimization is conducted at a lower level of theory (Greb's method). The inclusion of diffuse functions, as employed in Kaupp's method,^9*d*^ is essential for correct geometry optimization and accurate Cu–L bonds where no anchor point is used. The calculated FIA of acetylacetonate 1 (calculated in the gas phase, as is commonly done for main group Lewis acids)^9^ is 257 or 225 kJ mol^−1^, respectively, which is comparable to the calculated acidity of Ph_3_B.^23^ The discrepancy can be attributed to the difference between the electronic nature of the more complex copper(iii)-based Lewis acid 1 and the simple TMS anchor, as well as the lower sensitivity of the anchor-point method^9*a*^ to the basis set used. The value obtained *via* Kaupp's method^9*d*^ was compared with the Lewis acidities of the simple square-planar copper(ii) acetylacetonate calculated using the same method, which turned out to have a lower FIA of 160 kJ mol^−1^ (Fig. 1C). For a representative Lewis acidic copper(i) acetylacetonate, we selected a stable complex with an additional NHC ligand [Cu(acac)(IPr)],^24^ (IPr = 1,3-bis(2,6-diisopropylphenyl)imidazole-2-ylidene) which can be capable of Lewis base addition to form a stable tetrahedral Cu(i) adduct, typical for Cu(i). The calculated FIA of the formed complex further decreased to 97 kJ mol^−1^. This tendency reflects the stepwise increase in Lewis acidity from Cu(i) to Cu(ii) (by 63 kJ mol^−1^) and further to Cu(iii) (by another 65 kJ mol^−1^), supporting the treatment of formal oxidation states in copper complexes as distinct values, in line with the IUPAC formalism.^25^ While these are not physical oxidation states and the copper(i), (ii), and (iii) acetylacetonate systems are not completely identical, the observed trend is notable in distinguishing the true Cu(i) systems from entirely chemically distinct formal Cu(iii) systems.

With these insights, the enthalpies and Gibbs free energies of Lewis acid–base interactions in DCM solution were compared for all compounds at the ωB97X-D3(BJ)/def2-QZVPPD//ωB97X-D3(BJ)/def2-TZVPD level of theory with the SMD(DCM) solvation model to simulate the experimentally observed reactivity (Fig. 1D). The results indicate relatively low Gibbs energies of adduct formation, indicating relatively weak Lewis acid–base interactions. While the enthalpies of all Lewis base additions are slightly negative, the negative Δ*S* makes a significant contribution to the Gibbs free energy, which is negative only in the case of a strong base (primary amine) and the small chloride anion, where the entropic contribution is low.

The affinity of Lewis bases for the formal copper(iii) centre can therefore be formulated as decreasing in the following order: R–NH_2_ > Cl^−^ > Py > Et_3_PO > DMF > MeCN. Positive Gibbs energies of adduct formation calculated at 298 K reflect the experimental conditions necessary for the formation of these adducts, since low temperatures (down to −80 °C) were used for crystallization. The Δ*G*_233_ is negative for all adducts except the DMF and MeCN solvates, which are stabilized at lower temperatures, suggesting a weak binding. Under standard conditions (298 K), the acetylacetonate complex 1 does not form stable solvates, which is remarkably different from stronger acids, such as Cu(CF_3_)_3_ (ref. 8) and the cubane [Cu(CF_3_)_2_(OH)]_4_.^14^

To examine the electronic effects of the Lewis base on the copper(iii) acetylacetonate system more precisely, the thermodynamics of interaction between pyridines with different para-substituents in DCM and the parent complex 1 were modelled by DFT calculations (Fig. 1E). The Hammett plot revealed a moderate influence of the electronic properties of the Lewis bases on the equilibrium constant (*ρ* = −0.99 with a confidence value *R*^2^ = 0.95). The calculated binding enthalpy of the parent complex 1 in DCM solution for the most electron-rich 4-dimethylaminopyridine is approximately 20% higher than that of the most electron-deficient 4-nitropyridine.

## Conclusions

In conclusion, the Lewis acidity of the formal copper(iii) trifluoromethyl acetylacetonate has been systematically studied. Several representative square-pyramidal adducts of [(acac)Cu(CF_3_)_2_] with nitrogen, oxygen, and chloride Lewis bases have been isolated and fully characterized. The copper(iii) complex geometry change from square-planar to square-pyramidal has been analysed by bond index parameter and NPA charge calculations on different levels of theory, revealing the weakening of copper(iii)-ligand interaction upon addition of the Lewis base. The calculated fluoride ion affinity of this complex (225 kJ mol^−1^) is considerably higher than that of copper(ii) acetylacetonate and tricoordinate copper(i) acetylacetonate analogs, supporting increased Lewis acidity in formal copper(iii) complexes. The affinity of high-valent copper for Lewis bases, based on Gibbs free energy calculations in DCM solution, was characterized in the following order: R–NH_2_ > Cl^−^ > Py > Et_3_PO > DMF > MeCN.

## Author contributions

V. Motornov conceived the idea, led the project, acquired the funding, conducted the experiments and calculations, and wrote the manuscript. N. Limberg measured the X-ray crystal structures.

## Conflicts of interest

There are no conflicts to declare.

## Supplementary Material

RA-016-D6RA04158B-s001

RA-016-D6RA04158B-s002

RA-016-D6RA04158B-s003

RA-016-D6RA04158B-s004

RA-016-D6RA04158B-s005

## Data Availability

Data are available in the supplementary information (SI) of this article. Supplementary information: experimental procedures, characterization details and analytical data, computational details, copies of NMR spectra. See DOI: https://doi.org/10.1039/d6ra04158b. CCDC 2546501 (1), 2546502 (1a), 2546503 (2), 2546506 (3), 2546511 (4), 2546512 (5), and 2546600 (1b) contain the supplementary crystallographic data for this paper.^26*a*–*g*^
