# Supplementary material for: Lewis acidity of a high-valent copper(iii) acetylacetonate
Source: RSC Adv. 2026 Jul 8;16(36):38588–93. doi: 10.1039/d6ra04158b (PMC13343543; doi:10.1039/d6ra04158b)

Supplementary Information (SI) for RSC Advances.
This journal is © The Royal Society of Chemistry 2026

Supplementary information for

Lewis acidity of a high-valent copper(III) acetylacetonate

Vladimir Motornov[a],* and Niklas Limberg[a]

[a] Dr. V. Motornov,* N. Limberg, Freie Universität Berlin; Fabeckstraße 34-36, 14195 Berlin, Germany.
E-mail: cuprate51@gmail.com, motornov@zedat.fu-berlin.de

Table of contents

General 1
X-ray crystallography (general procedure) 2
Synthesis of pyridine adduct 2. 6
Synthesis of isopropylamine adduct 3 9
Synthesis of triethylphosphine oxide adduct 4. 13
Synthesis of chloride anionic complex 5. 17
Synthesis of solvates 1a and 1b 21
DFT calculations 28
References 32
NMR spectra 33

General

All solvents used for the reactions were HPLC grade. All commercially available chemicals were purchased from commercial suppliers (BLD Pharmatech, Sigma-Aldrich) and were used as received. Copper(III) bis(trifluoromethyl) acetylacetonate **1** was prepared according to the procedure reported by us previously (treatment of $[Cu(CF_3)_2(OH)]_4$ with acetylacetone).[1] Yields refer to isolated compounds. IR spectra were recorded on a Nicolet 6700 FTIR (Thermo Fisher Scientific) spectrometer. Elemental analysis was performed on the VARIO EL elemental analyzer. ^{1}H, ^{13}C, and ^{19}F NMR spectra were recorded on a JEOL 400 MHz spectrometer in the solvent indicated using 5 mm diameter NMR tubes; chemical shifts (δ) are given in ppm relative to the residual solvent peak. ^{13}C NMR spectra were proton decoupled. The chemical shift values (δ) are reported in ppm relative to Me_4Si (0 ppm for ^{1}H, ^{13}C NMR) or $CFCl_3$ (0 ppm for ^{19}F NMR). Coupling constants (J) are reported in Hertz.

X-ray crystallography (general procedure)

A crystal was mounted on a MiTeGen micromount with perfluoroether oil. Data were collected from a shock-cooled single crystal at 150.00 K on a Bruker D8 VENTURE dual wavelength Mo/Cu three-circle diffractometer with a microfocus sealed X-ray tube using a mirror optics as monochromator and a Bruker PHOTON II detector. The diffractometer was equipped with an Oxford Cryostream 700 low temperature device and used MoK_α radiation (λ = 0.71073 Å). All data were integrated with SAINT V8.41 and a multi-scan absorption correction using SADABS 2016/2 was applied.[2,3] The structure was solved by direct methods with SHELXT 2018/2 and refined by full-matrix least-squares methods against F^2 using XL.[4,5] All non-hydrogen atoms were refined with anisotropic displacement parameters. All hydrogen atoms were refined isotropic on calculated positions using a riding model with their U_{iso} values constrained to 1.5 times the U_{eq} of their pivot atoms for terminal sp^3 carbon atoms and 1.2 times for all other carbon atoms. Crystallographic data for the structures reported in this paper have been deposited with the Cambridge Crystallographic Data Centre.[6] CCDC 2546501 (**1**), 2546503 (**2**), 2546506 (**3**), 2546511 (**4**), 2546512 (**5**), 2546502 (**1a**) and 2546600 (**1b**) contain the supplementary crystallographic data for this paper. These data can be obtained free of charge from The Cambridge Crystallographic Data Centre via www.ccdc.cam.ac.uk/structures. The CIF files were generated using FinalCif software.[7]

Crystal structure of [(acac)Cu(CF_3)$_2$] 1

Single crystals of [(acac)Cu(CF_3)$_2$] were grown from a homogeneous solution of **1** (50 mg) in 9:1 pentane/DCM (0.5 ml) at −80°C for 24 hours. Dark yellow needles precipitated.

^{1}H NMR (400 MHz, $CDCl_3$) δ 5.65 (s, 1H), 2.11 (s, 6H); **^{13}C NMR** (101 MHz, $CDCl_3$) δ 189.6 (C=O), 101.8 (=CH), 26.8 (Me); **^{19}F NMR** (376 MHz, $CDCl_3$) δ −30.2 (s); Elem. Anal. calcd. for $C_7H_7O_2CuF_6$: 27.96% C, 2.35% H; found: 27.98% C, 2.18% H. Characterization details matched previously reported data.[1]

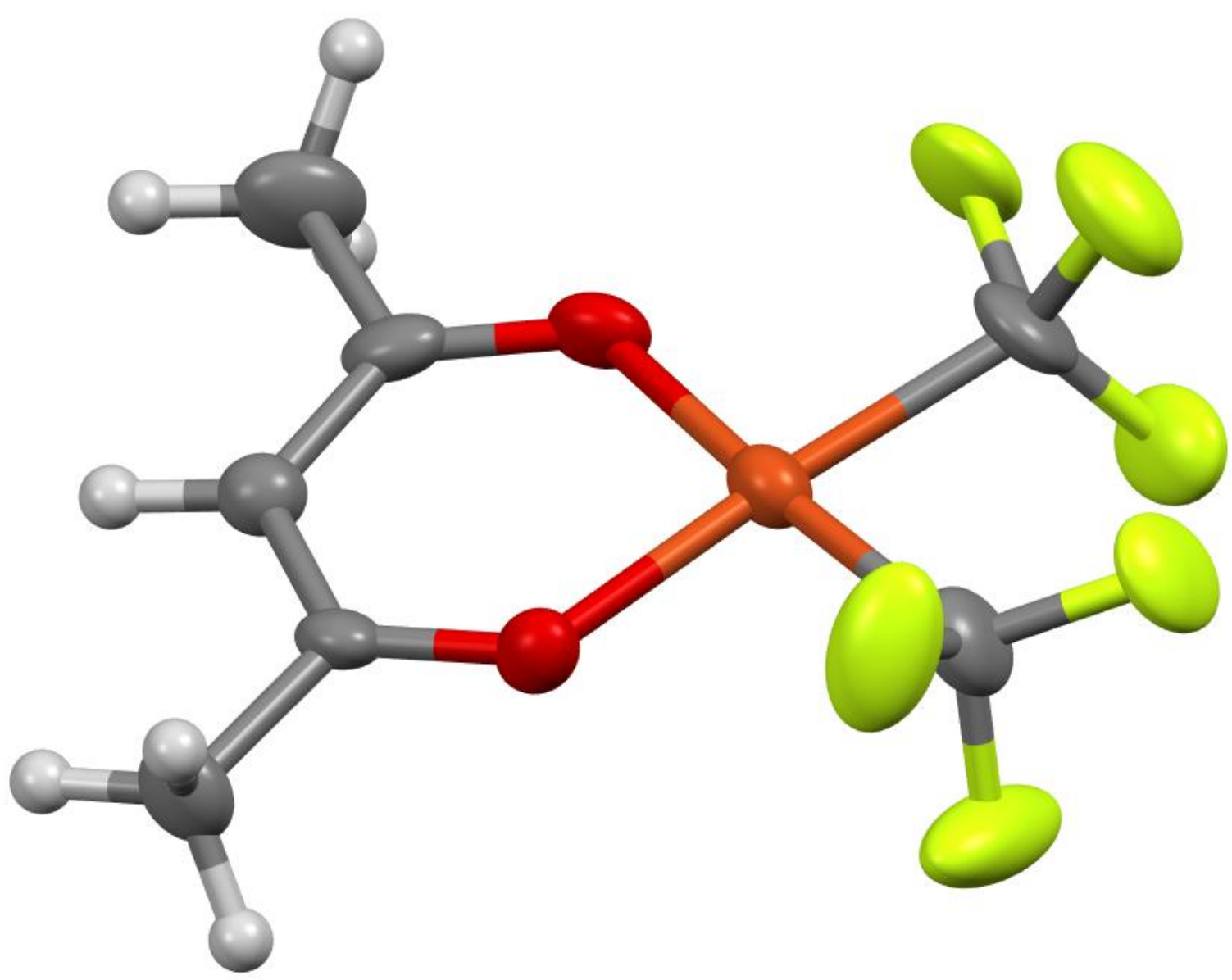

*Figure S1. Crystal structure of the parent [(acac)Cu(CF_3)$_2$] complex **1***

Table 1. Crystal data and structure refinement for 1

CCDC number	2546501
Empirical formula	$C_7H_7CuF_6O_2$
Formula weight	300.67
Temperature [K]	150.00
Crystal system	monoclinic
Space group (number)	$P2_1$ (4)
a [Å]	8.1514(8)
b [Å]	6.6328(5)
c [Å]	9.9108(9)
α [°]	90
β [°]	111.516(3)
γ [°]	90
Volume [Å^3]	498.50(8)
Z	2
ρ_{calc} [gcm^{-3}]	2.003
μ [mm^{-1}]	2.262
F(000)	296
Crystal size [mm^3]	0.111×0.161×0.74
Crystal colour	yellow
Crystal shape	needle
Radiation	MoK_α (λ=0.71073 Å)
2θ range [°]	4.42 to 50.72 (0.83 Å)
Index ranges	−9 ≤ h ≤ 9<br>−7 ≤ k ≤ 7<br>−11 ≤ l ≤ 11
Reflections collected	11823
Independent reflections	1822<br>R_{int} = 0.0583<br>R_{sigma} = 0.0353
Completeness to θ = 25.242°	99.9 %
Data / Restraints / Parameters	1822 / 88 / 204
Absorption correction T_{min}/T_{max} (method)	0.4397 / 0.7452 (multi-scan)
Goodness-of-fit on F^2	1.060
Final R indexes [I≥2σ(I)]	R_1 = 0.0253<br>wR_2 = 0.0587
Final R indexes [all data]	R_1 = 0.0305<br>wR_2 = 0.0627
Largest peak/hole [eÅ^{-3}]	0.29/−0.30
Flack X parameter	0.46(6)

Table 2. Bond lengths and angles for 1

Atom–Atom	Length [Å]
Cu1–O1	1.880(2)
Cu1–O2	1.865(3)
Cu1–C2	1.960(4)
Cu1–C1	1.947(5)
O1–C4	1.283(4)
O2–C6	1.274(5)
F5A–C2	1.348(13)
F4A–C2	1.286(13)
F6A–C2	1.357(7)
C6–C5	1.393(5)
C6–C7	1.492(5)
C4–C3	1.499(5)
C4–C5	1.388(5)
C3–H3A	0.9800
C3–H3B	0.9800
C3–H3C	0.9800
C5–H5	0.9500
C2–F4B	1.363(16)
C2–F5B	1.301(18)
C2–F6B	1.273(19)
C1–F3B	1.327(16)
C1–F2B	1.30(2)
C1–F1B	1.30(3)
C1–F2A	1.334(10)
C1–F1A	1.24(2)
C1–F3A	1.43(3)
C7–H7A	0.9800
C7–H7B	0.9800
C7–H7C	0.9800
**Atom–Atom–Atom**	**Angle [°]**
O1–Cu1–C2	175.9(2)
O1–Cu1–C1	89.11(15)
O2–Cu1–O1	94.93(11)
O2–Cu1–C2	89.12(17)
O2–Cu1–C1	175.8(3)
C1–Cu1–C2	86.8(2)
C4–O1–Cu1	125.3(2)
C6–O2–Cu1	125.8(2)
O2–C6–C5	125.3(3)
O2–C6–C7	114.2(4)
C5–C6–C7	120.4(4)
O1–C4–C3	114.6(3)
O1–C4–C5	125.1(3)
C5–C4–C3	120.2(3)
C4–C3–H3A	109.5
C4–C3–H3B	109.5
C4–C3–H3C	109.5
H3A–C3–H3B	109.5
H3A–C3–H3C	109.5
H3B–C3–H3C	109.5
C6–C5–H5	118.3
C4–C5–C6	123.5(3)
C4–C5–H5	118.3
F5A–C2–Cu1	112.0(9)
F5A–C2–F6A	102.1(12)
F4A–C2–Cu1	115.5(8)
F4A–C2–F5A	110.0(5)
F4A–C2–F6A	106.0(12)
F6A–C2–Cu1	110.3(4)
F4B–C2–Cu1	114.7(9)
F5B–C2–Cu1	103.9(11)
F5B–C2–F4B	106.6(13)
F6B–C2–Cu1	110.5(13)
F6B–C2–F4B	111.5(18)
F6B–C2–F5B	109(2)
F3B–C1–Cu1	122.2(8)
F2B–C1–Cu1	109.6(12)
F2B–C1–F3B	101.4(16)
F2B–C1–F1B	111.7(14)
F1B–C1–Cu1	106.8(13)
F1B–C1–F3B	104.9(18)
F2A–C1–Cu1	110.9(4)
F2A–C1–F3A	99.8(16)
F1A–C1–Cu1	120.2(12)
F1A–C1–F2A	111.7(17)
F1A–C1–F3A	106.4(12)
F3A–C1–Cu1	105.4(12)
C6–C7–H7A	109.5
C6–C7–H7B	109.5
C6–C7–H7C	109.5
H7A–C7–H7B	109.5
H7A–C7–H7C	109.5
H7B–C7–H7C	109.5

Table 3. Torsion angles for 1

**Atom–Atom–Atom–Atom**	**Torsion Angle [°]**
Cu1–O1–C4–C3	179.1(12)
Cu1–O1–C4–C5	−3(3)
Cu1–O2–C6–C5	−3(3)
Cu1–O2–C6–C7	178.8(14)
O1–Cu1–O2–C6	1.7(17)
O1–C4–C5–C6	2(3)
O2–Cu1–O1–C4	1.3(15)
O2–C6–C5–C4	2(3)
C3–C4–C5–C6	179.4(17)
C2–Cu1–O2–C6	−179.0(16)
C1–Cu1–O1–C4	−177.7(16)
C7–C6–C5–C4	179.6(19)

Synthesis of pyridine adduct 2.

To the solution of (acac)Cu(CF_3)$_2$ **1** (60 mg, 0.2 mmol) in dry dichloromethane (0.5 ml) pyridine (16.1 µl, 0.2 mmol) was added at room temperature. The mixture turned red immediately. The solution was concentrated under reduced pressure to give pure pyridine adduct **2** (74 mg, 98%). Crystallization from 2:1 pentane/DCM at −80°C for 2 days afforded orange-red crystals suitable for X-ray crystallography. **^{1}H NMR** (376 MHz, $CDCl_3$) δ 9.61 (br s, 2H, H2/H6(Py)), 7.87 (br s, 1H, H4(Py)), 7.70 (br s, 2H, H3/H5(Py)), 5.53 (s, 1H, =CH), 2.06 (s, 6H, 2Me); **^{19}F NMR** (376 MHz, $CDCl_3$) δ −30.3 (br s, 6F); **IR** (ATR): $\tilde{\nu}$ = 1600, 1580, 1526, 1385, 1361, 1146, 1086, 956, 929, 723, 704, 614 cm^{-1}; Elem. Anal. calcd. for $CuC_{12}F_6H_{12}O_2N$: 37.95% C, 3.69% N, 3.19% H, found: 38.09% C, 4.38% N, 2.99% H. **CCDC** 2546503.

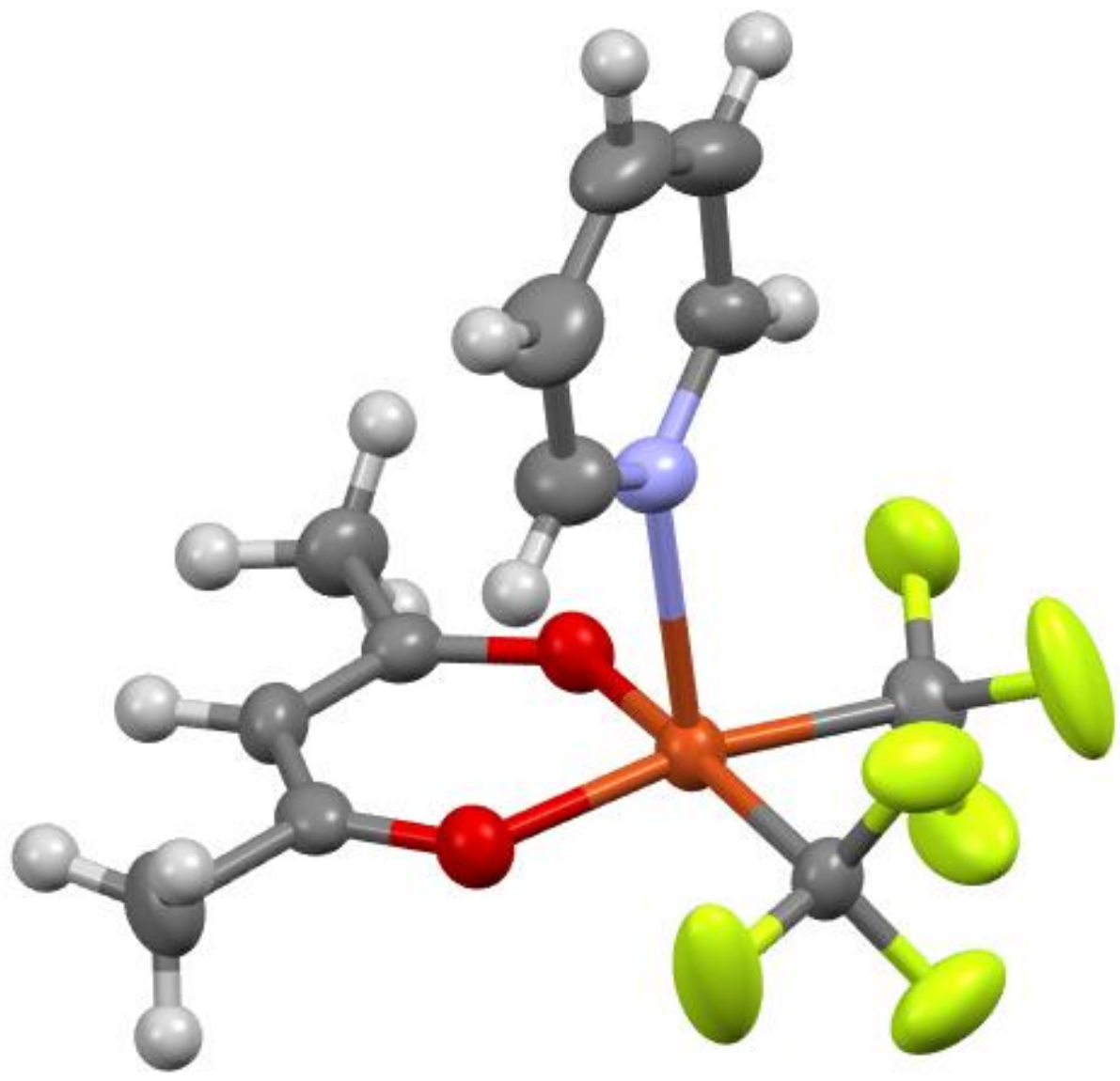

Figure S2. Crystal structure of [(acac)Cu(CF$_3$)$_2$(Py)] complex ***2***

Table 4. Crystal data and structure refinement for 2

CCDC number	2546503
Empirical formula	$C_{12}H_{12}CuF_6NO_2$
Formula weight	379.77
Temperature [K]	150.00
Crystal system	monoclinic
Space group (number)	$P2_1/c$ (14)
a [Å]	12.0638(7)
b [Å]	12.7245(8)
c [Å]	9.6927(6)
α [°]	90
β [°]	97.924(2)
γ [°]	90
Volume [Å^3]	1473.68(16)
Z	4
ρ_{calc} [gcm^{-3}]	1.712
μ [mm^{-1}]	1.552
F(000)	760
Crystal size [mm^3]	0.146×0.169×0.184
Crystal colour	orange
Crystal shape	block
Radiation	MoK_α (λ=0.71073 Å)
2θ range [°]	4.68 to 50.81 (0.83 Å)
Index ranges	$-14 \le h \le 14$ $-15 \le k \le 15$ $-11 \le l \le 11$
Reflections collected	64978
Independent reflections	2713 R_{int} = 0.1126 R_{sigma} = 0.0320
Completeness to θ = 25.242°	100.0 %
Data / Restraints / Parameters	2713 / 42 / 257
Absorption correction T_{min}/T_{max} (method)	0.5122 / 0.7452 (multi-scan)
Goodness-of-fit on F^2	1.257
Final R indexes [$I \ge 2\sigma(I)$]	R_1 = 0.0417 wR_2 = 0.0879
Final R indexes [all data]	R_1 = 0.0750 wR_2 = 0.1183
Largest peak/hole [eÅ^{-3}]	0.97/−0.61

Table 5. Bond lengths and angles for 2

Atom–Atom	Length [Å]
Cu1–O1	1.882(3)
Cu1–O2	1.897(3)
Cu1–N1	2.367(4)
Cu1–C1	1.937(5)
Cu1–C2	1.941(4)
O1–C4	1.282(5)
O2–C6	1.278(5)
F6A–C1	1.337(6)
F3A–C2	1.321(6)
F1A–C2	1.342(6)
F4A–C1	1.333(6)
F5A–C1	1.309(6)
N1–C8	1.337(6)
N1–C12	1.329(6)
C6–C5	1.382(6)
C6–C7	1.507(6)
C5–H5	0.9500
C5–C4	1.379(6)
F2A–C2	1.323(6)
C4–C3	1.505(6)
C1–F4B	1.358(18)
C1–F5B	1.302(19)
C1–F6B	1.316(18)
C2–F1B	1.277(16)
C2–F3B	1.333(19)
C2–F2B	1.364(15)
C7–H7A	0.9800
C7–H7B	0.9800
C7–H7C	0.9800
C8–H8	0.9500
C8–C9	1.374(7)
C3–H3A	0.9800
C3–H3B	0.9800
C3–H3C	0.9800
C12–H12	0.9500
C12–C11	1.388(7)
C10–H10	0.9500
C10–C11	1.367(9)
C10–C9	1.375(9)
C11–H11	0.9500
C9–H9	0.9500
**Atom–Atom–Atom**	**Angle [°]**
O1–Cu1–O2	95.16(13)
O1–Cu1–N1	91.23(14)
O1–Cu1–C1	169.18(18)
O1–Cu1–C2	88.49(16)
O2–Cu1–N1	91.37(13)
O2–Cu1–C1	88.53(16)
O2–Cu1–C2	171.17(17)
C1–Cu1–N1	98.86(17)
C1–Cu1–C2	86.49(19)
C2–Cu1–N1	96.59(17)
C4–O1–Cu1	124.5(3)
C6–O2–Cu1	124.2(3)

C8–N1–Cu1	118.0(3)
C12–N1–Cu1	124.7(3)
C12–N1–C8	117.2(4)
O2–C6–C5	125.7(4)
O2–C6–C7	114.7(4)
C5–C6–C7	119.6(4)
C6–C5–H5	117.9
C4–C5–C6	124.3(4)
C4–C5–H5	117.9
O1–C4–C5	125.7(4)
O1–C4–C3	113.8(4)
C5–C4–C3	120.5(4)
F6A–C1–Cu1	109.2(3)
F4A–C1–Cu1	110.0(3)
F4A–C1–F6A	104.4(4)
F5A–C1–Cu1	120.3(3)
F5A–C1–F6A	104.2(4)
F5A–C1–F4A	107.5(5)
F4B–C1–Cu1	111.4(15)
F5B–C1–Cu1	116.5(17)
F5B–C1–F4B	102(2)
F5B–C1–F6B	107(3)
F6B–C1–Cu1	111.5(16)
F6B–C1–F4B	108(2)
F3A–C2–Cu1	112.8(3)
F3A–C2–F1A	105.5(4)
F3A–C2–F2A	107.5(4)
F1A–C2–Cu1	115.1(3)
F2A–C2–Cu1	111.2(3)
F2A–C2–F1A	104.1(4)
F1B–C2–Cu1	112.4(12)
F1B–C2–F3B	107(2)
F1B–C2–F2B	105.6(15)
F3B–C2–Cu1	123.9(16)
F3B–C2–F2B	97(2)
F2B–C2–Cu1	108.9(8)
C6–C7–H7A	109.5
C6–C7–H7B	109.5
C6–C7–H7C	109.5
H7A–C7–H7B	109.5
H7A–C7–H7C	109.5
H7B–C7–H7C	109.5
N1–C8–H8	118.4
N1–C8–C9	123.1(5)
C9–C8–H8	118.4
C4–C3–H3A	109.5
C4–C3–H3B	109.5
C4–C3–H3C	109.5
H3A–C3–H3B	109.5
H3A–C3–H3C	109.5
H3B–C3–H3C	109.5
N1–C12–H12	118.3
N1–C12–C11	123.3(5)
C11–C12–H12	118.3
C11–C10–H10	120.5
C11–C10–C9	119.1(5)

C9–C10–H10	120.5
C12–C11–H11	120.8
C10–C11–C12	118.4(5)
C10–C11–H11	120.8
C8–C9–C10	118.9(6)
C8–C9–H9	120.6
C10–C9–H9	120.6

Table 6. Torsion angles for 2

**Atom–Atom–Atom–Atom**	**Torsion Angle [°]**
Cu1–O1–C4–C5	3.7(6)
Cu1–O1–C4–C3	−177.6(3)
Cu1–O2–C6–C5	−4.3(6)
Cu1–O2–C6–C7	176.5(3)
Cu1–N1–C8–C9	175.3(4)
Cu1–N1–C12–C11	−175.4(4)
O1–Cu1–O2–C6	7.0(3)
O2–Cu1–O1–C4	−6.8(4)
O2–C6–C5–C4	−1.3(7)
N1–Cu1–O1–C4	84.7(3)
N1–Cu1–O2–C6	−84.3(3)
N1–C8–C9–C10	−0.3(9)
N1–C12–C11–C10	1.1(8)
C6–C5–C4–O1	1.6(8)
C6–C5–C4–C3	−177.0(4)
C1–Cu1–O1–C4	−116.4(9)
C1–Cu1–O2–C6	176.9(4)
C2–Cu1–O1–C4	−178.7(4)
C7–C6–C5–C4	177.9(4)
C8–N1–C12–C11	0.0(7)
C12–N1–C8–C9	−0.4(8)
C11–C10–C9–C8	1.5(9)
C9–C10–C11–C12	−1.8(8)

Synthesis of isopropylamine adduct 3

To the solution of (acac)Cu(CF_3)$_2$ **1** (60 mg, 0.2 mmol) in dry dichloromethane (1 ml) isopropylamine (17.2 µl, 0.2 mmol) was added at −20°C. The mixture turned red immediately. The solution was concentrated under reduced pressure to ca. 0.1 ml volume and pentane (0.5 ml) was added with stirring. Crystallization at −80°C for 3 days afforded isopropylamine adduct **3** (26 mg, 72%) as red crystals, which were collected after removal of mother liquor at −20°C and drying in vacuo. These crystals melt immediately upon warming up. Crystal suitable for X-ray crystallography was grown by *in situ* cooling of the compound in cold perfluoroethyl oil. **^{1}H NMR** (400 MHz, CD_3CN) δ 5.53 (s, 1H, =CH), 1.99 (s, 6H, 2Me); **^{13}C NMR** (101 MHz, CD_3CN) δ 189.7 (C=O), 100.6 (=CH), 27.2 (Me); **^{19}F NMR** (376 MHz, CD_3CN) δ −32.4 (s, 6F); signals of isopropylamine are invisible in ^{1}H and ^{13}C NMR due to fast Berry pseudorotation causing broadening. **^{1}H NMR** (400 MHz, benzene-d$_6$) δ 4.86 (s, 1H, =CH), 3.2 (br s, 1H, N-CH), 2.2 (br s, 6H, 2Me(amine)), 1.59 (s, 6H, 2Me(acac)); **^{19}F NMR** (376 MHz, benzene-d$_6$) δ −29.9 (s, 6F); **IR** (ATR): $\tilde{\nu}$ = 3151, 2985, 1561, 1520, 1363, 1102, 1027, 1027, 970, 954, 807, 723, 631 cm^{-1}; Elem. Anal. calcd. for $CuC_{10}H_{16}O_2F_6N$: 32.61% C, 3.89% N, 4.48% H, found: 33.38% C, 4.02% N, 3.80% H. **CCDC** 2546506.

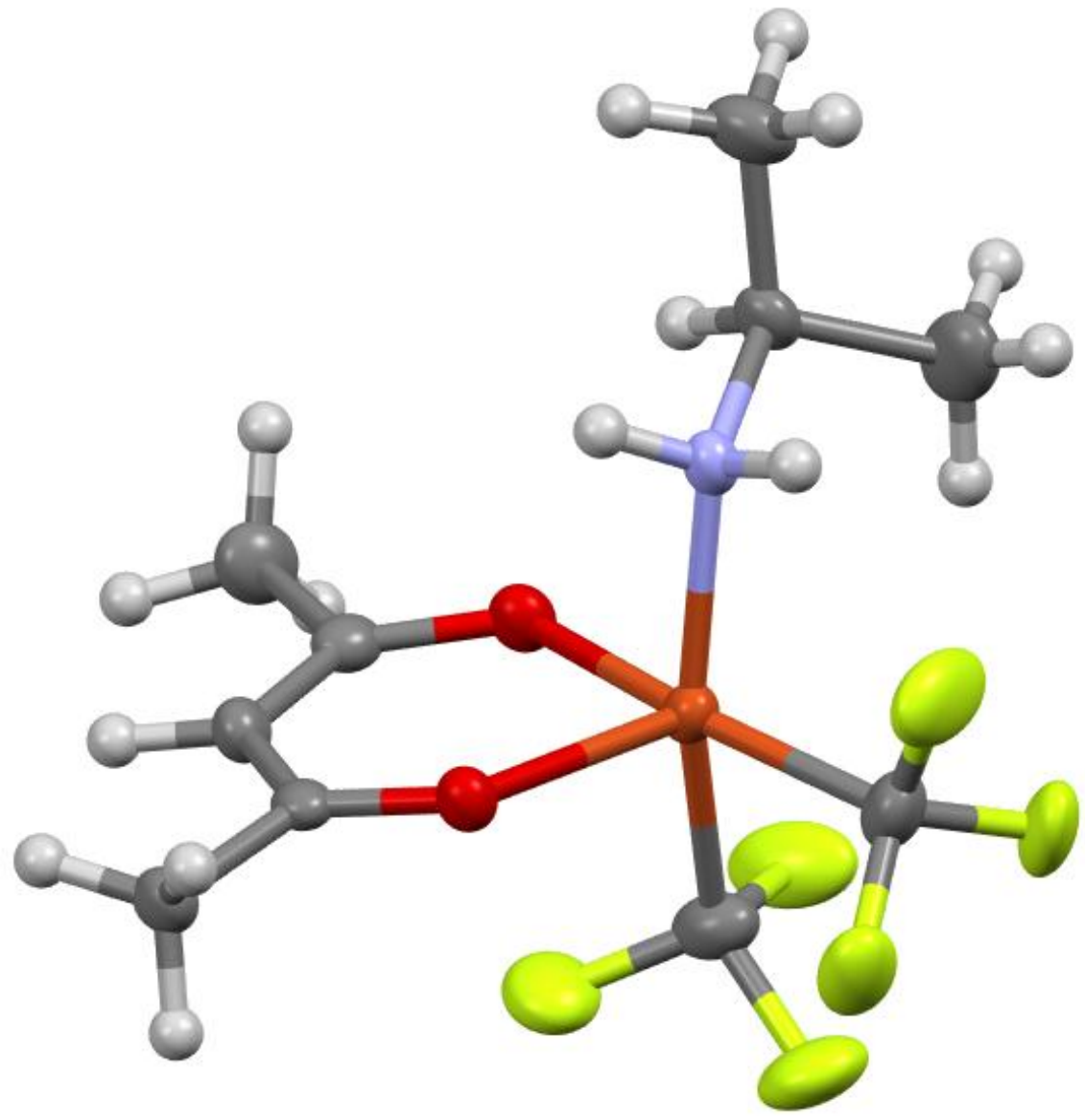

*Figure S3. Crystal structure of isopropylamine adduct [(acac)Cu(CF_3)$_2$(iPrNH$_2$)] **3***

Table 7. Crystal data and structure refinement for 3

CCDC number	2546506
Empirical formula	$C_{10}H_{16}CuF_6NO_2$
Formula weight	359.78
Temperature [K]	150.00
Crystal system	monoclinic
Space group (number)	$C2/c$ (15)
a [Å]	23.1196(15)
b [Å]	9.2773(7)
c [Å]	15.2468(9)
α [°]	90
β [°]	117.317(2)
γ [°]	90
Volume [Å^3]	2905.6(3)
Z	8
ρ_{calc} [gcm^{-3}]	1.645
μ [mm^{-1}]	1.569
F(000)	1456
Crystal size [mm^3]	0.304×0.411×0.513
Crystal colour	red
Crystal shape	block
Radiation	MoK_α (λ=0.71073 Å)
2θ range [°]	3.97 to 50.77 (0.83 Å)
Index ranges	$-27 \leq h \leq 27$<br>$-11 \leq k \leq 11$<br>$-16 \leq l \leq 18$
Reflections collected	17809
Independent reflections	2663<br>R_{int} = 0.0597<br>R_{sigma} = 0.0378

Completeness to θ = 25.242°	100.0 %
Data / Restraints / Parameters	2663 / 0 / 185
Absorption correction T_{min}/T_{max} (method)	0.4541 / 0.7452 (multi-scan)
Goodness-of-fit on F^2	1.053
Final *R* indexes [$I \geq 2\sigma(I)$]	$R_1 = 0.0283$ $wR_2 = 0.0686$
Final *R* indexes [all data]	$R_1 = 0.0349$ $wR_2 = 0.0717$
Largest peak/hole [eÅ^{-3}]	0.39/−0.27

Table 8. Bond lengths and angles for 3

Atom–Atom	Length [Å]
Cu1–O1	2.1868(15)
Cu1–O2	1.8796(15)
Cu1–N1	1.9864(18)
Cu1–C2	1.957(2)
Cu1–C1	1.931(3)
O1–C4	1.264(3)
O2–C6	1.290(3)
F2–C1	1.339(3)
F3–C1	1.334(3)
F1–C1	1.359(3)
F4–C2	1.338(3)
F5–C2	1.331(3)
F6–C2	1.329(3)
N1–H1A	0.9100
N1–H1B	0.9100
N1–C8	1.497(3)
C4–C3	1.503(3)
C4–C5	1.404(3)
C6–C5	1.383(3)
C6–C7	1.503(3)
C8–H8	1.0000
C8–C9	1.512(3)
C8–C10	1.513(3)
C3–H3A	0.9800
C3–H3B	0.9800
C3–H3C	0.9800
C5–H5	0.9500
C7–H7A	0.9800
C7–H7B	0.9800
C7–H7C	0.9800
C9–H9A	0.9800
C9–H9B	0.9800
C9–H9C	0.9800
C10–H10A	0.9800
C10–H10B	0.9800
C10–H10C	0.9800
**Atom–Atom–Atom**	**Angle [°]**
O2–Cu1–O1	91.25(6)
O2–Cu1–N1	88.33(7)
O2–Cu1–C2	87.38(9)
O2–Cu1–C1	169.72(9)
N1–Cu1–O1	94.96(7)
C2–Cu1–O1	101.87(9)
C2–Cu1–N1	162.71(9)
C1–Cu1–O1	98.51(9)
C1–Cu1–N1	94.00(10)
C1–Cu1–C2	87.46(11)
C4–O1–Cu1	121.12(13)
C6–O2–Cu1	127.08(14)
Cu1–N1–H1A	108.6
Cu1–N1–H1B	108.6
H1A–N1–H1B	107.6
C8–N1–Cu1	114.76(14)
C8–N1–H1A	108.6
C8–N1–H1B	108.6
O1–C4–C3	116.7(2)
O1–C4–C5	124.6(2)
C5–C4–C3	118.7(2)
O2–C6–C5	127.0(2)
O2–C6–C7	113.1(2)
C5–C6–C7	119.8(2)
N1–C8–H8	108.3
N1–C8–C9	110.46(19)
N1–C8–C10	109.02(19)
C9–C8–H8	108.3
C9–C8–C10	112.3(2)
C10–C8–H8	108.3
C4–C3–H3A	109.5
C4–C3–H3B	109.5
C4–C3–H3C	109.5
H3A–C3–H3B	109.5
H3A–C3–H3C	109.5
H3B–C3–H3C	109.5
C4–C5–H5	116.4
C6–C5–C4	127.2(2)
C6–C5–H5	116.4
F4–C2–Cu1	121.07(19)
F5–C2–Cu1	110.27(16)
F5–C2–F4	104.3(2)
F6–C2–Cu1	106.31(16)
F6–C2–F4	106.4(2)
F6–C2–F5	107.9(2)
F2–C1–Cu1	113.48(17)
F2–C1–F1	103.8(2)
F3–C1–Cu1	114.35(18)
F3–C1–F2	107.68(19)
F3–C1–F1	105.0(2)
F1–C1–Cu1	111.64(16)
C6–C7–H7A	109.5
C6–C7–H7B	109.5
C6–C7–H7C	109.5
H7A–C7–H7B	109.5
H7A–C7–H7C	109.5
H7B–C7–H7C	109.5
C8–C9–H9A	109.5
C8–C9–H9B	109.5
C8–C9–H9C	109.5
H9A–C9–H9B	109.5
H9A–C9–H9C	109.5
H9B–C9–H9C	109.5
C8–C10–H10A	109.5
C8–C10–H10B	109.5
C8–C10–H10C	109.5
H10A–C10–H10B	109.5
H10A–C10–H10C	109.5
H10B–C10–H10C	109.5

Table 9. Torsion angles for 3

**Atom–Atom–Atom–Atom**	**Torsion Angle [°]**
Cu1–O1–C4–C3	168.18(14)
Cu1–O1–C4–C5	−11.1(3)
Cu1–O2–C6–C5	6.8(3)
Cu1–O2–C6–C7	−174.32(15)
Cu1–N1–C8–C9	171.14(16)
Cu1–N1–C8–C10	−65.1(2)
O1–Cu1–O2–C6	−11.80(17)
O1–C4–C5–C6	1.6(4)
O2–C6–C5–C4	2.0(4)
N1–Cu1–O2–C6	−106.72(18)
C3–C4–C5–C6	−177.7(2)
C2–Cu1–O2–C6	90.03(18)
C1–Cu1–O2–C6	150.0(5)
C7–C6–C5–C4	−176.8(2)

Synthesis of triethylphosphine oxide adduct 4.

To the solid (acac)Cu(CF_3)$_2$ **1** (60 mg, 0.2 mmol) a solution of triethylphosphine oxide (27 mg, 0.2 mmol) in dry dichloromethane (0.2 ml) was added at room temperature. To the resulting red solution pentane (1.8 ml) was added upon stirring. Crystallization in the freezer at −80°C for 3 days afforded TEPO-adduct **4** (49 mg, 56%) as orange crystals (suitable for X-ray), which were collected after removal of mother liquor at −20°C and drying in vacuo. **^{1}H NMR** (400 MHz, MeCN-d$_3$) δ 5.63 (s, 1H, =CH), 2.02 (s, 3H, Me(acac)), 2.01 (s, 3H, Me(acac)), 1.68-1.58 (m, 6H, CH_2), 1.10-1.03 (m, 9H, CH_3); **^{1}H NMR** (400 MHz, $CDCl_3$) δ 5.63 (s, 1H, =CH), 2.10 (s, 6H, Me(acac)), 1.72 (dq, J = 11.5, 7.6 Hz, 6H, CH_2), 1.17 (dt, J = 15.6, 7.6 Hz, 9H, CH_3); **^{19}F NMR** (376 MHz, MeCN-d$_3$) δ −33.1 (br s, 6F); **^{31}P NMR** (162 MHz, MeCN-d$_3$) δ 53.0 (br s); **IR** (ATR): $\tilde{\nu}$ = 2977, 2947, 2877, 1593, 1523, 1382, 1143, 1125, 1081, 984, 785, 723, 614 cm^{-1}; Elem. Anal. calcd. for $C_{13}H_{22}O_3CuF_6P$: 35.91% C, 5.10% H, found: 36.17% C, 5.19% H. **CCDC** 2546511.

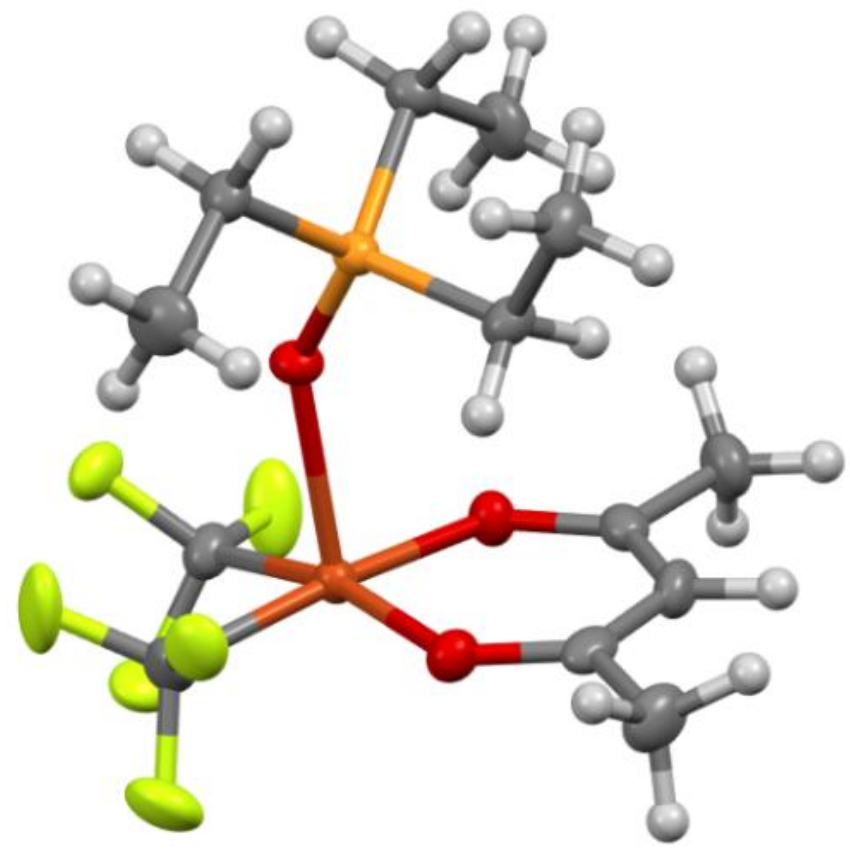

Figure S4. Crystal structure of [(acac)Cu(CF$_3$)$_2$(TEPO)] ***4***

Table 10. Crystal data and structure refinement for 4

CCDC number	2546511
Empirical formula	$C_{13}H_{22}CuF_6O_3P$
Formula weight	434.81
Temperature [K]	150.00
Crystal system	monoclinic
Space group (number)	$P2_1/c$ (14)
a [Å]	13.4593(8)
b [Å]	8.4946(4)
c [Å]	16.1462(10)
α [°]	90
β [°]	101.342(2)
γ [°]	90
Volume [Å^3]	1809.97(18)
Z	4
ρ_{calc} [gcm^{-3}]	1.596
μ [mm^{-1}]	1.361
F(000)	888
Crystal size [mm^3]	0.166×0.25×0.522
Crystal colour	orange
Crystal shape	block
Radiation	MoK_α (λ=0.71073 Å)
2θ range [°]	5.15 to 50.73 (0.83 Å)
Index ranges	−16 ≤ h ≤ 16<br>−10 ≤ k ≤ 10<br>−19 ≤ l ≤ 19
Reflections collected	35445
Independent reflections	3318<br>R_{int} = 0.0838<br>R_{sigma} = 0.0369
Completeness to θ = 25.242°	100.0 %
Data / Restraints / Parameters	3318 / 6 / 276
Absorption correction T_{min}/T_{max} (method)	0.5447 / 0.7452 (multi-scan)
Goodness-of-fit on F^2	1.035
Final R indexes [I≥2σ(I)]	R_1 = 0.0280<br>wR_2 = 0.0685
Final R indexes [all data]	R_1 = 0.0382<br>wR_2 = 0.0740
Largest peak/hole [eÅ^{-3}]	0.39/−0.32

Table 11. Bond lengths and angles for 4

Atom–Atom	Length [Å]
Cu1–O3	2.2544(15)
Cu1–O1	1.8932(15)
Cu1–O2	1.8918(15)
Cu1–C2	1.948(2)
Cu1–C1	1.945(2)
P1–O3	1.4980(15)
P1–C8	1.797(2)
P1–C10	1.807(2)
P1–C12	1.800(2)
F5A–C2	1.317(9)
O1–C4	1.275(3)
O2–C6	1.276(3)
F1B–C1	1.324(10)
F4A–C2	1.264(6)
F2B–C1	1.431(5)
C4–C5	1.388(3)
C4–C3	1.498(3)
C5–H5	0.9500
C5–C6	1.390(3)
C6–C7	1.500(3)
C8–H8A	0.9900
C8–H8B	0.9900
C8–C9	1.527(3)
F6A–C2	1.412(4)
C2–F5B	1.321(10)
C2–F6B	1.311(5)
C2–F4B	1.417(6)
C10–H10A	0.9900
C10–H10B	0.9900
C10–C11	1.528(3)
C12–H12A	0.9900
C12–H12B	0.9900
C12–C13	1.532(3)
C1–F3B	1.308(4)
C1–F2A	1.250(5)
C1–F3A	1.429(4)
C1–F1A	1.304(10)
C11–H11A	0.9800
C11–H11B	0.9800
C11–H11C	0.9800
C13–H13A	0.9800
C13–H13B	0.9800
C13–H13C	0.9800
C3–H3A	0.9800
C3–H3B	0.9800
C3–H3C	0.9800
C7–H7A	0.9800
C7–H7B	0.9800
C7–H7C	0.9800
C9–H9A	0.9800
C9–H9B	0.9800
C9–H9C	0.9800
**Atom–Atom–Atom**	**Angle [°]**
O1–Cu1–O3	97.41(6)
O1–Cu1–C2	166.35(9)
O1–Cu1–C1	88.40(9)
O2–Cu1–O3	94.33(6)
O2–Cu1–O1	94.28(7)
O2–Cu1–C2	89.40(9)
O2–Cu1–C1	172.06(9)
C2–Cu1–O3	95.41(8)
C1–Cu1–O3	92.74(9)
C1–Cu1–C2	86.33(11)
O3–P1–C8	112.16(10)
O3–P1–C10	112.18(10)
O3–P1–C12	111.66(10)
C8–P1–C10	106.92(11)
C8–P1–C12	105.92(11)
C12–P1–C10	107.64(11)
P1–O3–Cu1	143.99(9)
C4–O1–Cu1	125.65(15)
C6–O2–Cu1	125.48(15)
O1–C4–C5	125.2(2)
O1–C4–C3	114.6(2)
C5–C4–C3	120.2(2)
C4–C5–H5	118.0
C4–C5–C6	124.0(2)
C6–C5–H5	118.0
O2–C6–C5	125.3(2)
O2–C6–C7	114.7(2)
C5–C6–C7	120.0(2)
P1–C8–H8A	109.0
P1–C8–H8B	109.0
H8A–C8–H8B	107.8
C9–C8–P1	113.02(18)
C9–C8–H8A	109.0
C9–C8–H8B	109.0
F5A–C2–Cu1	113.5(7)
F5A–C2–F6A	102.2(6)
F4A–C2–Cu1	113.4(3)
F4A–C2–F5A	112.2(8)
F4A–C2–F6A	106.5(3)
F6A–C2–Cu1	108.0(2)
F5B–C2–Cu1	122.9(7)
F5B–C2–F4B	99.1(8)
F6B–C2–Cu1	113.3(2)
F6B–C2–F5B	109.0(6)
F6B–C2–F4B	103.0(3)
F4B–C2–Cu1	106.6(3)
P1–C10–H10A	108.4
P1–C10–H10B	108.4
H10A–C10–H10B	107.4
C11–C10–P1	115.70(17)
C11–C10–H10A	108.4
C11–C10–H10B	108.4
P1–C12–H12A	109.1
P1–C12–H12B	109.1
H12A–C12–H12B	107.9
C13–C12–P1	112.36(17)
C13–C12–H12A	109.1

C13–C12–H12B	109.1
F1B–C1–Cu1	122.8(8)
F1B–C1–F2B	99.2(9)
F2B–C1–Cu1	105.8(2)
F3B–C1–Cu1	113.2(2)
F3B–C1–F1B	110.5(6)
F3B–C1–F2B	102.1(3)
F2A–C1–Cu1	113.7(3)
F2A–C1–F3A	105.1(4)
F2A–C1–F1A	112.4(10)
F3A–C1–Cu1	108.9(2)
F1A–C1–Cu1	113.7(7)
F1A–C1–F3A	101.8(5)
C10–C11–H11A	109.5
C10–C11–H11B	109.5
C10–C11–H11C	109.5
H11A–C11–H11B	109.5
H11A–C11–H11C	109.5
H11B–C11–H11C	109.5
C12–C13–H13A	109.5
C12–C13–H13B	109.5
C12–C13–H13C	109.5
H13A–C13–H13B	109.5

H13A–C13–H13C	109.5
H13B–C13–H13C	109.5
C4–C3–H3A	109.5
C4–C3–H3B	109.5
C4–C3–H3C	109.5
H3A–C3–H3B	109.5
H3A–C3–H3C	109.5
H3B–C3–H3C	109.5
C6–C7–H7A	109.5
C6–C7–H7B	109.5
C6–C7–H7C	109.5
H7A–C7–H7B	109.5
H7A–C7–H7C	109.5
H7B–C7–H7C	109.5
C8–C9–H9A	109.5
C8–C9–H9B	109.5
C8–C9–H9C	109.5
H9A–C9–H9B	109.5
H9A–C9–H9C	109.5
H9B–C9–H9C	109.5

Table 12. Torsion angles for 4

Atom–Atom–Atom–Atom	Torsion Angle [°]
Cu1–O1–C4–C5	−0.7(3)
Cu1–O1–C4–C3	179.86(15)
Cu1–O2–C6–C5	2.3(3)
Cu1–O2–C6–C7	−178.00(15)
O3–Cu1–O1–C4	−92.52(18)
O3–Cu1–O2–C6	94.58(18)
O3–P1–C8–C9	56.4(2)
O3–P1–C10–C11	−172.00(17)
O3–P1–C12–C13	−47.1(2)
O1–Cu1–O2–C6	−3.20(19)
O1–C4–C5–C6	−1.3(4)
O2–Cu1–O1–C4	2.42(18)
C4–C5–C6–O2	0.4(4)
C4–C5–C6–C7	−179.2(2)
C8–P1–O3–Cu1	−129.02(16)
C8–P1–C10–C11	−48.7(2)
C8–P1–C12–C13	−169.46(17)
C2–Cu1–O1–C4	107.7(4)
C2–Cu1–O2–C6	−170.04(19)
C10–P1–O3–Cu1	−8.7(2)
C10–P1–C8–C9	−66.9(2)
C10–P1–C12–C13	76.45(19)
C12–P1–O3–Cu1	112.27(16)
C12–P1–C8–C9	178.49(18)
C12–P1–C10–C11	64.8(2)
C1–Cu1–O1–C4	174.93(19)
C3–C4–C5–C6	178.1(2)

Synthesis of chloride anionic complex 5.

To the solid (acac)Cu(CF_3)$_2$ **1** (60 mg, 0.2 mmol) in a 10 ml Schlenk tube a pre-cooled solution of tetrabutylammonium chloride (56 mg, 0.2 mmol) in dry dichloromethane (0.3 ml) was added at −20°C via syringe. The resulting red solution was stirred for 5 minutes at −20°C. Then the stirring was stopped, and the mixture was layered with 3 ml of pentane. Crystallization in the freezer at −80°C for 3 days afforded anionic chlorocuprate(III) complex **5** (94 mg, 81%) as red crystals (suitable for X-ray), which were collected after removal of mother liquor at −20°C and drying in vacuo. **^{1}H NMR** (400 MHz, $CDCl_3$) δ 5.60 (s, 1H, =CH), 3.33 (br s, 8H, CH_2), 2.07 (s, 6H, Me), 1.65 (br s, 8H, CH_2), 1.43 (q, J = 7.2 Hz, 8H, CH_2), 0.98 (t, J = 7.2 Hz, 12H, CH_3); **^{19}F NMR** (376 MHz, MeCN-d_3) δ −30.2 (s, 6F); **IR** (ATR): $\tilde{\nu}$ = 2966, 2936, 2877, 1598, 1520, 1388, 1143, 1127, 1103, 1068, 959, 782, 720, 601 cm^{-1}; Elem. Anal. calcd. for $C_{23}H_{43}O_2CuF_6ClN$: 47.74% C, 2.42% N, 7.49% H, found: 47.91% C, 3.02% N, 7.78% H. **CCDC** 2546512.

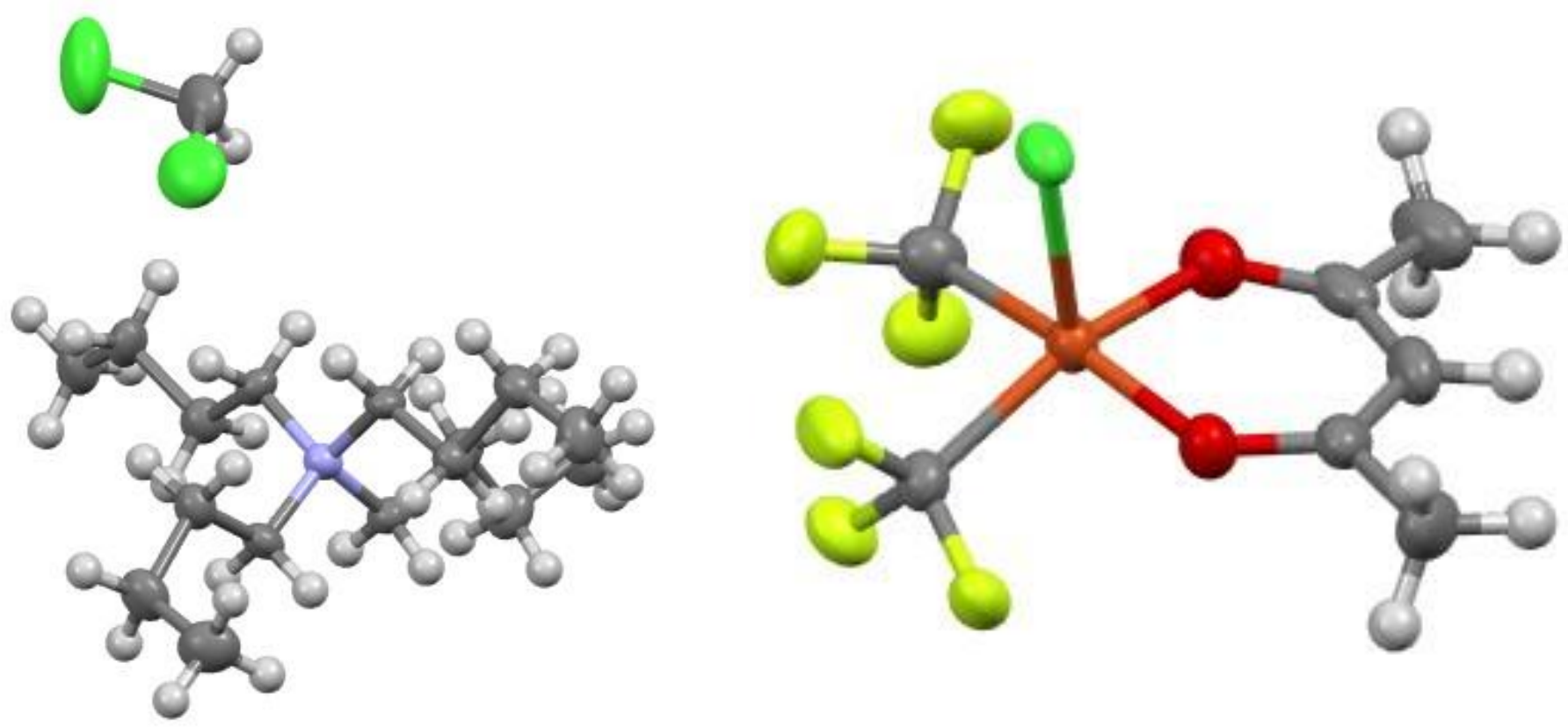

Figure S5. Crystal structure of Bu_4N^+[(acac)Cu(CF_3)$_2$(Cl)]$^-$ **5** *crystallized as a DCM solvate*

Table 13. Crystal data and structure refinement for 5

CCDC number	2546512
Empirical formula	$C_{24}H_{45}Cl_3CuF_6NO_2$
Formula weight	663.50
Temperature [K]	150.00
Crystal system	orthorhombic
Space group (number)	$Pna2_1$ (33)
a [Å]	19.2413(12)
b [Å]	11.6909(8)
c [Å]	14.2142(9)
α [°]	90
β [°]	90
γ [°]	90
Volume [Å^3]	3197.5(4)
Z	4
ρ_{calc} [gcm^{-3}]	1.378
μ [mm^{-1}]	0.990
F(000)	1384
Crystal size [mm^3]	0.138×0.285×0.362
Crystal colour	red
Crystal shape	block
Radiation	MoK_α (λ=0.71073 Å)
2θ range [°]	4.08 to 50.72 (0.83 Å)
Index ranges	$-23 \leq h \leq 23$<br>$-14 \leq k \leq 14$<br>$-17 \leq l \leq 17$
Reflections collected	90256
Independent reflections	5845<br>R_{int} = 0.0929<br>R_{sigma} = 0.0380
Completeness to θ = 25.242°	100.0 %
Data / Restraints / Parameters	5845 / 1 / 341
Absorption correction T_{min}/T_{max} (method)	0.5445 / 0.7452 (multi-scan)
Goodness-of-fit on F^2	1.255
Final R indexes [$I \geq 2\sigma(I)$]	R_1 = 0.0338<br>wR_2 = 0.0770
Final R indexes [all data]	R_1 = 0.0519<br>wR_2 = 0.0954
Largest peak/hole [eÅ^{-3}]	0.42/−0.40
Flack X parameter	0.04(2)

Table 14. Bond lengths and angles for 5

Atom–Atom	Length [Å]
Cu1–Cl1	2.5005(12)
Cu1–O1	1.902(4)
Cu1–O2	1.916(4)
Cu1–C2	1.947(6)
Cu1–C1	1.933(6)
Cl3–C8	1.766(10)
Cl2–C8	1.756(9)
F5–C2	1.340(7)
F4–C2	1.331(6)
F6–C2	1.363(6)
F2–C1	1.342(7)
F3–C1	1.336(7)
O1–C6	1.271(7)
F1–C1	1.341(8)
O2–C4	1.266(7)
N1–C21	1.518(6)
N1–C17	1.518(6)
N1–C13	1.523(6)
N1–C9	1.515(6)
C21–H21A	0.9900
C21–H21B	0.9900
C21–C22	1.518(7)
C17–H17A	0.9900
C17–H17B	0.9900
C17–C18	1.515(8)
C13–H13A	0.9900
C13–H13B	0.9900
C13–C14	1.511(8)
C4–C5	1.392(9)
C4–C3	1.508(8)
C23–H23A	0.9900
C23–H23B	0.9900
C23–C22	1.515(7)
C23–C24	1.514(9)
C9–H9A	0.9900
C9–H9B	0.9900
C9–C10	1.520(7)
C18–H18A	0.9900
C18–H18B	0.9900
C18–C19	1.536(8)
C15–H15A	0.9900
C15–H15B	0.9900
C15–C16	1.529(9)
C15–C14	1.525(9)
C22–H22A	0.9900
C22–H22B	0.9900
C16–H16A	0.9800
C16–H16B	0.9800
C16–H16C	0.9800
C6–C5	1.384(8)
C6–C7	1.507(8)
C10–H10A	0.9900
C10–H10B	0.9900
C10–C11	1.516(8)
C14–H14A	0.9900
C14–H14B	0.9900
C5–H5	0.9500
C24–H24A	0.9800
C24–H24B	0.9800
C24–H24C	0.9800
C20–H20A	0.9800
C20–H20B	0.9800
C20–H20C	0.9800
C20–C19	1.496(9)
C11–H11A	0.9900
C11–H11B	0.9900
C11–C12	1.510(10)
C19–H19A	0.9900
C19–H19B	0.9900
C3–H3A	0.9800
C3–H3B	0.9800
C3–H3C	0.9800
C7–H7A	0.9800
C7–H7B	0.9800
C7–H7C	0.9800
C8–H8A	0.9900
C8–H8B	0.9900
C12–H12A	0.9800
C12–H12B	0.9800
C12–H12C	0.9800
**Atom–Atom–Atom**	**Angle [°]**
O1–Cu1–Cl1	92.21(13)
O1–Cu1–O2	94.51(18)
O1–Cu1–C2	90.84(19)
O1–Cu1–C1	174.2(2)
O2–Cu1–Cl1	102.89(13)
O2–Cu1–C2	153.6(2)
O2–Cu1–C1	86.0(2)
C2–Cu1–Cl1	102.66(17)
C1–Cu1–Cl1	93.3(2)
C1–Cu1–C2	86.2(3)
C6–O1–Cu1	124.5(4)
C4–O2–Cu1	124.8(4)
C21–N1–C13	105.6(4)
C17–N1–C21	111.1(4)
C17–N1–C13	111.6(4)
C9–N1–C21	111.1(4)
C9–N1–C17	106.4(4)
C9–N1–C13	111.2(4)
F5–C2–Cu1	111.0(4)
F5–C2–F6	103.8(4)
F4–C2–Cu1	114.2(4)
F4–C2–F5	105.5(5)

F4–C2–F6	105.3(4)
F6–C2–Cu1	116.1(4)
N1–C21–H21A	108.0
N1–C21–H21B	108.0
H21A–C21–H21B	107.2
C22–C21–N1	117.2(4)
C22–C21–H21A	108.0
C22–C21–H21B	108.0
N1–C17–H17A	108.1
N1–C17–H17B	108.1
H17A–C17–H17B	107.3
C18–C17–N1	116.6(4)
C18–C17–H17A	108.1
C18–C17–H17B	108.1
N1–C13–H13A	108.0
N1–C13–H13B	108.0
H13A–C13–H13B	107.2
C14–C13–N1	117.2(4)
C14–C13–H13A	108.0
C14–C13–H13B	108.0
O2–C4–C5	125.3(5)
O2–C4–C3	115.5(6)
C5–C4–C3	119.3(6)
H23A–C23–H23B	107.7
C22–C23–H23A	108.9
C22–C23–H23B	108.9
C24–C23–H23A	108.9
C24–C23–H23B	108.9
C24–C23–C22	113.3(5)
N1–C9–H9A	108.4
N1–C9–H9B	108.4
N1–C9–C10	115.7(4)
H9A–C9–H9B	107.4
C10–C9–H9A	108.4
C10–C9–H9B	108.4
F2–C1–Cu1	110.0(4)
F3–C1–Cu1	108.5(4)
F3–C1–F2	107.0(5)
F3–C1–F1	105.6(5)
F1–C1–Cu1	117.8(4)
F1–C1–F2	107.4(5)
C17–C18–H18A	109.5
C17–C18–H18B	109.5
C17–C18–C19	110.9(5)
H18A–C18–H18B	108.1
C19–C18–H18A	109.5
C19–C18–H18B	109.5
H15A–C15–H15B	107.9
C16–C15–H15A	109.1
C16–C15–H15B	109.1
C14–C15–H15A	109.1
C14–C15–H15B	109.1
C14–C15–C16	112.4(5)

C21–C22–H22A	110.0
C21–C22–H22B	110.0
C23–C22–C21	108.5(4)
C23–C22–H22A	110.0
C23–C22–H22B	110.0
H22A–C22–H22B	108.4
C15–C16–H16A	109.5
C15–C16–H16B	109.5
C15–C16–H16C	109.5
H16A–C16–H16B	109.5
H16A–C16–H16C	109.5
H16B–C16–H16C	109.5
O1–C6–C5	126.2(5)
O1–C6–C7	114.0(5)
C5–C6–C7	119.8(6)
C9–C10–H10A	109.9
C9–C10–H10B	109.9
H10A–C10–H10B	108.3
C11–C10–C9	109.1(5)
C11–C10–H10A	109.9
C11–C10–H10B	109.9
C13–C14–C15	109.4(4)
C13–C14–H14A	109.8
C13–C14–H14B	109.8
C15–C14–H14A	109.8
C15–C14–H14B	109.8
H14A–C14–H14B	108.2
C4–C5–H5	117.9
C6–C5–C4	124.2(5)
C6–C5–H5	117.9
C23–C24–H24A	109.5
C23–C24–H24B	109.5
C23–C24–H24C	109.5
H24A–C24–H24B	109.5
H24A–C24–H24C	109.5
H24B–C24–H24C	109.5
H20A–C20–H20B	109.5
H20A–C20–H20C	109.5
H20B–C20–H20C	109.5
C19–C20–H20A	109.5
C19–C20–H20B	109.5
C19–C20–H20C	109.5
C10–C11–H11A	109.2
C10–C11–H11B	109.2
H11A–C11–H11B	107.9
C12–C11–C10	111.9(6)
C12–C11–H11A	109.2
C12–C11–H11B	109.2
C18–C19–H19A	108.7
C18–C19–H19B	108.7
C20–C19–C18	114.2(5)
C20–C19–H19A	108.7
C20–C19–H19B	108.7

H19A–C19–H19B	107.6
C4–C3–H3A	109.5
C4–C3–H3B	109.5
C4–C3–H3C	109.5
H3A–C3–H3B	109.5
H3A–C3–H3C	109.5
H3B–C3–H3C	109.5
C6–C7–H7A	109.5
C6–C7–H7B	109.5
C6–C7–H7C	109.5
H7A–C7–H7B	109.5
H7A–C7–H7C	109.5
H7B–C7–H7C	109.5
Cl3–C8–H8A	109.2
Cl3–C8–H8B	109.2
Cl2–C8–Cl3	111.9(3)
Cl2–C8–H8A	109.2
Cl2–C8–H8B	109.2
H8A–C8–H8B	107.9
C11–C12–H12A	109.5
C11–C12–H12B	109.5
C11–C12–H12C	109.5
H12A–C12–H12B	109.5
H12A–C12–H12C	109.5
H12B–C12–H12C	109.5

Table 15. Torsion angles for 5

**Atom–Atom–Atom–Atom**	**Torsion Angle [°]**
Cu1–O1–C6–C5	−4.9(8)
Cu1–O1–C6–C7	176.3(4)
Cu1–O2–C4–C5	−8.2(8)
Cu1–O2–C4–C3	170.7(4)
O1–C6–C5–C4	0.8(10)
O2–C4–C5–C6	6.5(9)
N1–C21–C22–C23	172.6(5)
N1–C17–C18–C19	−176.2(5)
N1–C13–C14–C15	170.6(4)
N1–C9–C10–C11	−171.4(5)
C21–N1–C17–C18	56.0(6)
C21–N1–C13–C14	166.9(4)
C21–N1–C9–C10	−54.1(6)
C17–N1–C21–C22	53.5(6)
C17–N1–C13–C14	−72.3(6)
C17–N1–C9–C10	−175.2(5)
C17–C18–C19–C20	79.6(7)
C13–N1–C21–C22	174.7(5)
C13–N1–C17–C18	−61.5(6)
C13–N1–C9–C10	63.2(6)
C9–N1–C21–C22	−64.7(6)
C9–N1–C17–C18	177.0(5)
C9–N1–C13–C14	46.3(6)
C9–C10–C11–C12	179.3(6)
C16–C15–C14–C13	173.1(5)
C24–C23–C22–C21	174.0(5)
C3–C4–C5–C6	−172.4(6)
C7–C6–C5–C4	179.5(6)

Synthesis of solvates 1a and 1b

[(Acac)Cu(CF$_3$)$_2$(MeCN)] 1a: Complex **1** (20 mg) was dissolved in 5:1 DCM/MeCN (0.5 ml) and the mixture was cooled down to 0°C (ice bath), then layered with 2.5 ml of dry pentane. Crystallization of this mixture in the freezer at −80°C for 3 days afforded orange crystals of the solvate **1a**, which were collected at −80°C and put directly into pre-cooled perfluorohexane oil to record the X-ray crystal structure. CCDC 2546502.

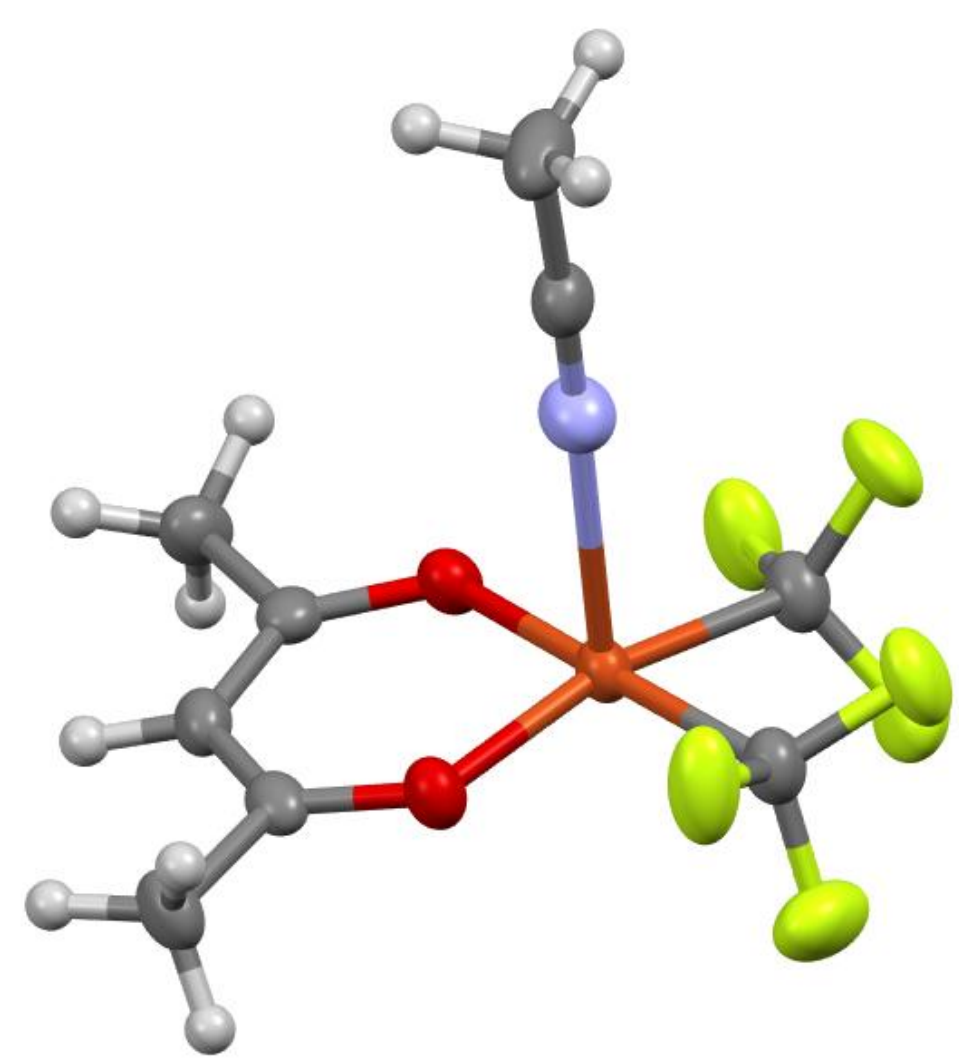

Figure S6. Crystal structure of [(acac)Cu(CF$_3$)$_2$(MeCN)] ***1a***

Table 16. Crystal data and structure refinement for 1a

CCDC number	2546502
Empirical formula	$C_9H_{10}CuF_6NO_2$
Formula weight	341.72
Temperature [K]	150.00
Crystal system	orthorhombic
Space group (number)	*Pbca* (61)
a [Å]	13.0189(13)
b [Å]	8.7770(8)
c [Å]	22.512(2)
α [°]	90
β [°]	90
γ [°]	90
Volume [Å^3]	2572.4(4)
Z	8
ρ_{calc} [gcm^{-3}]	1.765
μ [mm^{-1}]	1.767
F(000)	1360
Crystal size [mm^3]	0.124×0.202×0.704
Crystal colour	clear yellow
Crystal shape	block
Radiation	MoK_α (λ=0.71073 Å)
2θ range [°]	4.78 to 50.70 (0.83 Å)
Index ranges	−15 ≤ h ≤ 15<br>−10 ≤ k ≤ 10<br>−27 ≤ l ≤ 27
Reflections collected	124396
Independent reflections	2360<br>R_{int} = 0.0726<br>R_{sigma} = 0.0159
Completeness to θ = 25.242°	100.0 %
Data / Restraints / Parameters	2360 / 0 / 175
Absorption correction T_{min}/T_{max} (method)	0.5389 / 0.7452 (multi-scan)
Goodness-of-fit on F^2	1.191
Final *R* indexes [$I \geq 2\sigma(I)$]	R_1 = 0.0265<br>wR_2 = 0.0626
Final *R* indexes [all data]	R_1 = 0.0344<br>wR_2 = 0.0741
Largest peak/hole [eÅ^{-3}]	0.31/−0.40

Table 17. Bond lengths and angles for 1a

Atom–Atom	Length [Å]
Cu1–O2	1.8917(16)
Cu1–O1	1.8836(16)
Cu1–N1	2.422(2)
Cu1–C2	1.957(2)
Cu1–C1	1.958(2)
F5–C2	1.329(3)
F1–C1	1.340(3)
F2–C1	1.327(3)
F3–C1	1.343(3)
O2–C6	1.283(3)
O1–C4	1.286(3)
F6–C2	1.330(3)
F4–C2	1.331(3)
N1–C8	1.131(3)
C6–C5	1.388(3)
C6–C7	1.495(3)
C4–C5	1.385(3)
C4–C3	1.498(3)
C5–H5	0.9500
C8–C9	1.452(4)
C7–H7A	0.9800
C7–H7B	0.9800
C7–H7C	0.9800
C3–H3A	0.9800
C3–H3B	0.9800
C3–H3C	0.9800
C9–H9A	0.9800
C9–H9B	0.9800
C9–H9C	0.9800
**Atom–Atom–Atom**	**Angle [°]**
O2–Cu1–N1	94.61(7)
O2–Cu1–C2	88.11(8)
O2–Cu1–C1	167.59(9)
O1–Cu1–O2	94.75(7)
O1–Cu1–N1	91.08(7)
O1–Cu1–C2	175.88(9)
O1–Cu1–C1	90.44(9)
C2–Cu1–N1	91.65(9)
C2–Cu1–C1	86.18(10)
C1–Cu1–N1	96.56(9)
C6–O2–Cu1	125.32(15)
C4–O1–Cu1	125.33(15)
C8–N1–Cu1	176.6(2)
O2–C6–C5	125.0(2)
O2–C6–C7	114.8(2)
C5–C6–C7	120.3(2)
O1–C4–C5	125.1(2)
O1–C4–C3	115.0(2)
C5–C4–C3	119.9(2)
C6–C5–H5	117.8
C4–C5–C6	124.4(2)
C4–C5–H5	117.8
N1–C8–C9	178.3(3)
F5–C2–Cu1	117.96(17)
F5–C2–F6	105.10(19)
F5–C2–F4	107.3(2)
F6–C2–Cu1	108.84(16)
F6–C2–F4	106.8(2)
F4–C2–Cu1	110.15(16)
F1–C1–Cu1	110.26(16)
F1–C1–F3	105.5(2)
F2–C1–Cu1	113.58(17)
F2–C1–F1	106.0(2)
F2–C1–F3	107.2(2)
F3–C1–Cu1	113.69(17)
C6–C7–H7A	109.5
C6–C7–H7B	109.5
C6–C7–H7C	109.5
H7A–C7–H7B	109.5
H7A–C7–H7C	109.5
H7B–C7–H7C	109.5
C4–C3–H3A	109.5
C4–C3–H3B	109.5
C4–C3–H3C	109.5
H3A–C3–H3B	109.5
H3A–C3–H3C	109.5
H3B–C3–H3C	109.5
C8–C9–H9A	109.5
C8–C9–H9B	109.5
C8–C9–H9C	109.5
H9A–C9–H9B	109.5
H9A–C9–H9C	109.5
H9B–C9–H9C	109.5

Table 18. Torsion angles for 1a

Atom–Atom–Atom–Atom	Torsion Angle [°]
Cu1–O2–C6–C5	−1.6(3)
Cu1–O2–C6–C7	179.46(15)
Cu1–O1–C4–C5	0.2(3)
Cu1–O1–C4–C3	−179.04(15)
O2–Cu1–O1–C4	−2.33(18)
O2–C6–C5–C4	−1.6(4)
O1–Cu1–O2–C6	2.99(18)
O1–C4–C5–C6	2.3(4)
N1–Cu1–O2–C6	94.46(18)
N1–Cu1–O1–C4	−97.04(18)
C2–Cu1–O2–C6	−174.04(19)
C1–Cu1–O2–C6	−111.5(4)
C1–Cu1–O1–C4	166.38(18)
C7–C6–C5–C4	177.3(2)
C3–C4–C5–C6	−178.5(2)

[(Acac)Cu(CF_3)$_2$(DMF)] 1b: Complex **1** (20 mg) was dissolved in 10:1 DCM/DMF (0.5 ml) and the mixture was cooled down to 0°C, then layered with 2 ml of dry pentane. Crystallization of this mixture in the freezer at −40°C for 3 days afforded orange-red crystals of the solvate **1b**. Mother liquor was removed and the crystals were dried in vacuo at ambient temperature, then the X-ray crystal structure was recorded. CCDC 2546600.

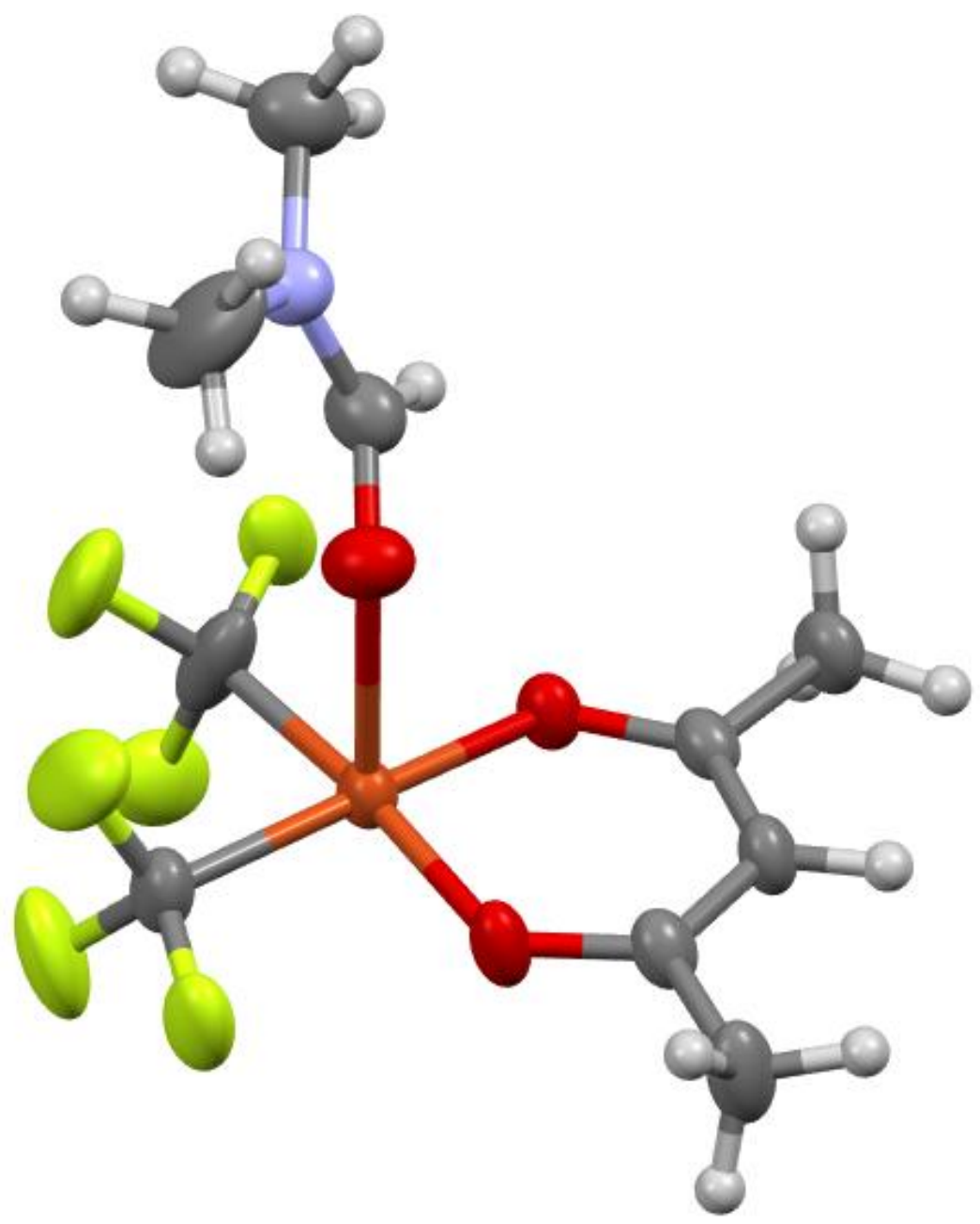

Figure S7. Crystal structure of [(acac)Cu(CF_3)$_2$(DMF)] ***1a***

Table 19. Crystal data and structure refinement for 1b

CCDC number	2546600
Empirical formula	$C_{10}H_{14}CuF_6NO_3$
Formula weight	373.76
Temperature [K]	150.00
Crystal system	monoclinic
Space group (number)	$P2_1/n$ (14)
a [Å]	8.9045(3)
b [Å]	12.3323(4)
c [Å]	13.4262(5)
α [°]	90
β [°]	104.8250(10)
γ [°]	90
Volume [Å^3]	1425.29(9)
Z	4
ρ_{calc} [gcm^{-3}]	1.742
μ [mm^{-1}]	1.608
F(000)	752
Crystal size [mm^3]	0.168×0.301×0.44
Crystal colour	orange
Crystal shape	block
Radiation	MoK_α (λ=0.71073 Å)
2θ range [°]	4.56 to 50.74 (0.83 Å)
Index ranges	$-10 \leq h \leq 10$<br>$-14 \leq k \leq 14$<br>$-16 \leq l \leq 16$
Reflections collected	34155
Independent reflections	2618<br>R_{int} = 0.0501<br>R_{sigma} = 0.0219
Completeness to θ = 25.242°	100.0 %
Data / Restraints / Parameters	2618 / 39 / 268
Absorption correction T_{min}/T_{max} (method)	0.4942 / 0.7452 (multi-scan)
Goodness-of-fit on F^2	1.043
Final R indexes [$I \geq 2\sigma(I)$]	R_1 = 0.0242<br>wR_2 = 0.0579
Final R indexes [all data]	R_1 = 0.0276<br>wR_2 = 0.0602
Largest peak/hole [eÅ^{-3}]	0.49/−0.32

Table 20. Bond lengths and angles for 1b

Atom–Atom	Length [Å]
Cu1–O1	1.8874(13)
Cu1–O2	1.8703(13)
Cu1–C1	1.947(2)
Cu1–C2	1.933(3)
Cu1–O3A	2.322(3)
Cu1–O3B	2.402(4)
O1–C4	1.278(2)
O2–C6	1.281(2)
F3–C1	1.330(2)
F2–C1	1.329(3)
F1–C1	1.333(3)
F6B–C2	1.383(9)
F5B–C2	1.276(5)
C4–C5	1.388(3)
C4–C3	1.499(3)
C5–H5	0.9500
C5–C6	1.388(3)
C6–C7	1.499(3)
F4B–C2	1.499(8)
C3–H3A	0.9800
C3–H3B	0.9800
C3–H3C	0.9800
C7–H7A	0.9800
C7–H7B	0.9800
C7–H7C	0.9800
C2–F4A	1.180(7)
C2–F5A	1.459(5)
C2–F6A	1.336(7)
C9B–H9BA	0.9800
C9B–H9BB	0.9800
C9B–H9BC	0.9800
C9B–N1B	1.41(3)
O3A–C8A	1.221(5)
C9A–H9AA	0.9800
C9A–H9AB	0.9800
C9A–H9AC	0.9800
C9A–N1A	1.487(16)
N1A–C8A	1.307(6)
N1A–C10A	1.45(3)
C8A–H8A	0.9500
O3B–C8B	1.214(6)
C8B–H8B	0.9500
C8B–N1B	1.313(6)
N1B–C10B	1.395(16)
C10B–H10A	0.9800
C10B–H10B	0.9800
C10B–H10C	0.9800
C10A–H10D	0.9800
C10A–H10E	0.9800
C10A–H10F	0.9800

Atom–Atom–Atom	Angle [°]
O1–Cu1–C1	170.47(8)
O1–Cu1–C2	87.44(8)
O1–Cu1–O3A	94.61(10)
O1–Cu1–O3B	87.54(9)
O2–Cu1–O1	95.48(6)
O2–Cu1–C1	89.61(7)
O2–Cu1–C2	174.00(12)
O2–Cu1–O3A	98.40(11)
O2–Cu1–O3B	79.96(11)
C1–Cu1–O3A	92.59(11)
C1–Cu1–O3B	101.30(11)
C2–Cu1–C1	86.77(10)
C2–Cu1–O3A	86.56(15)
C2–Cu1–O3B	105.44(15)
C4–O1–Cu1	124.49(12)
C6–O2–Cu1	124.49(12)
O1–C4–C5	125.13(17)
O1–C4–C3	114.83(17)
C5–C4–C3	120.04(17)
C4–C5–H5	118.0
C6–C5–C4	124.07(17)
C6–C5–H5	118.0
O2–C6–C5	125.53(18)
O2–C6–C7	114.08(17)
C5–C6–C7	120.37(18)
C4–C3–H3A	109.5
C4–C3–H3B	109.5
C4–C3–H3C	109.5
H3A–C3–H3B	109.5
H3A–C3–H3C	109.5
H3B–C3–H3C	109.5
F3–C1–Cu1	111.49(13)
F3–C1–F1	104.49(18)
F2–C1–Cu1	113.20(15)
F2–C1–F3	105.04(18)
F2–C1–F1	108.40(19)
F1–C1–Cu1	113.53(15)
C6–C7–H7A	109.5
C6–C7–H7B	109.5
C6–C7–H7C	109.5
H7A–C7–H7B	109.5
H7A–C7–H7C	109.5
H7B–C7–H7C	109.5
F6B–C2–Cu1	113.0(5)
F6B–C2–F4B	114.5(5)
F5B–C2–Cu1	119.5(3)
F5B–C2–F6B	104.4(6)
F5B–C2–F4B	99.0(4)
F4B–C2–Cu1	106.0(3)
F4A–C2–Cu1	115.0(4)
F4A–C2–F5A	108.7(4)
F4A–C2–F6A	101.7(5)
F5A–C2–Cu1	100.4(2)
F6A–C2–Cu1	123.0(5)
F6A–C2–F5A	107.4(4)
H9BA–C9B–H9BB	109.5
H9BA–C9B–H9BC	109.5
H9BB–C9B–H9BC	109.5
N1B–C9B–H9BA	109.5

N1B–C9B–H9BB	109.5
N1B–C9B–H9BC	109.5
C8A–O3A–Cu1	124.1(3)
H9AA–C9A–H9AB	109.5
H9AA–C9A–H9AC	109.5
H9AB–C9A–H9AC	109.5
N1A–C9A–H9AA	109.5
N1A–C9A–H9AB	109.5
N1A–C9A–H9AC	109.5
C8A–N1A–C9A	116.7(8)
C8A–N1A–C10A	121.3(12)
C10A–N1A–C9A	122.0(14)
O3A–C8A–N1A	125.9(4)
O3A–C8A–H8A	117.0
N1A–C8A–H8A	117.0
C8B–O3B–Cu1	119.1(3)
O3B–C8B–H8B	117.2
O3B–C8B–N1B	125.5(5)
N1B–C8B–H8B	117.2
C8B–N1B–C9B	119.8(15)
C8B–N1B–C10B	121.8(9)
C10B–N1B–C9B	118.0(18)
N1B–C10B–H10A	109.5
N1B–C10B–H10B	109.5
N1B–C10B–H10C	109.5
H10A–C10B–H10B	109.5
H10A–C10B–H10C	109.5
H10B–C10B–H10C	109.5
N1A–C10A–H10D	109.5
N1A–C10A–H10E	109.5
N1A–C10A–H10F	109.5
H10D–C10A–H10E	109.5
H10D–C10A–H10F	109.5
H10E–C10A–H10F	109.5

Table 21. Torsion angles for 1b

Atom–Atom–Atom–Atom	Torsion Angle [°]
Cu1–O1–C4–C5	5.1(3)
Cu1–O1–C4–C3	−174.80(13)
Cu1–O2–C6–C5	−5.0(3)
Cu1–O2–C6–C7	175.98(15)
Cu1–O3A–C8A–N1A	147.6(4)
Cu1–O3B–C8B–N1B	−133.0(4)
O1–Cu1–O2–C6	8.86(17)
O1–C4–C5–C6	2.0(3)
O2–Cu1–O1–C4	−8.93(16)
C4–C5–C6–O2	−2.1(3)
C4–C5–C6–C7	176.9(2)
C3–C4–C5–C6	−178.1(2)
C1–Cu1–O2–C6	−179.21(17)
C2–Cu1–O1–C4	176.32(18)
O3A–Cu1–O1–C4	89.98(18)
O3A–Cu1–O2–C6	−86.64(18)
C9A–N1A–C8A–O3A	0.5(11)
O3B–Cu1–O1–C4	70.74(18)
O3B–Cu1–O2–C6	−77.66(18)
O3B–C8B–N1B–C9B	5.0(17)
O3B–C8B–N1B–C10B	177.3(9)
C10A–N1A–C8A–O3A	179.4(15)

DFT calculations

All calculations were performed in the ORCA 6.1.0 software[8] (defgrid 3) for single state of all copper(III) complexes owing to their diamagnetic properties. Initial geometries were taken from the XRD structures.

1. Lewis acidity according to Greb's method. The initial optimization of geometries for compounds was done at the B3LYP level[9] with Zero-Order Regular Approximation relativistic Hamiltonian[10] using the ZORA-def2-TZVP[11] basis set with a D3(BJ)[12] dispersion correction. Final results of optimization contain no imaginary frequencies. The single point energy calculations were done at the ωB97X-D3(BJ)[12]/def2-TZVPPD level of theory. Calculation of the Lewis acidity was done using the TMS^+/TMSF as an anchor point, as in the method developed by Greb and coworkers.[13] The geometries and single point energies of TMS^+ and TMSF were calculated on the same level mentioned above. Thermal corrections (ZPE differences at B3LYP-D3(BJ)/ZORA-def2-TZVP) were included in the calculation. All calculations were performed in the gas phase without solvation corrections.

2. Lewis acidity according to Kaupp's method. The initial optimization of geometries (tightOPT, tightSCF) and thermochemistry for compounds was done at the ωB97X-D3(BJ)[12]/def2-TZVPD level of theory. Calculation of the Lewis acidity was done via direct comparison of enthalpies of formation of

acetylacetonate and fluoride adduct as suggested by Kaupp.[14] Thermochemistry of a fluoride anion was calculated on the same level. All calculations were performed in the gas phase without solvation corrections.

Table 22. Comparison of FIA for copper(III), (II) and (I) acetylacetonates

Complex	FIA, kJ/mol
(acac)Cu(CF_3)$_2$	225
(acac)$_2$Cu	160
(acac)Cu(IPr)	97

3. ΔH and ΔG calculations in DCM solution. Geometries of all compounds for thermodynamic parameter calculations were calculated on the same level of theory as used for the Greb's method (B3LYP[9]-D3(BJ)[12]/ZORA-def2-TZVP[11]) *with the solvent environment represented as SMD(DCM)[15] solvation model, incorporated both during optimization of geometry and single point energy calculations*. Vibrational frequencies and thermochemistry were calculated on the same level as geometries at two different temperatures (298K and 233K); final results of optimization contain no imaginary frequencies. The single point energy calculations were done at the ωB97X-D3(BJ)[12]/def2-QZVPPD level of theory. Thermal corrections (differences of **all** inner energy parameters) were included in the calculation (H = E(el) + E(ZPE) + E(vibr) + E(rot) + E(transl) + kB*T). Pyridine adducts for the Hammett analysis of the electronic effects of the Lewis base were modelled on the same level of theory as other adducts (ωB97X-D3(BJ)[12]/def2-QZVPPD//B3LYP[9]-D3(BJ)[12]/ZORA-def2-TZVP[11], SMD(DCM).

All computed geometries can be found in the separately provided .xyz files.

4. **NBO calculations.** Natural bonding orbital (NBO)[16] analysis including NPA charge calculations, WBI and NBI parameters, was performed on ωB97X-D3(BJ)/def2-QZVPPD level of theory using the B3LYP-D3(BJ)/ZORA-def2-TZVP optimal geometries. Validation of these results was done on the "gold-standard" coupled-cluster DLPNO-CCSD(T)[17]/def2-TZVP level of theory and a double-hybrid functional while keeping the same basis set: B2PLYP[18]-D3(BJ)/def2-QZVPPD. In both cases the validation results corroborated the originally observed trend on the ωB97X-D3(BJ)/def2-QZVPPD level of theory. The absolute value of NPA charge on high-valent copper is notably higher for calculations at the DLPNO-CCSD(T)[17]/def2-TZVP level of theory compared to other two methods.

Table 23. NBO parameters for all complexes calculated with a wB97X functional

Complex	Number	τ_5	NPA (Cu)	WBI (Cu-L)	NBI (Cu-L)	WBI (Cu-C) median	Δ(WBI)	NBI (Cu-C) median	Δ(NBI)	τ4/[τ4]
(acac)Cu(CF_3)$_2$	**1**	-	1.119	-	-	0.529	-	0.728	-	0.059
(acac)Cu(CF_3)$_2$(Py)	**2**	0.033	1.178	0.051	0.226	0.518	0.011	0.719	0.009	0.200
(acac)Cu(CF_3)$_2$(*i*PrNH_2)	**3**	0.117	1.162	0.182	0.426	0.513	0.016	0.716	0.012	0.117
(acac)Cu(CF_3)$_2$(Et_3PO)	**4**	0.095	1.191	0.046	0.213	0.518	0.011	0.720	0.008	0.153
(acac)Cu(CF_3)$_2$(DMF)	**1b**	0.059	1.187	0.042	0.206	0.519	0.01	0.720	0.008	0.110
(acac)Cu(CF_3)$_2$(MeCN)	**1a**	0.138	1.172	0.041	0.203	0.517	0.012	0.719	0.009	0.117
(acac)Cu(CF_3)$_2$(Cl)$^-$	**5**	0.343	1.179	0.121	0.347	0.513	0.016	0.716	0.012	0.228

Validation of NBO parameters using different basis sets and methods:

Original trend with a wB97X functional:

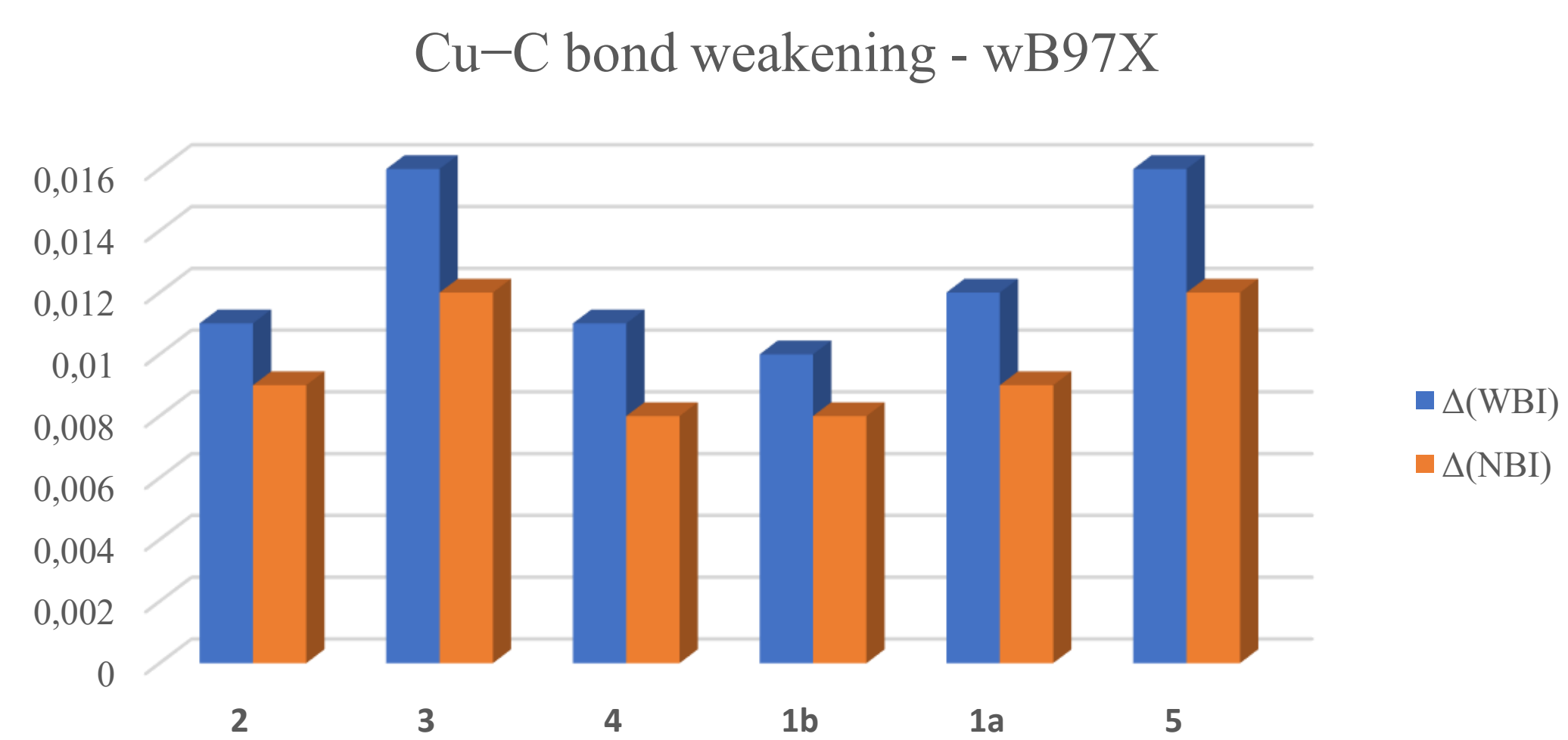

Table 24. Validation of parameters for complexes computed on the DLPNO-CCSD(T)/def2-TZVP level of theory

Complex	NPA (Cu)	WBI (Cu-L)	NBI (Cu-L)	WBI (Cu-C) median	NBI (Cu-C) median
**1**	1.379	-	-	0.607	0.779
**2**	1.443	0.041	0.203	0.600	0.775

**3**	1.428	0.182	0.427	0.592	0.769
**4**	1.459	0.037	0.192	0.597	0.774
**1b**	1.465	0.033	0.182	0.597	0.773
**1a**	1.432	0.032	0.178	0.598	0.774
**5**	1.457	0.106	0.326	0.601	0.773

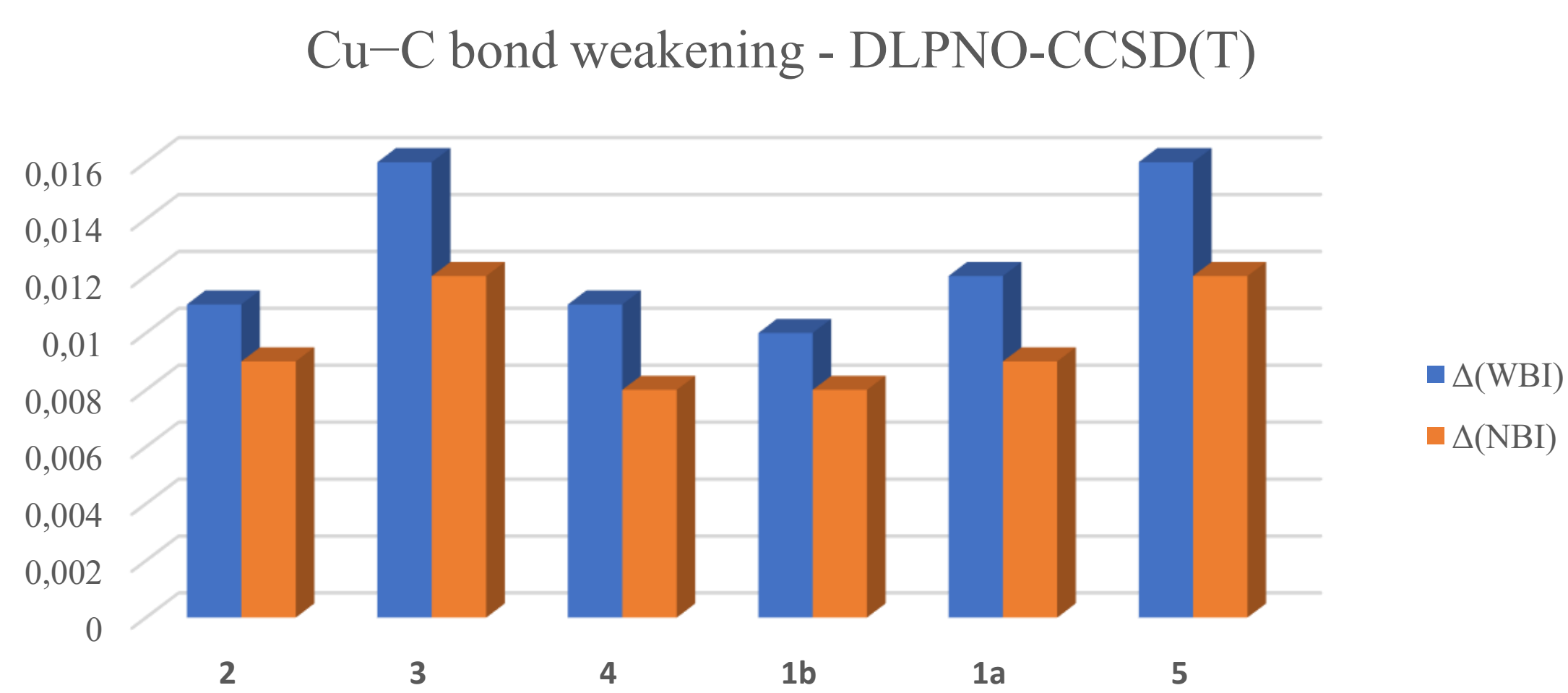

Table 25. Validation of parameters for complexes computed on the B2PLYP-D3(BJ)/def2-QZVPPD (def2-QZVPPD/C as an auxiliary basis set)

Complex	NPA (Cu)	WBI (Cu-L)	NBI (Cu-L)	WBI (Cu-C) median	NBI (Cu-C) median
**1**	1.182	-	-	0.549	0.741
**2**	1.248	0.048	0.219	0.538	0.733
**3**	1.232	0.185	0.430	0.531	0.729
**4**	1.262	0.044	0.209	0.539	0.734
**1b**	1.259	0.040	0.199	0.538	0.734
**1a**	1.241	0.038	0.195	0.536	0.732
**5**	1.253	0.116	0.341	0.533	0.730

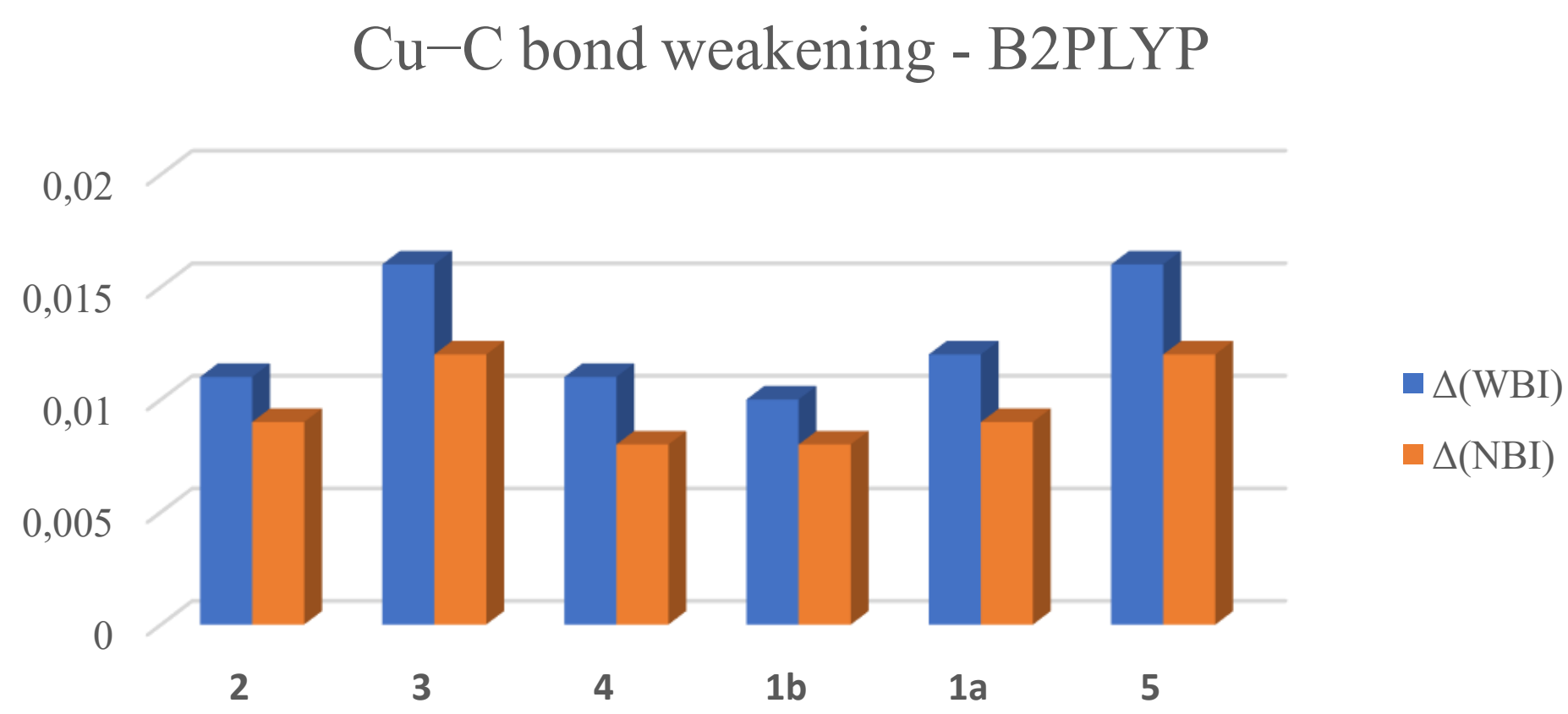

References

1. V. Motornov, N. Limberg, *Chem. Eur. J.* **2026**, 32(5), e03417.
2. Bruker, *SAINT*, Bruker AXS SE, Karlsruhe, Germany.
3. L. Krause, R. Herbst-Irmer, G. M. Sheldrick, D. Stalke, *J. Appl. Cryst.* **2015**, *48*, 3–10, doi:10.1107/S1600576714022985.
4. G. M. Sheldrick, *Acta Cryst.* **2015**, *A71*, 3–8, doi:10.1107/S2053273314026370.
5. G. M. Sheldrick, *Acta Cryst.* **2015**, *C71*, 3–8, doi:10.1107/S2053229614024218.
6. C. R. Groom, I. J. Bruno, M. P. Lightfoot, S. C. Ward, *Acta Cryst.* **2016**, *B72*, 171–179, doi:10.1107/S2052520616003954.
7. D. Kratzert, *FinalCif*, *V152*, https://dkratzert.de/finalcif.html.
8. a) Neese, F. Software update: the ORCA program system, version 6.0, WIRES *Comput. Molec. Sci.* **2025**, 15, e70019, DOI: 10.1002/wcms.7019. b) Neese, F., *J. Comp. Chem*. **2003** *24*, 1740-1747, DOI: 10.1002/jcc.10318; c) Neese, F.; Wennmohs, F.; Hansen, A.; Becker, U. Chem. Phys. 2009 356, 98-109; d) Grimme, S.; Antony, J.; Ehrlich, S.; Krieg, H. *J. Chem. Phys*. **2010** *132*, 154104; e) Grimme, S.; Ehrlich, S.; Goerigk, L. *J. Comput. Chem.* **2011** *32*, 1456-1465; f) Helmich-Paris, B.; de Souza, B.; Neese, F.; Izsák, R. *J. Chem. Phys.* **2021** *155*, 104109; g) Neese, F. *J. Comp. Chem.* **2022** *44*, 381.
9. a) C. Lee, W. Yang, R. G. Parr, *Physical review. B, Condensed matter* **1988**, 37, 785; b) A. D. Becke, *Physical review. A, General physics* **1988**, *38*, 3098.
10. a) M. Douglas, N. M. Kroll, *Ann. Phys*., **1974**, *82*, 89. b) B. A. Hess, *Phys. Rev. A*, **1985**, *32*, 756. c) D. A. Pantazis, X.-Y. Chen, C. R. Landis, F. Neese, *J. Chem. Theor. Comput*., **2008**, *4*, 908.
11. a) F. Weigend, *Phys. Chem. Chem. Phys.* **2006**, *8*, 1057; b) F. Weigend, R. Ahlrichs, *Phys. Chem. Chem. Phys.* **2005**, *7*, 3297.
12. a) G. Brandenburg, J. W. Ochterski, K. A. Peterson, *J. Chem. Phys*. **2007**, *127*, 154108. b) M. Sierka, A. Hogekamp, R. Ahlrichs, *J. Chem. Phys*. **2003**, *118*, 9136; c) S. Grimme, S. Ehrlich, L. Goerigk, *J. Comput. Chem*. **2011**, *32*, 1456; d) S. Grimme, J. Antony, S. Ehrlich, H. Krieg, *J. Chem. Phys.* **2010**, *132*, 154104.
13. a) P. Erdmann, J. Leitner, J. Schwarz, L. Greb, *ChemPhysChem* **2020**, 21, 987–994. b) P. Erdmann, L. Greb, *ChemPhysChem*, **2021**, *22*, 935-943.
14. C. Lehmann, S. N. Balogun, M. Reimann, M. Kaupp, *Chem. Eur. J.* **2025**, *31*, e202404662.
15. A. V. Marenich, C. J. Cramer, D. G. Truhlar, *J. Phys. Chem. B*, **2009**, *113*, 6378.
16. E. D. Glendening, C. R. Landis, F. Weinhold, *J. Comput. Chem*. **2019**, *40*, 2234.
17. C. Riplinger and F. Neese, *J. Chem. Phys.*, **2013**, 138, 034106.
18. S. Grimme and F. Neese, *J. Chem. Phys.* **2007**, 127, 154116.

NMR spectra

[(acac)Cu(CF$_3$)$_2$(Py)] 2

^{1}H NMR (CDCl$_3$, 400 MHz, 293 K)

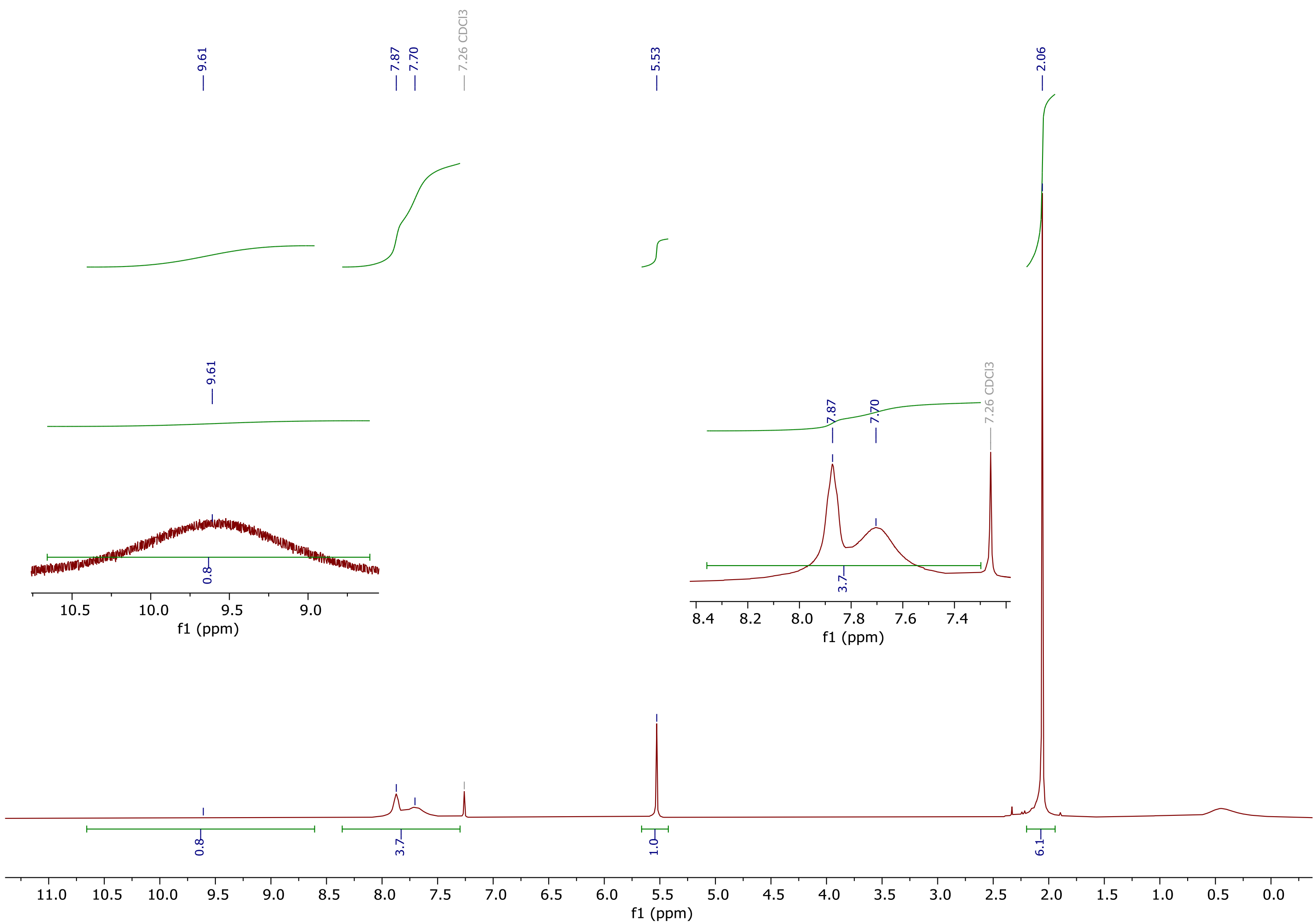

Stacked ^{1}H NMR spectra of free pyridine and an adduct **2** in 0.1 M CD_2Cl_2 at different temperatures

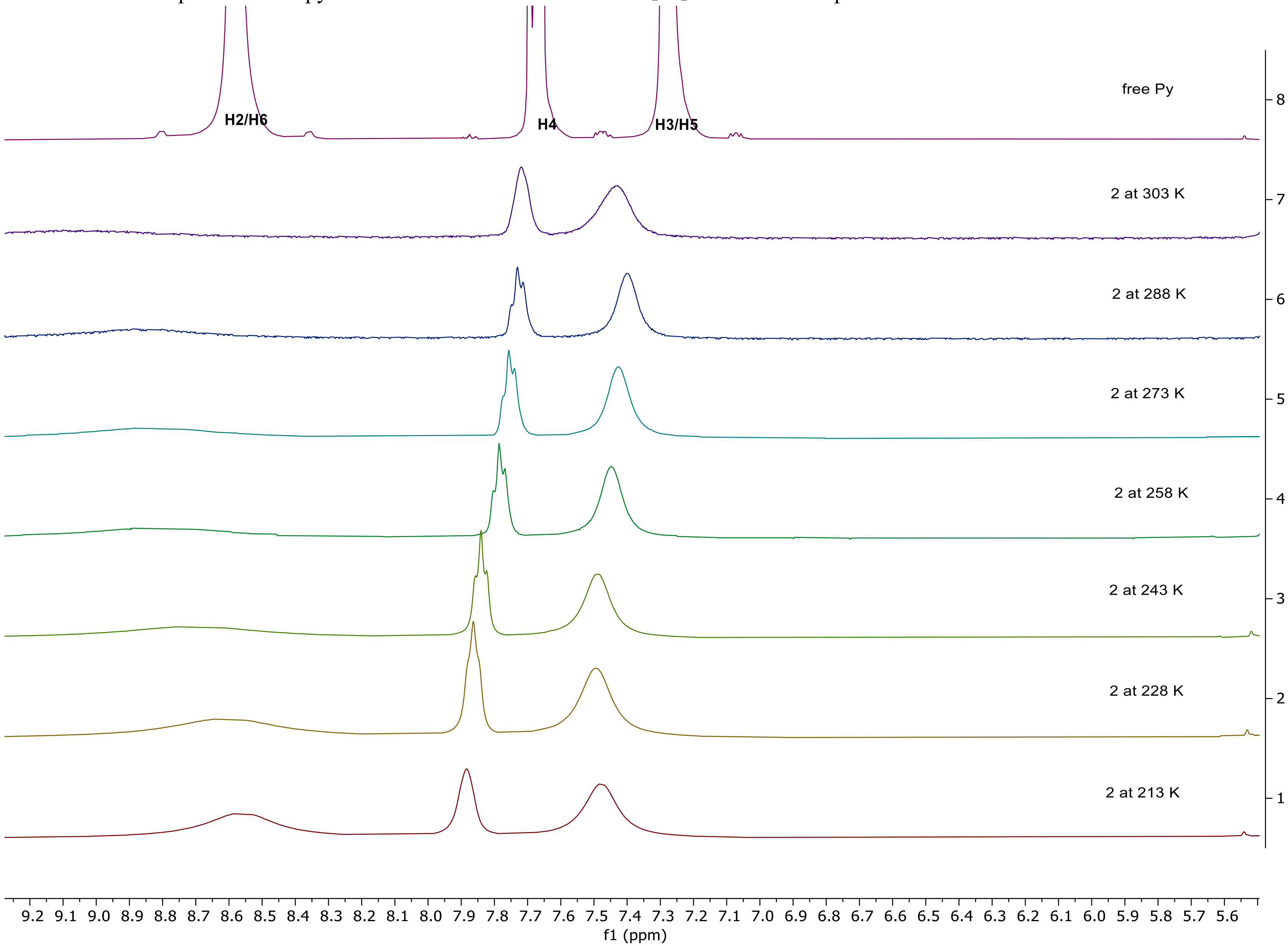

^{1}H NMR (CD_2Cl_2, 400 MHz, 198 K)

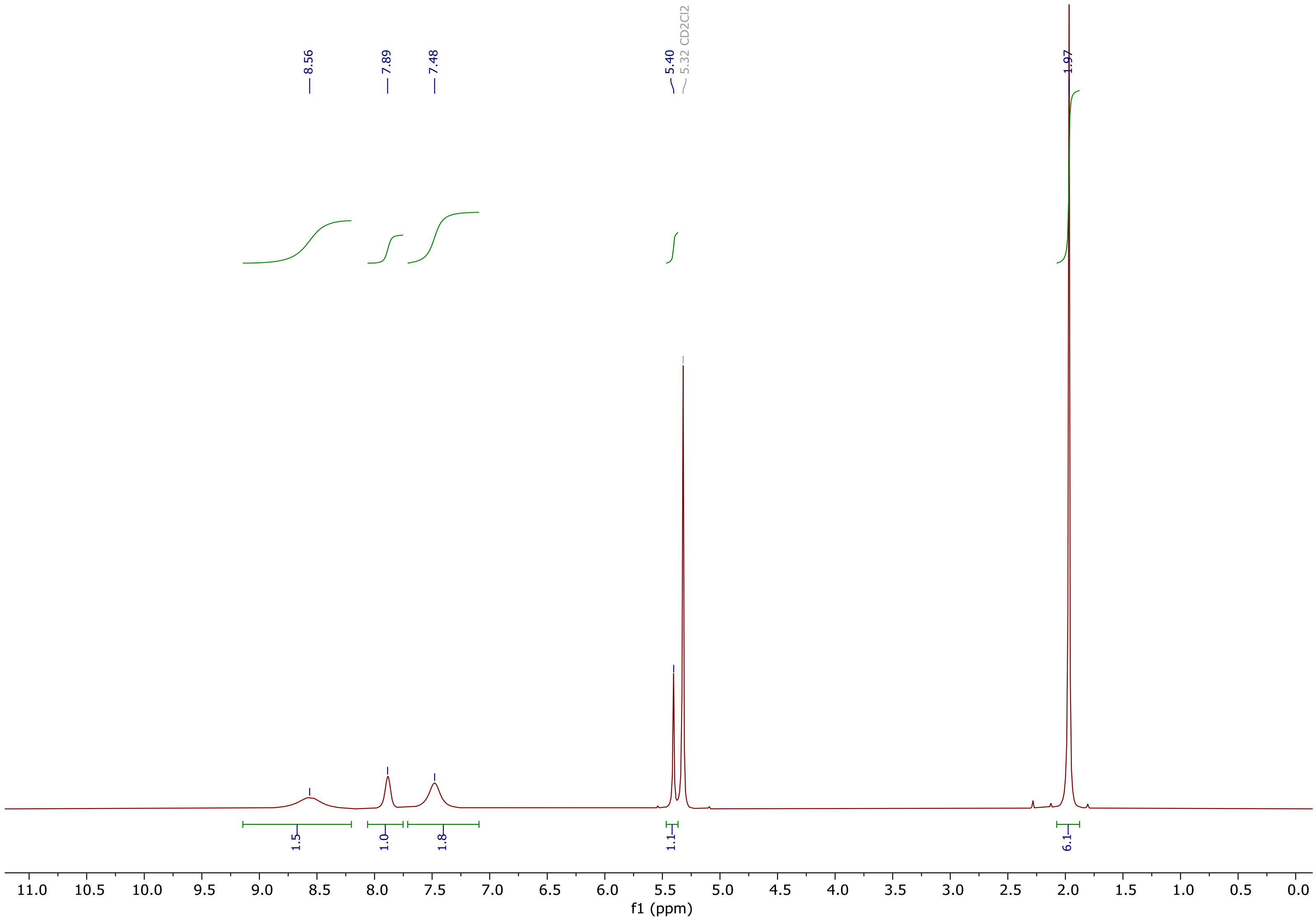

^1H NMR (CD_2Cl_2, 400 MHz, 213 K)

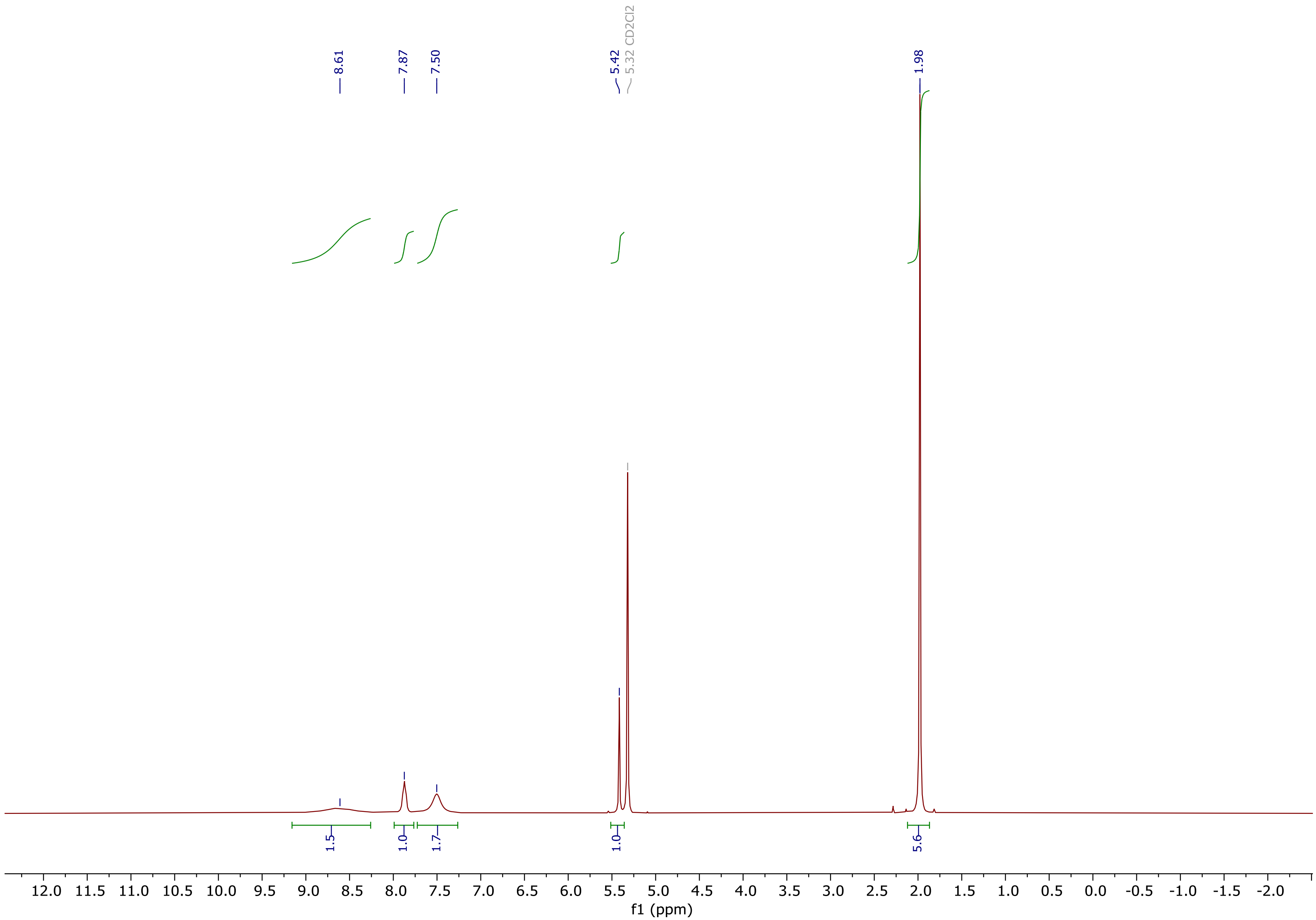

^{1}H NMR (CD_2Cl_2, 400 MHz, 228 K)

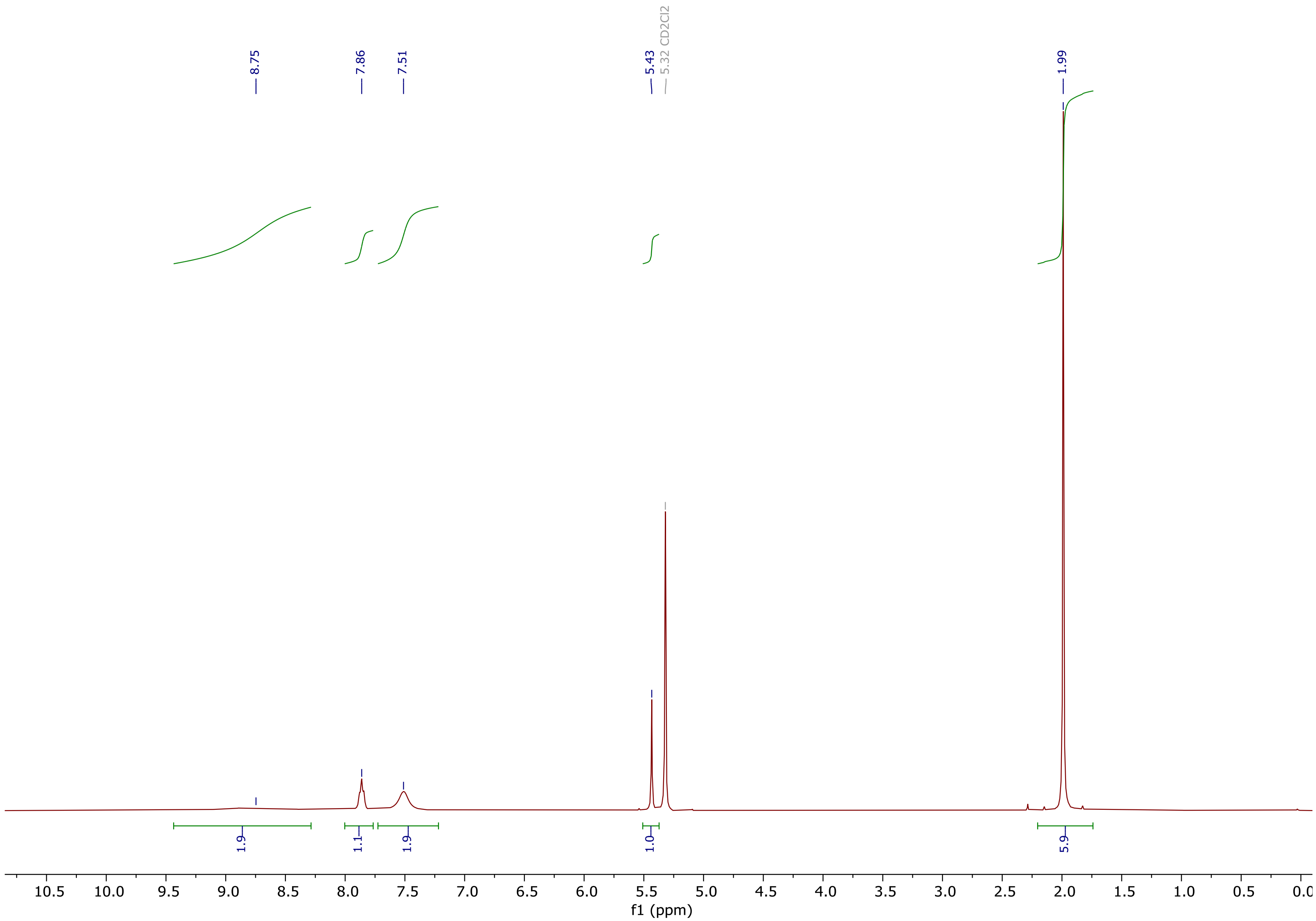

^1H NMR (CD_2Cl_2, 400 MHz, 243 K)

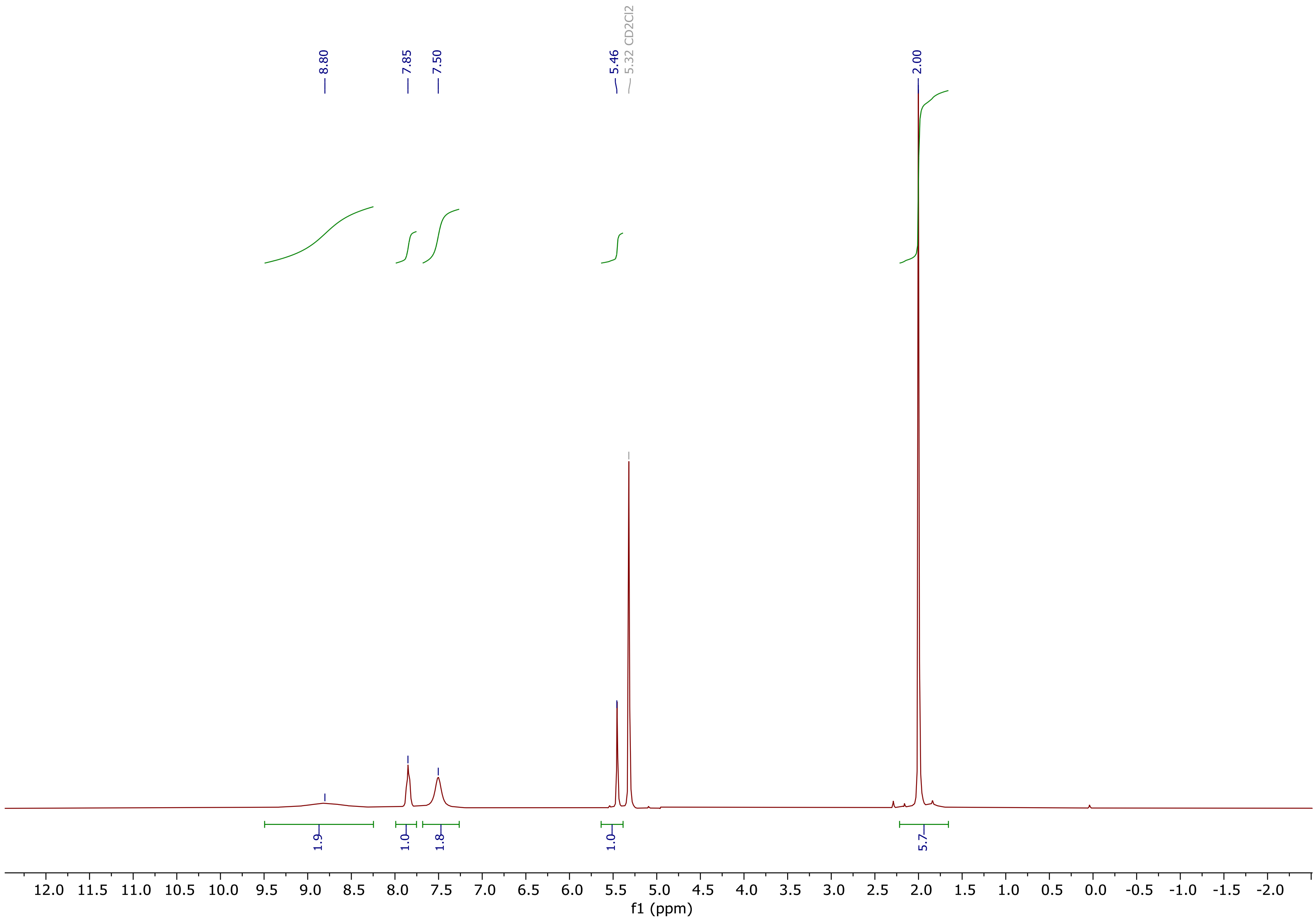

^{1}H NMR (CD_2Cl_2, 400 MHz, 258 K)

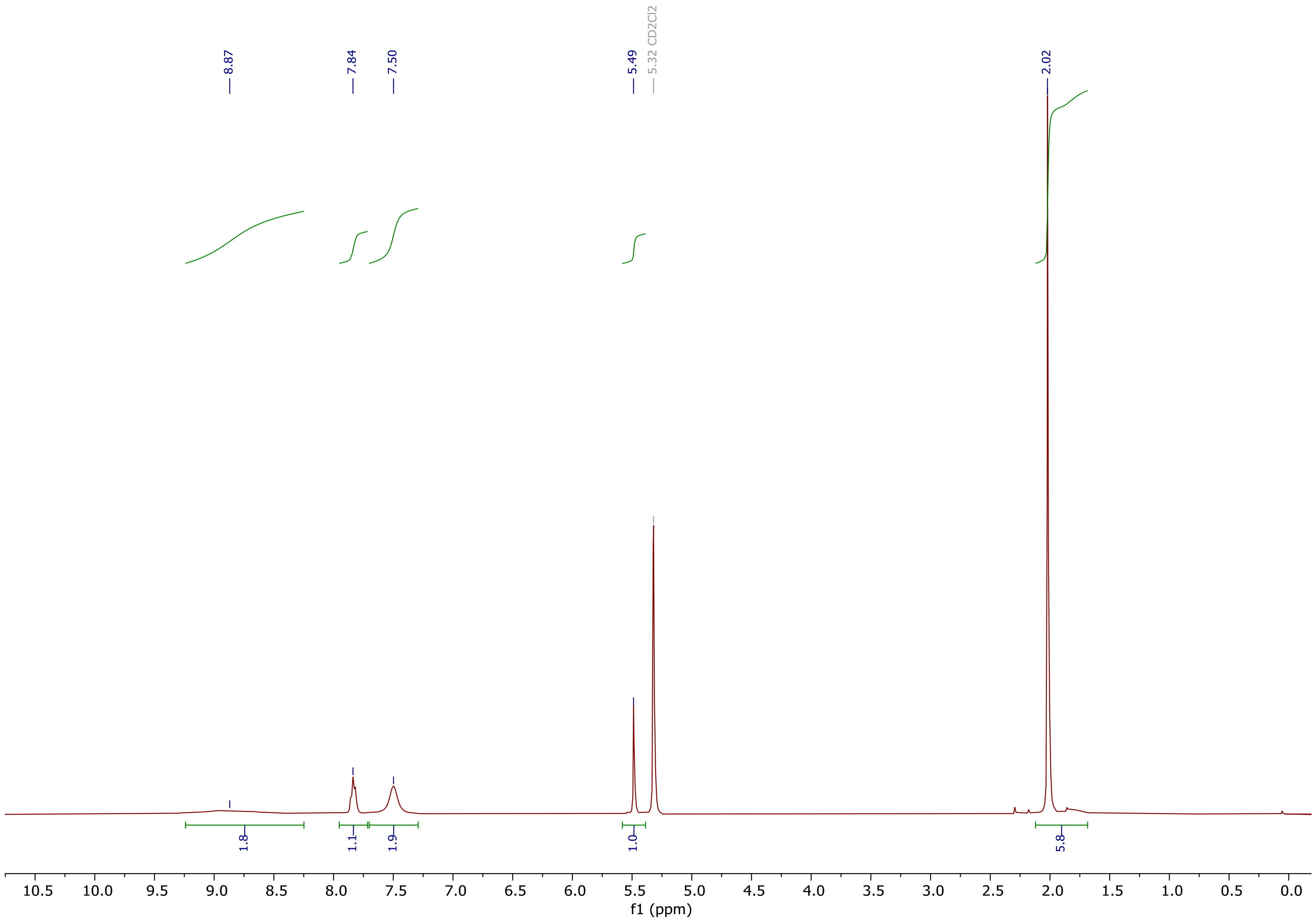

^{1}H NMR (CD_2Cl_2, 400 MHz, 273 K)

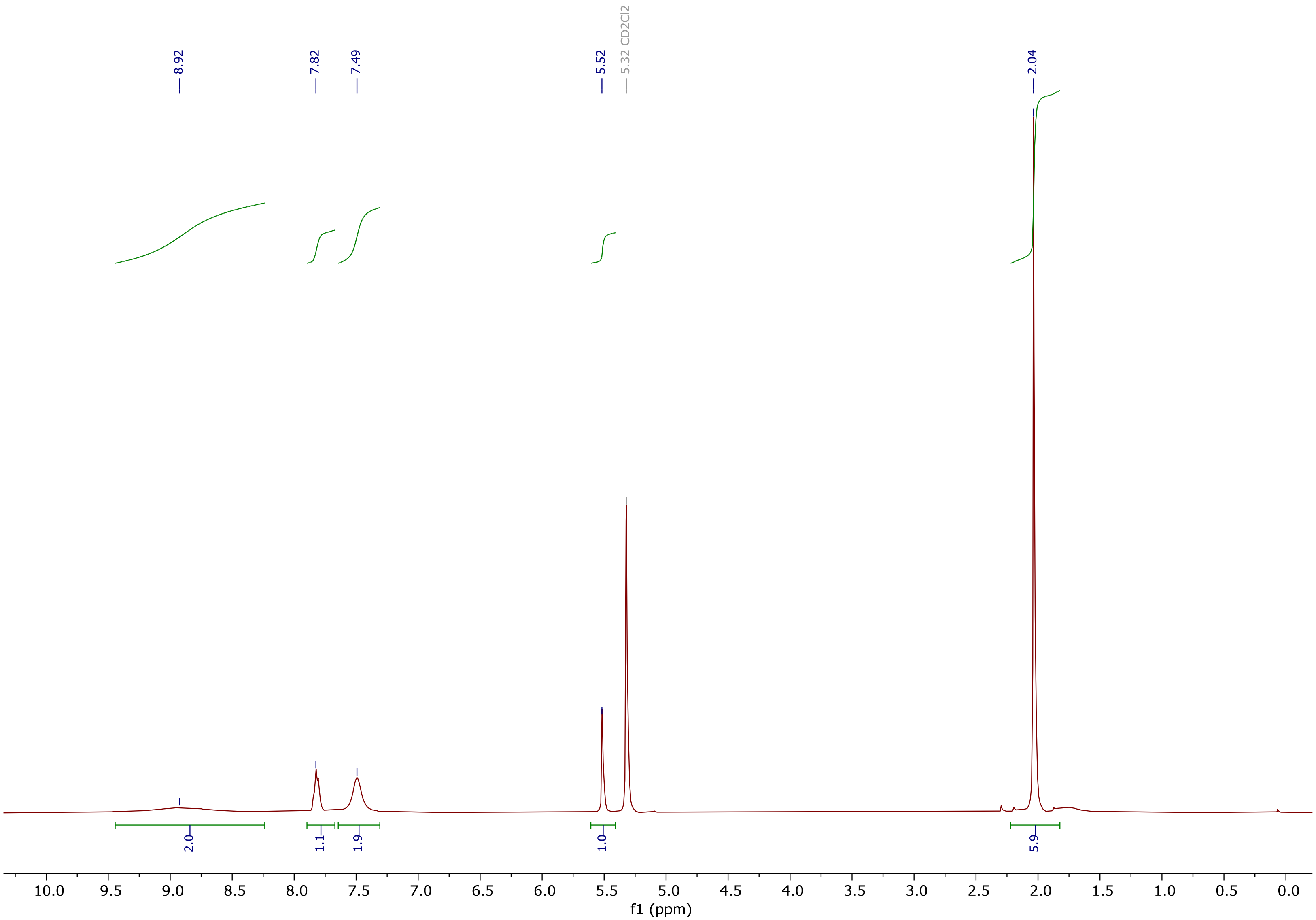

^{1}H NMR (CD_2Cl_2, 400 MHz, 288 K)

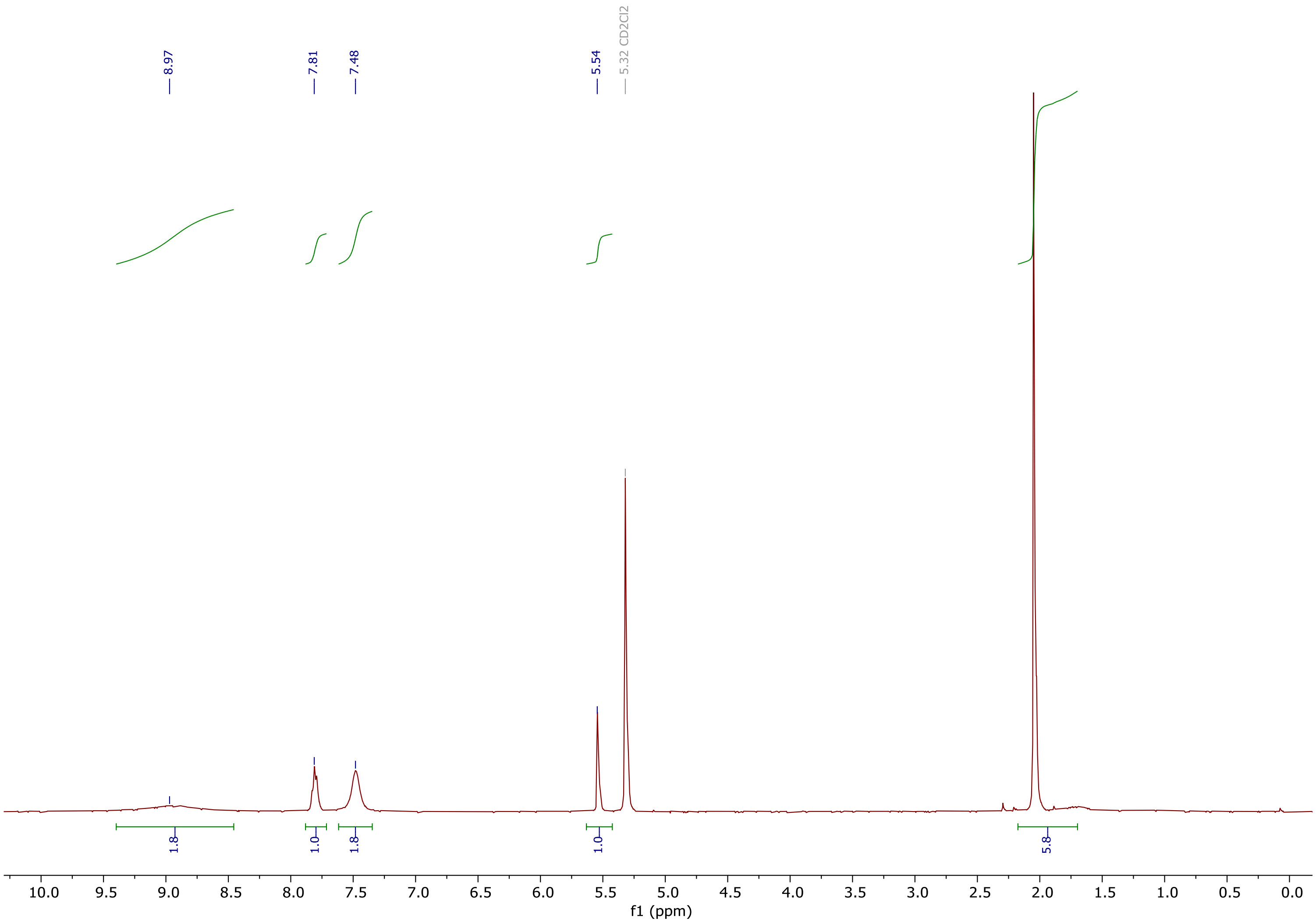

^{1}H NMR (CD_2Cl_2, 400 MHz, 303 K)

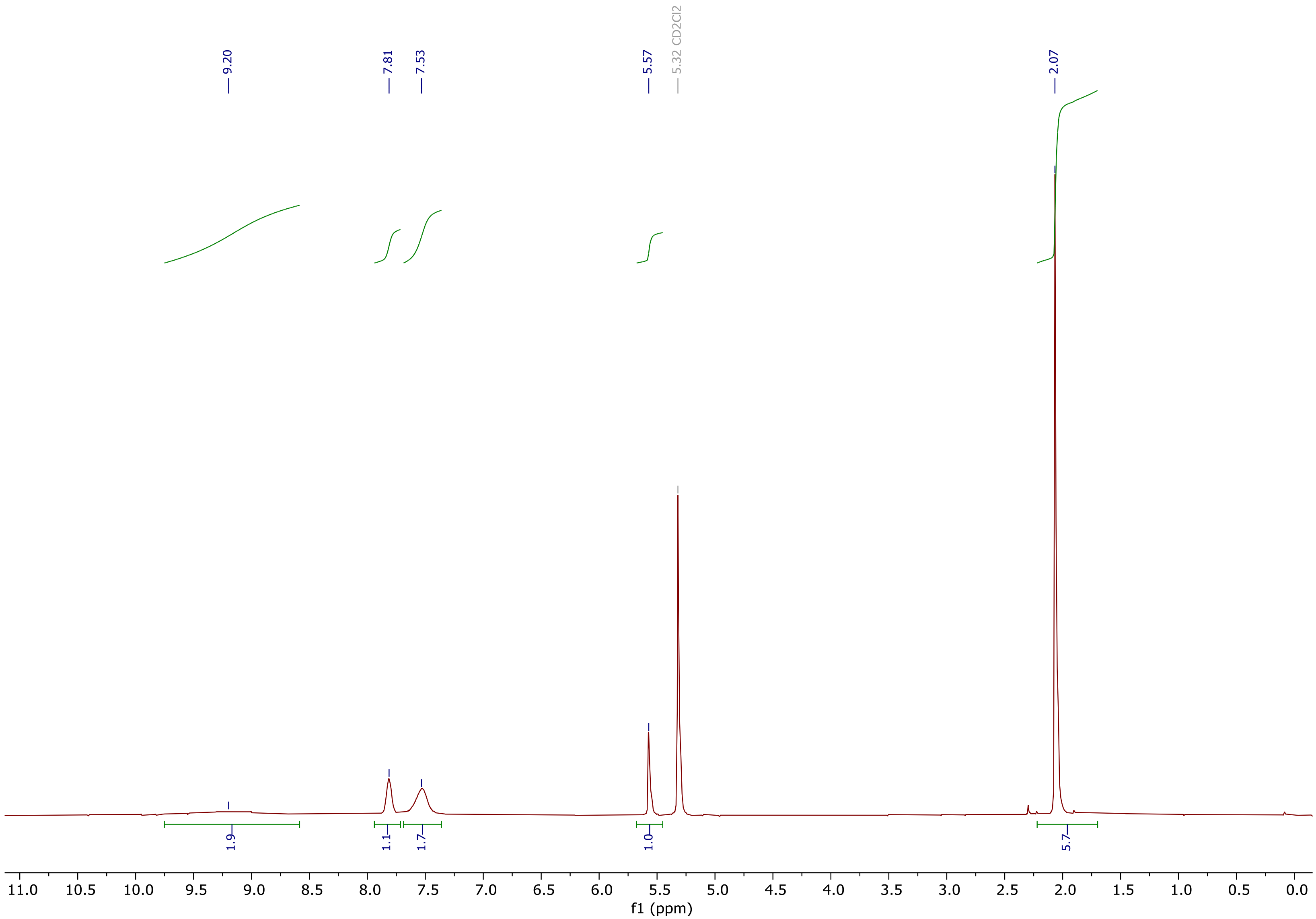

^{19}F NMR ($CDCl_3$, 376 MHz, 293 K)

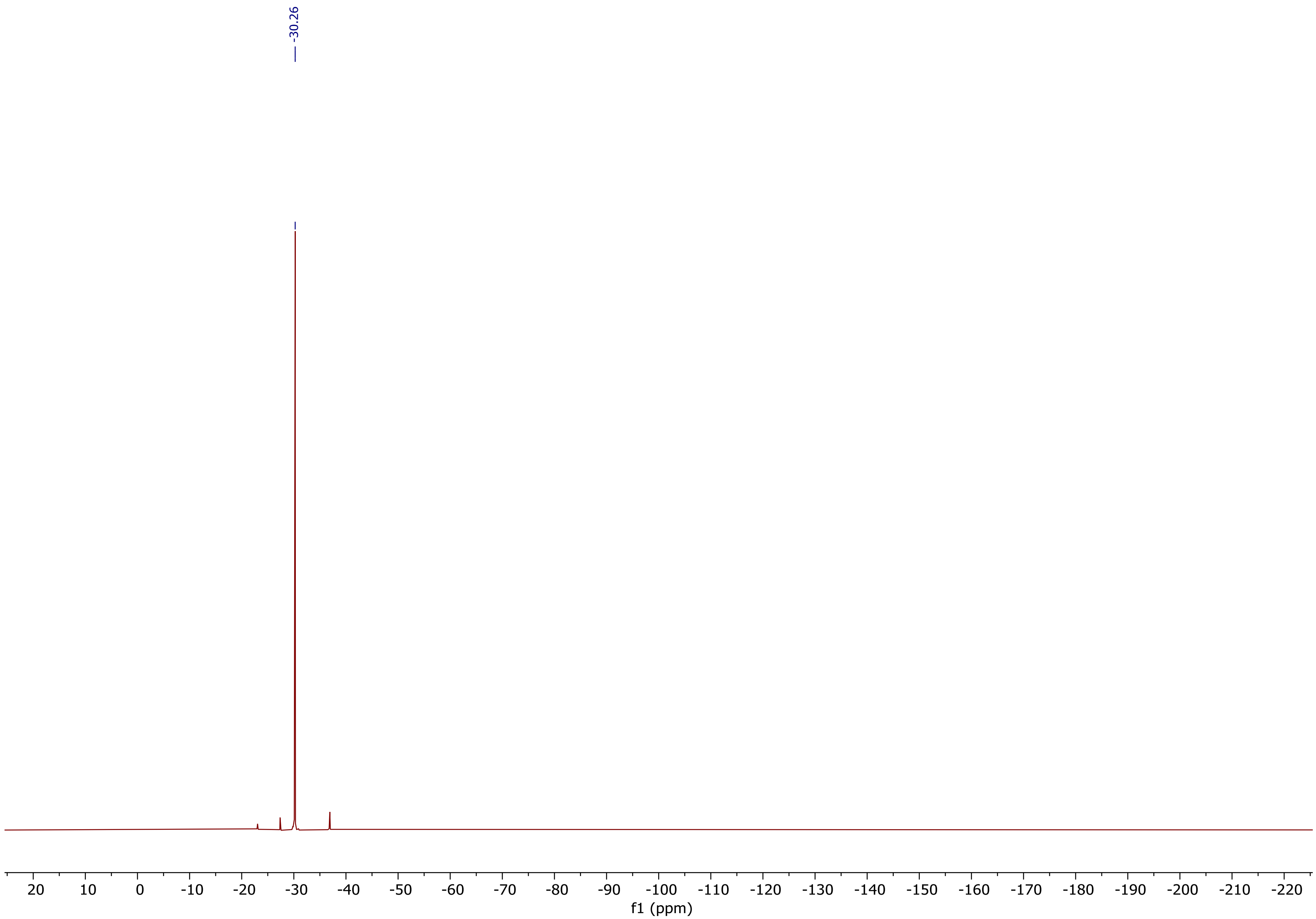

[(acac)Cu(CF_3)$_2$(iPrNH$_2$)] 3

^{1}H NMR (benzene-d$_6$, 400 MHz, 293 K)

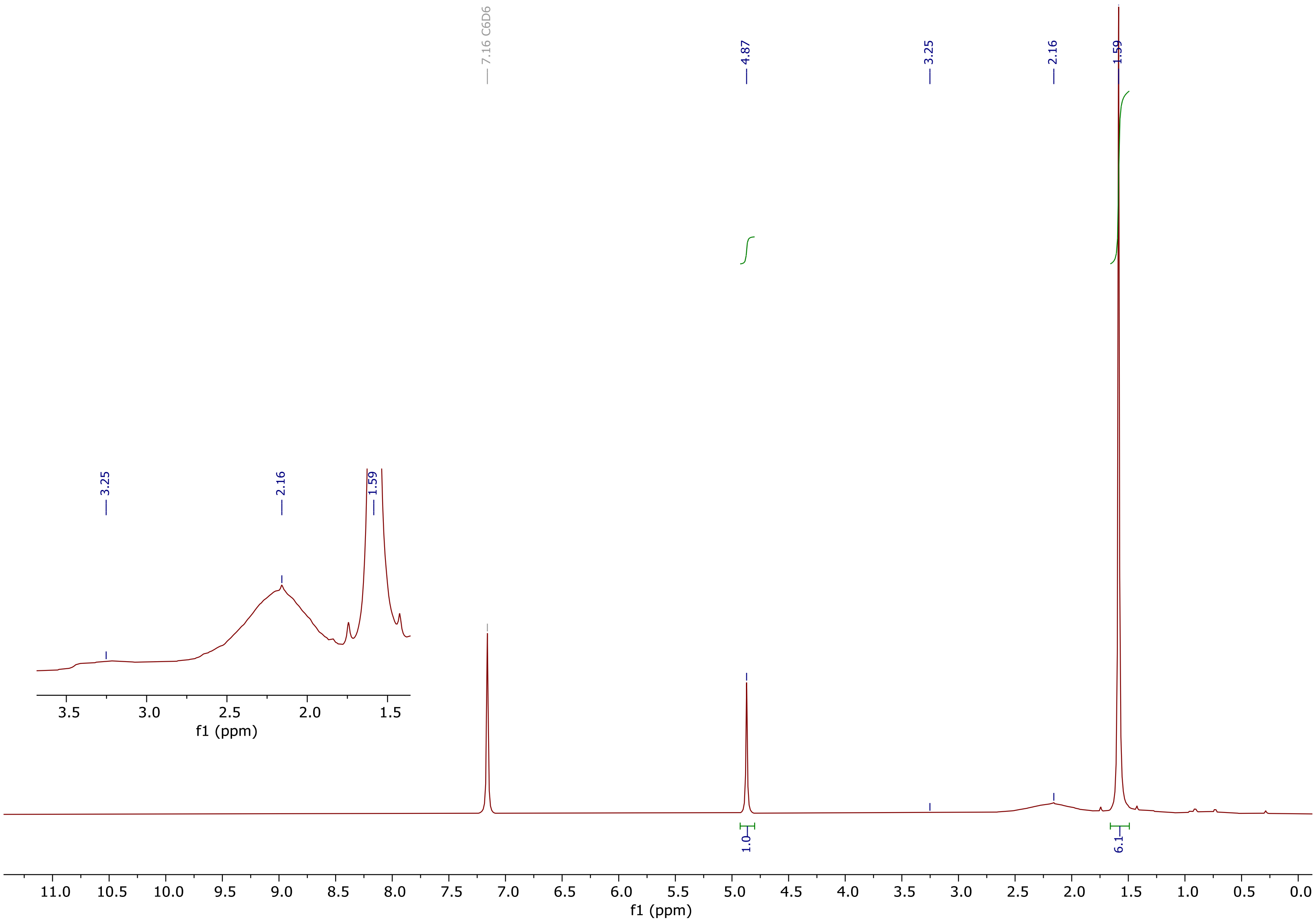

^{13}C NMR (CD_3CN, 101 MHz, 293 K)

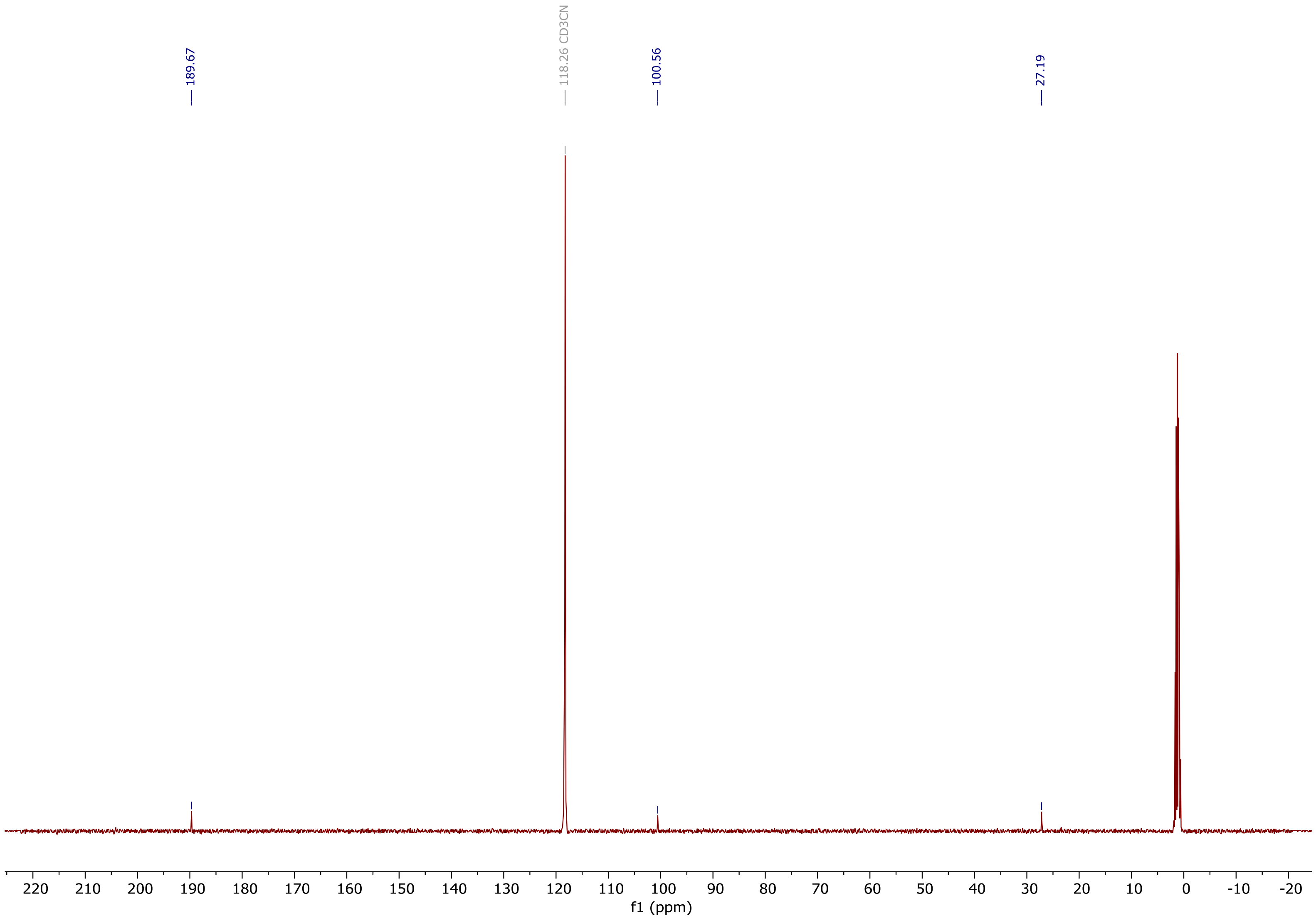

^{19}F NMR (CD_3CN, 376 MHz, 293 K)

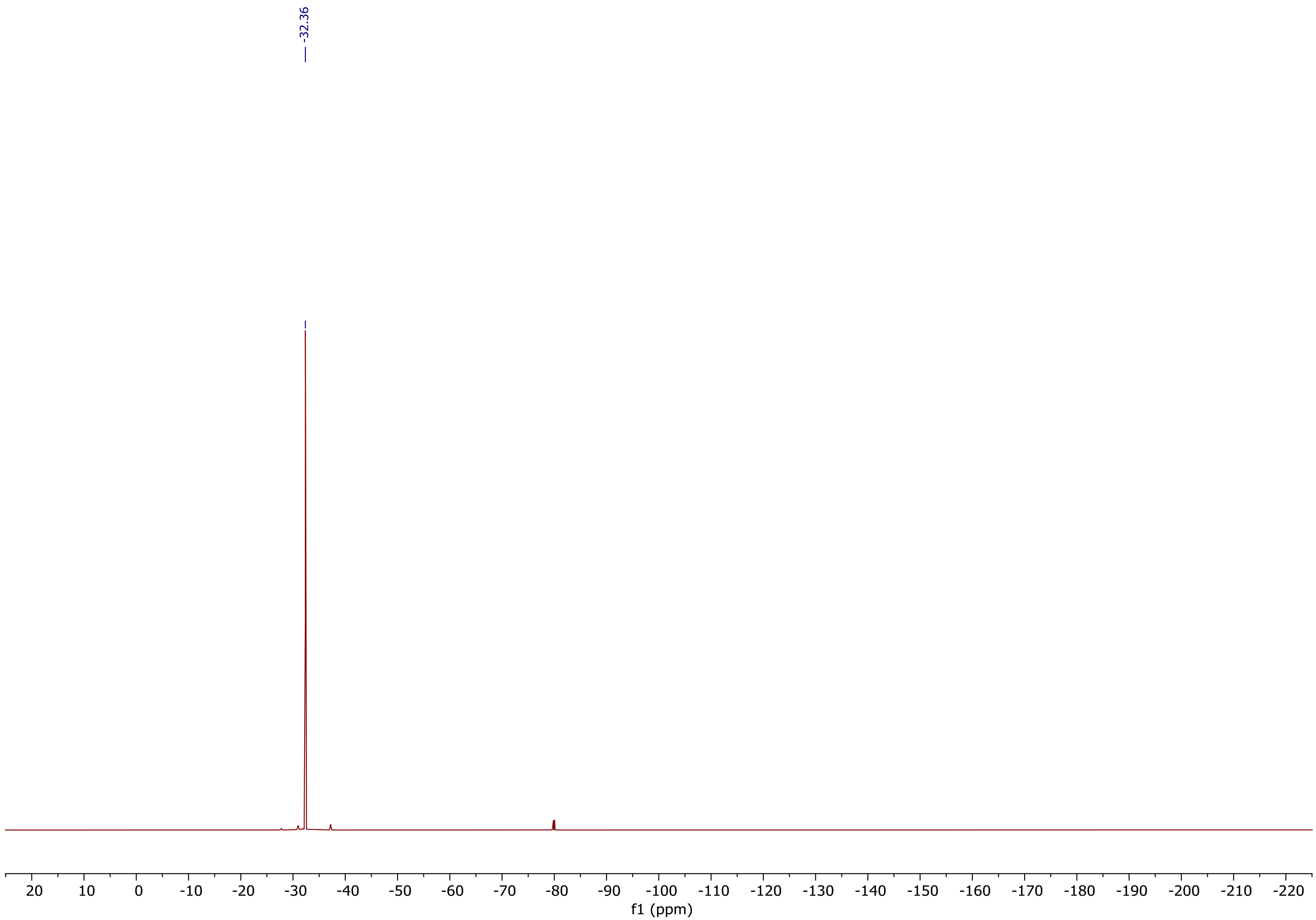

[(acac)Cu(CF$_3$)$_2$(TEPO)] 4

^{1}H NMR (CD$_3$CN, 400 MHz, 293 K)

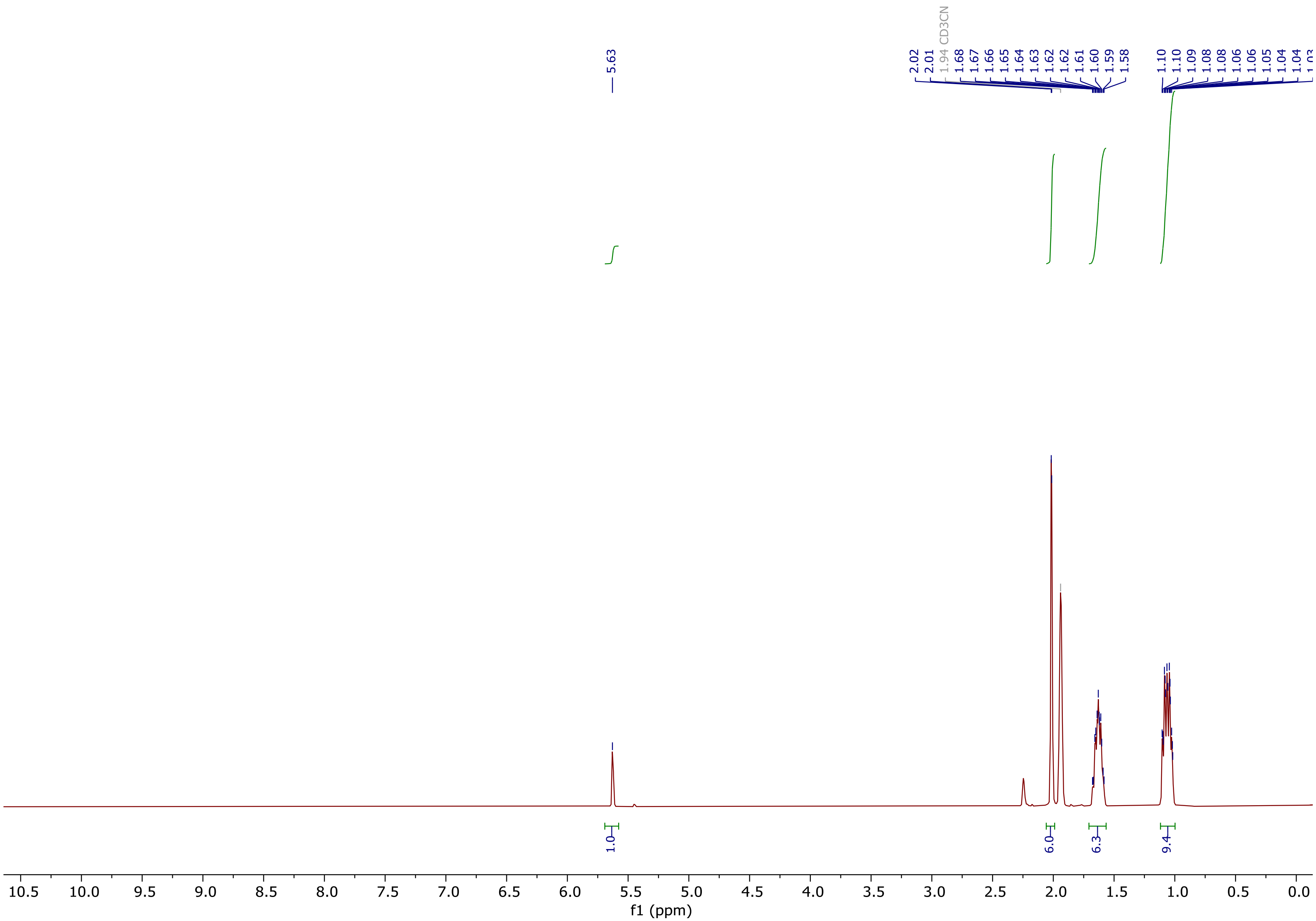

^{19}F NMR (CD_3CN, 376 MHz, 293 K)

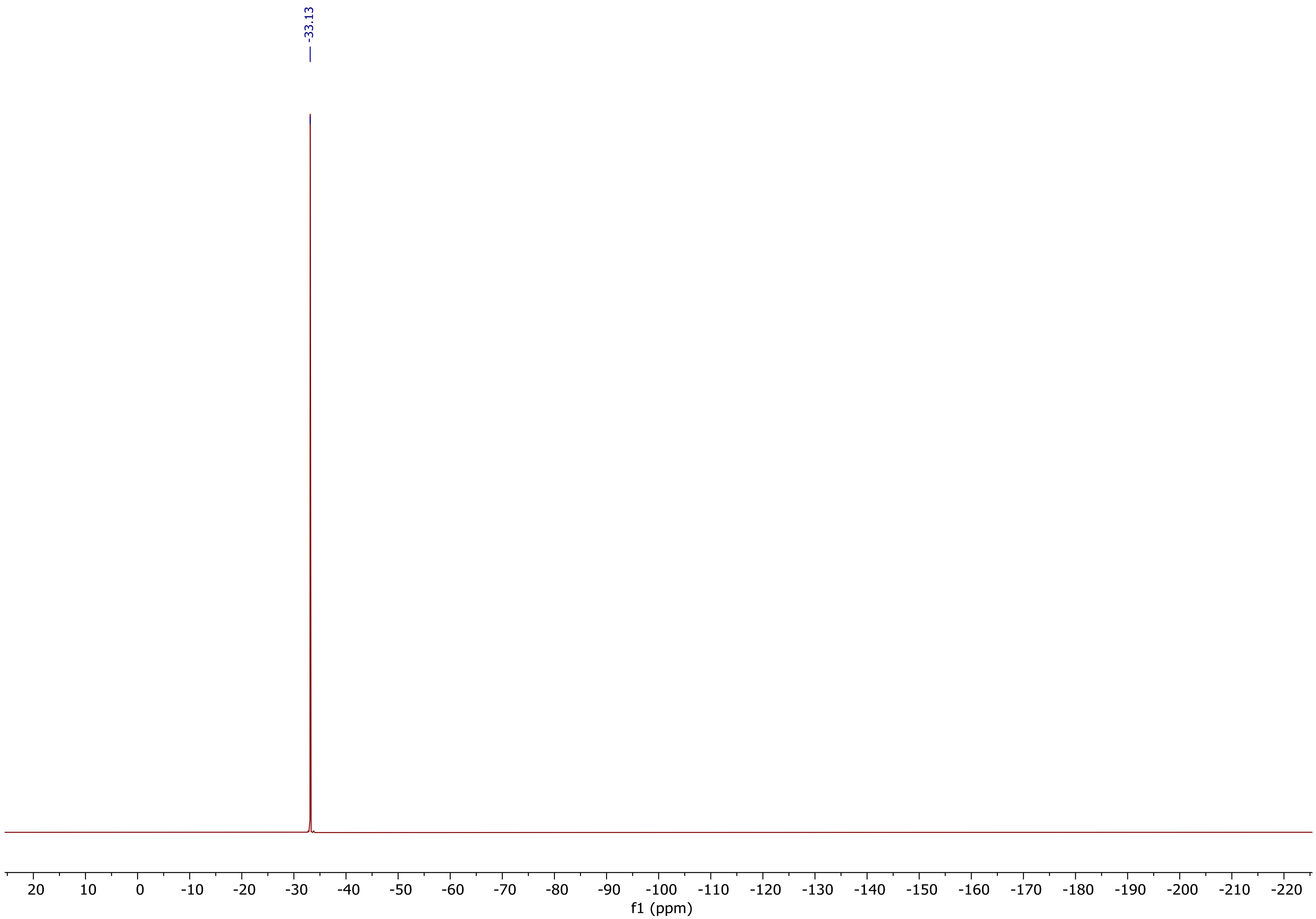

^{31}P NMR (CD_3CN, 162 MHz, 293 K)

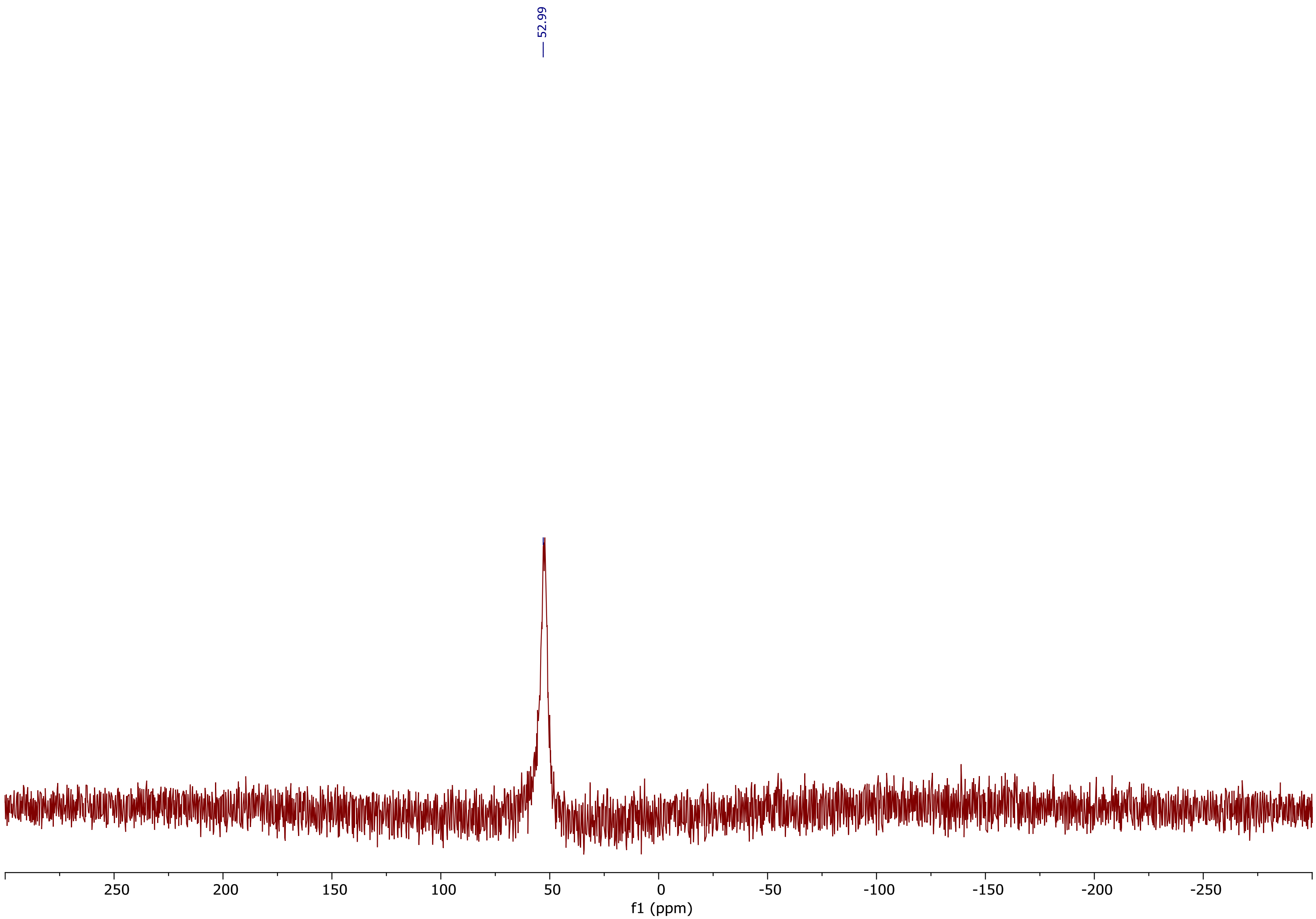

Triethylphosphine oxide (free base)

^{31}P NMR (CD_3CN, 162 MHz, 293 K)

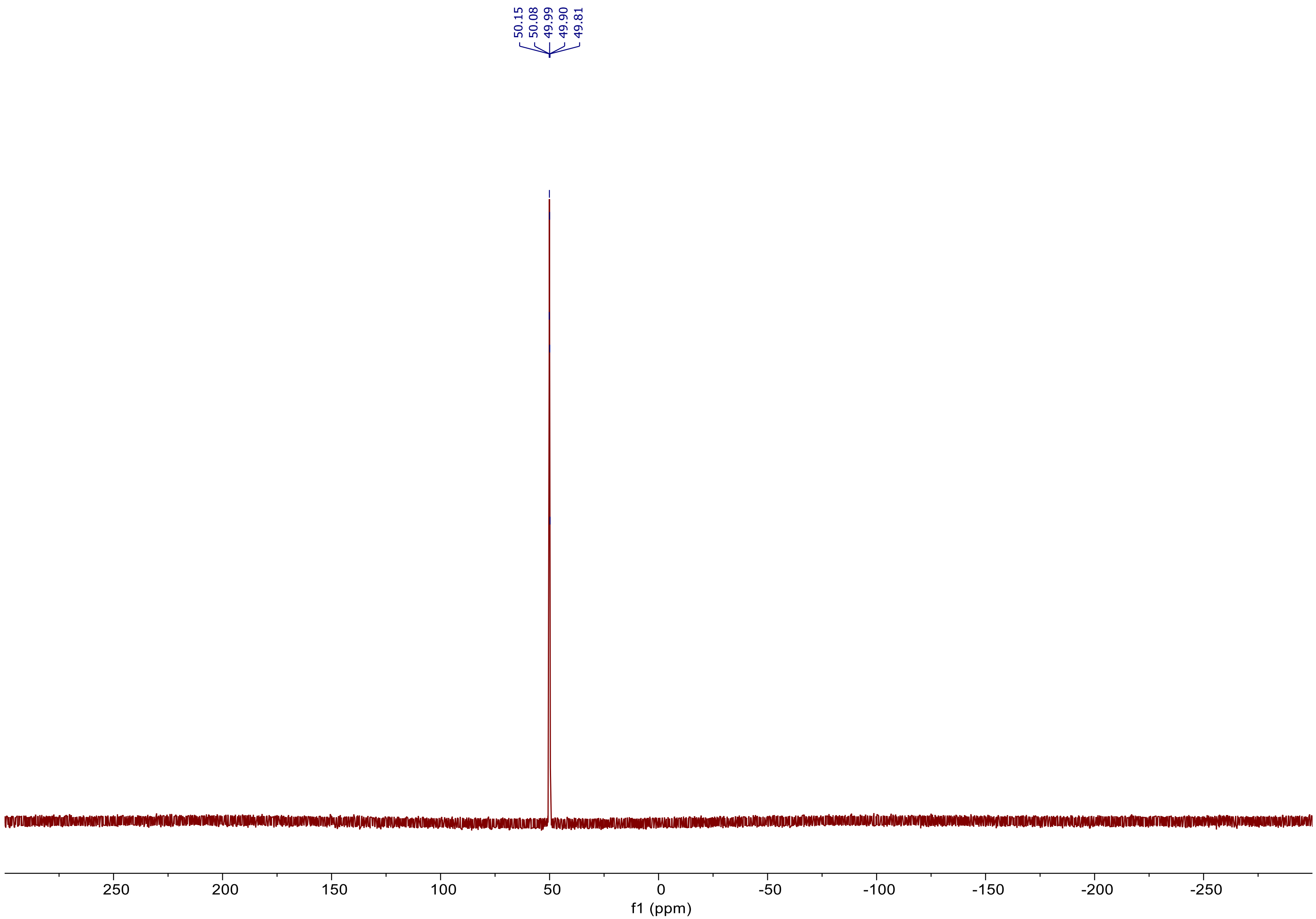

$Bu_4N^+[(acac)Cu(CF_3)_2(Cl)]^-$ 5

^1H NMR ($CDCl_3$, 400 MHz, 293 K)

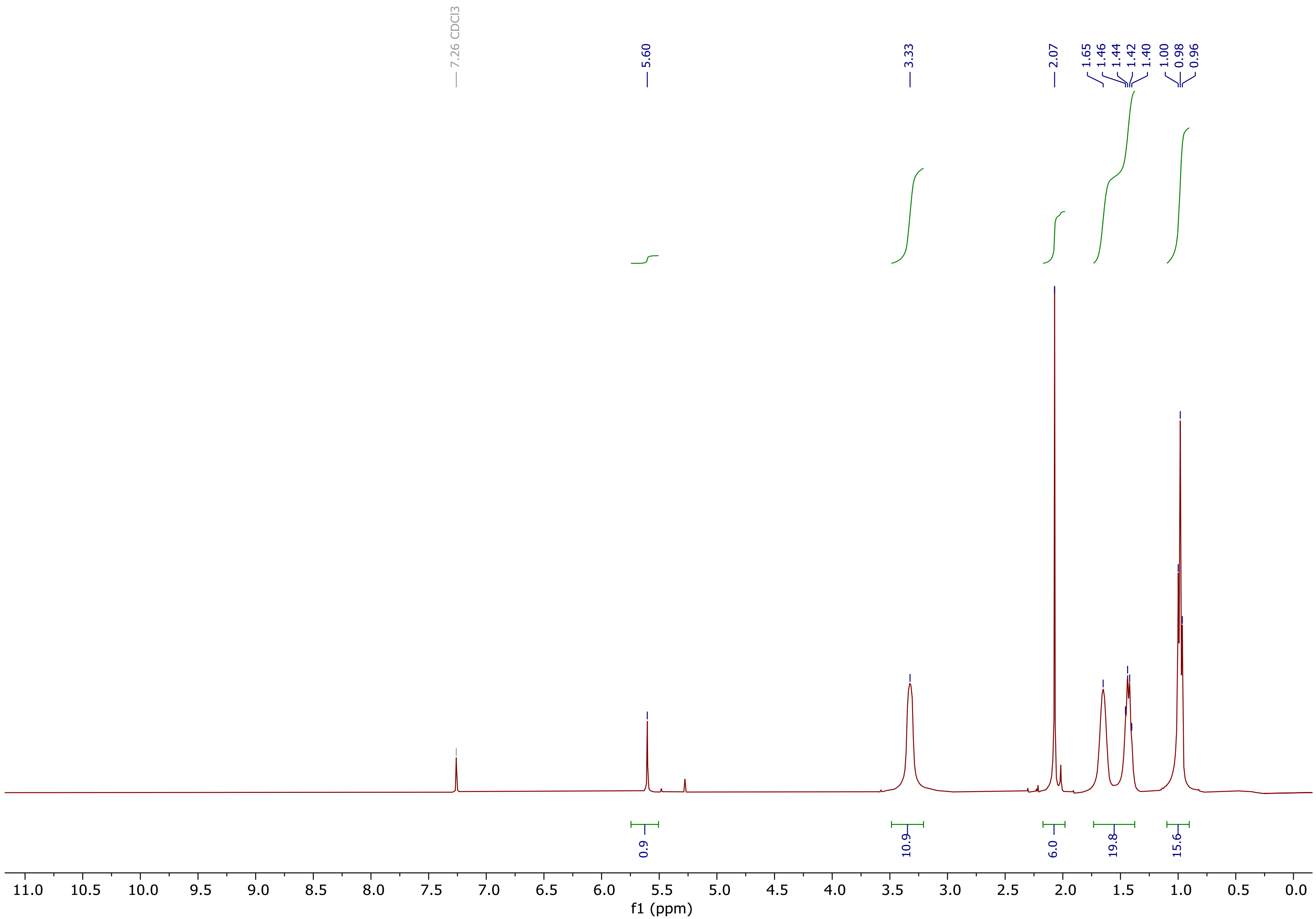

^{13}C NMR ($CDCl_3$, 101 MHz, 293 K)

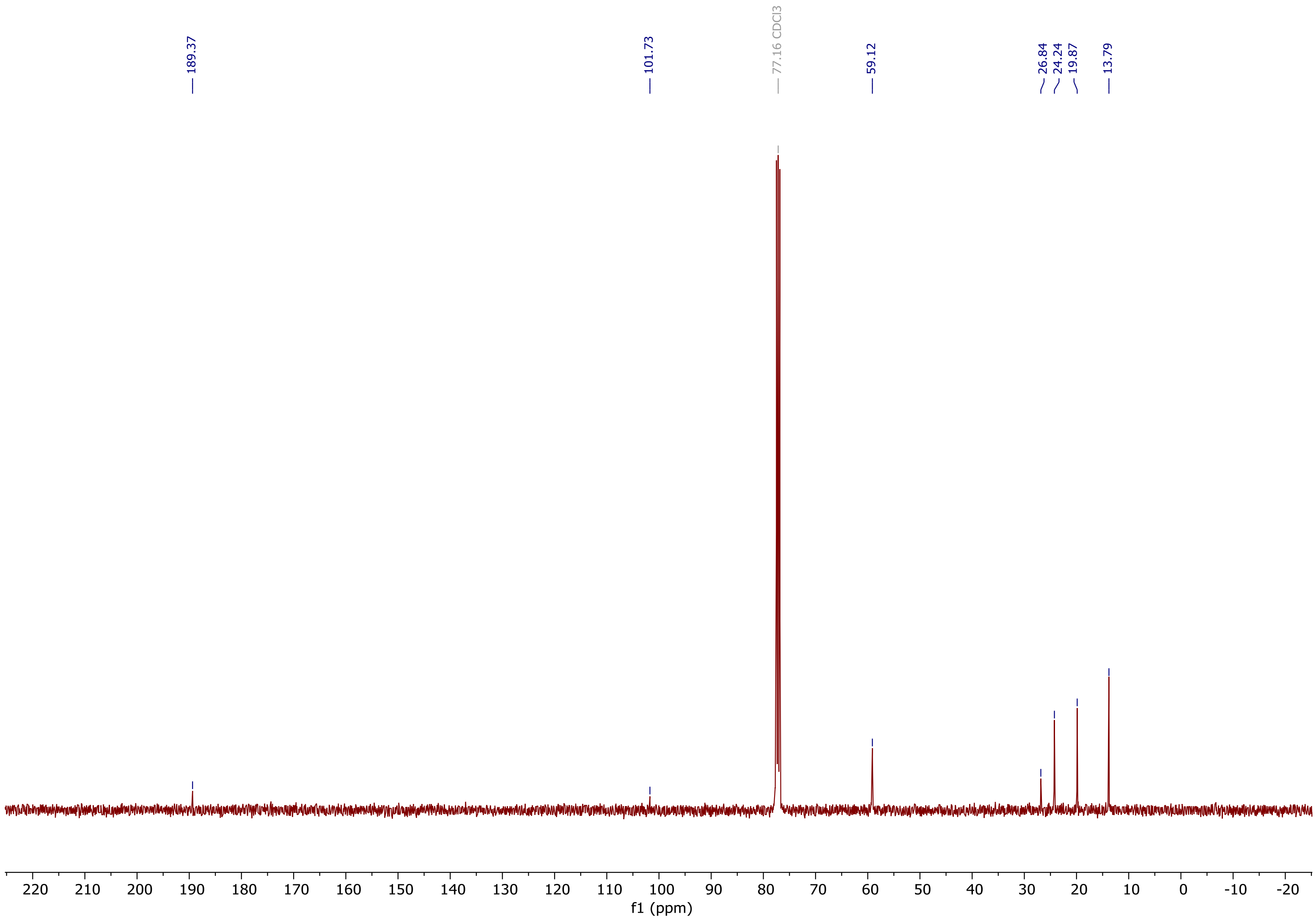

^{19}F NMR ($CDCl_3$, 376 MHz, 293 K)

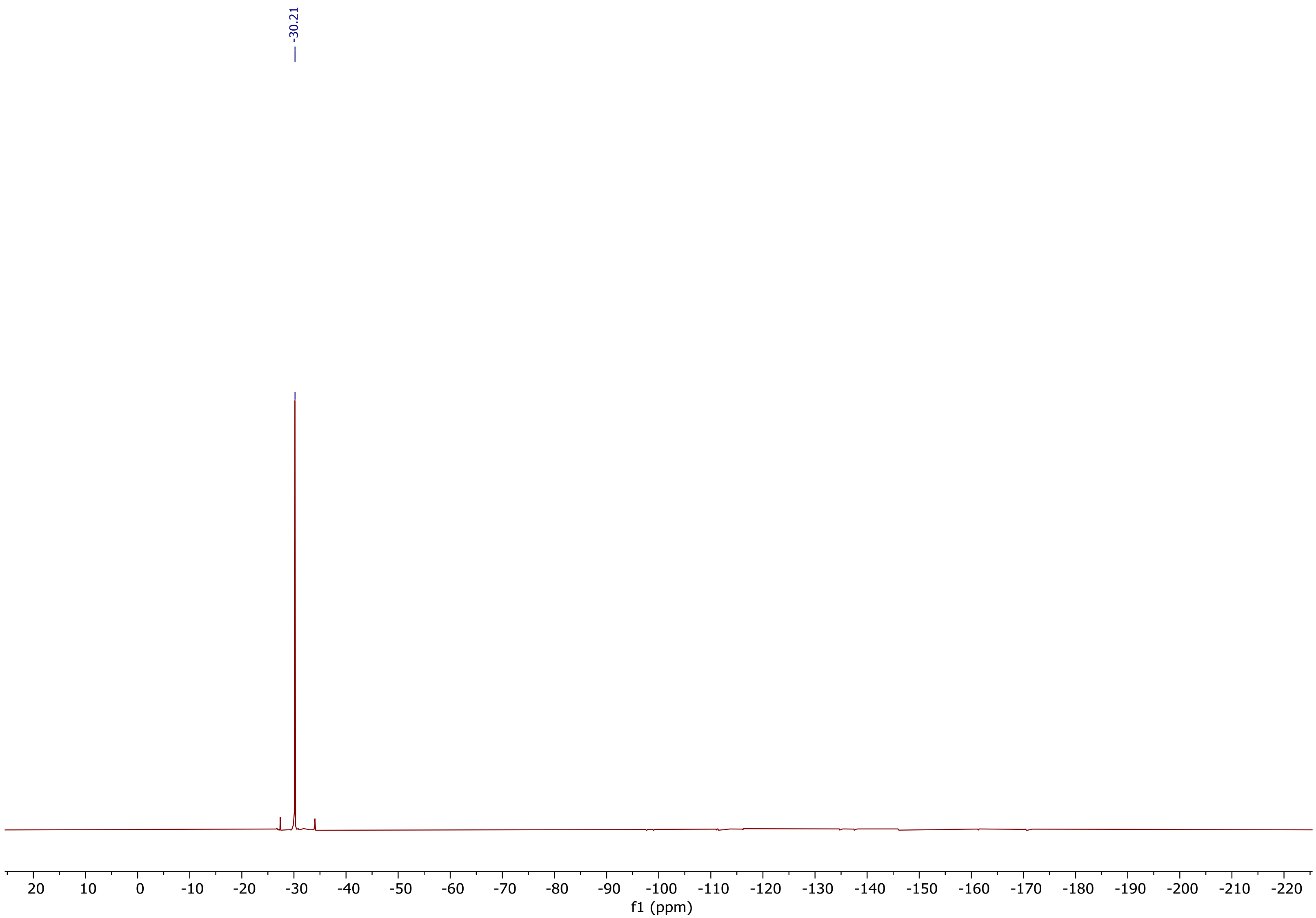

Supplement: Supplementary file 1 [file RA-016-D6RA04158B-s004.pdf]
